# Supplementary material for: Power sector investment implications of climate impacts on renewable resources in Latin America and the Caribbean
Source: Nat Commun. 2021 Feb 24;12:1276. doi: 10.1038/s41467-021-21502-y (PMC7904843; doi:10.1038/s41467-021-21502-y)
Supplement: Supplementary file 1 — Supplementary Information [file 41467_2021_21502_MOESM1_ESM.pdf]

# Supplementary Information:

# Power sector investment implications of climate impacts on renewable resources in Latin America and the Caribbean

Silvia R. Santos da Silva [1,2]*, Mohamad I. Hejazi[2], Gokul Iyer[2], Thomas B. Wild[2,3], Matthew Binsted[2], Fernando Miralles-Wilhelm[1,2,3], Pralit Patel[2], Abigail C. Snyder[2], Chris R. Vernon[4]

[1] Department of Atmospheric and Oceanic Science, University of Maryland, College Park, Maryland, United States of America; [2] Joint Global Change Research Institute, Pacific Northwest National Laboratory, College Park, Maryland, United States of America; [3] Earth System Science Interdisciplinary Center, College Park, Maryland, United States of America; [4] Pacific Northwest National Laboratory, Richland, Washington, United States of America.

* Corresponding author

## Supplementary Figures

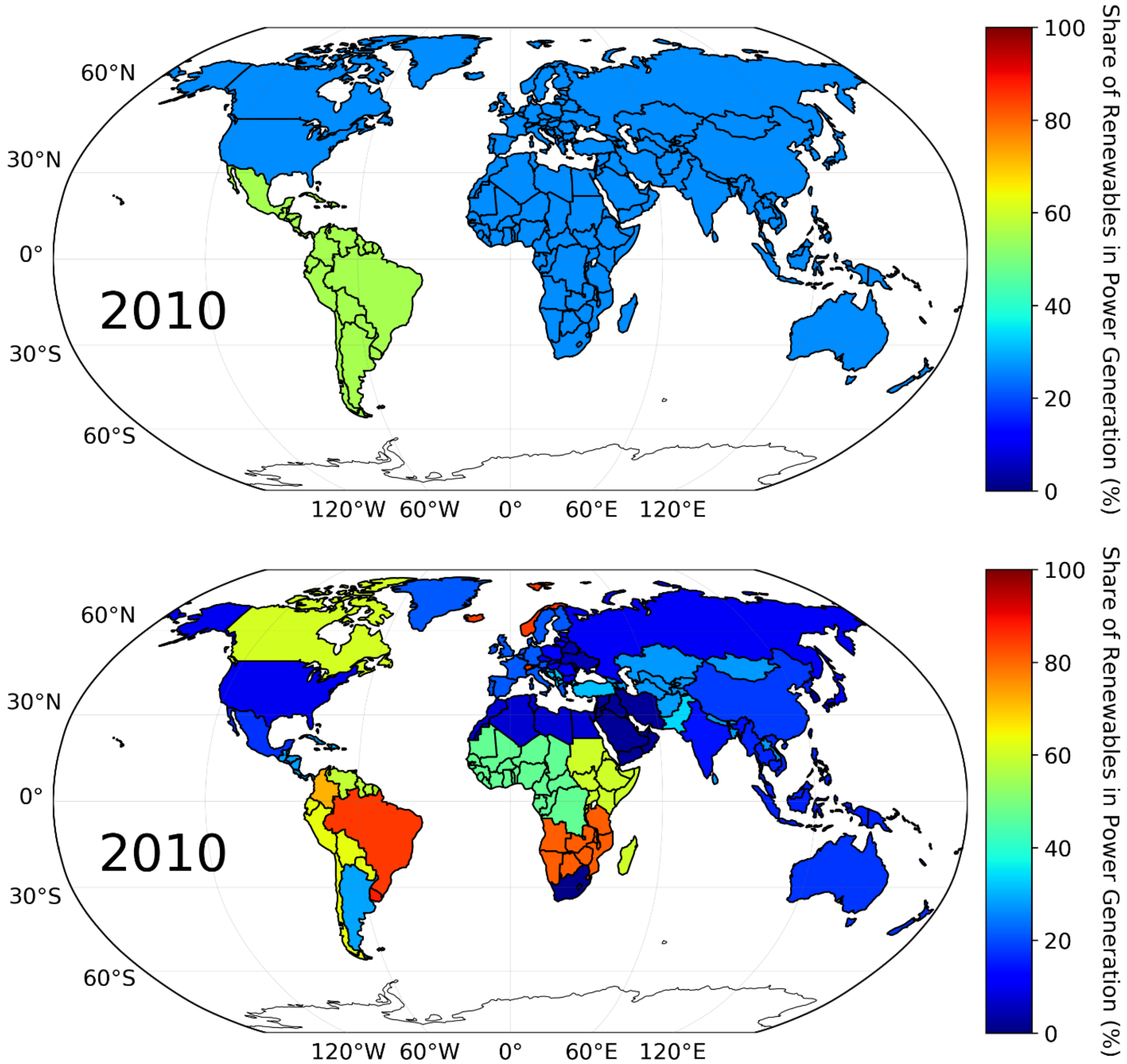


**Supplementary Figure 1.** Share of renewable energy (bioenergy, geothermal, hydropower, solar and wind) in total electricity generation: in the Latin American and the Caribbean (LAC) region compared to the average of the rest of the world (top); and by individual regions (bottom). The share of renewables in power generation was computed as total renewable electricity generation divided by total electricity generation expressed in relative (%) terms. [Notes: (1) 33 geopolitical regions are represented in the GCAM-LAC model version with 8 regions in LAC: Argentina, Brazil, Central America and Caribbean, Colombia, Mexico, South America Northern, South America Southern, and Uruguay; (2) Source: GCAM-LAC total electricity generation by region in 2010 (last calibrated year[1]).]

Supplementary Figure 2 shows the long-term evolution of the electricity generation mix in LAC as projected by the GCAM-LAC *Baseline* (No Policy) scenario. Note that the *Baseline* scenario assumes no greenhouse gas mitigation actions throughout the 21st century. Socioeconomic assumptions are consistent with the Shared Socioeconomic Pathway (SSP) 2, which reflects a world in which social, economic and technological future trends do not differ markedly from historical patterns[2]. However, as noted in the Supplementary Note 2, socioeconomic assumptions in Argentina, Colombia and Uruguay were revised to align with these countries' future projections. Also note that 2010 is the last calibrated year in the GCAM-LAC model[1]. The *Baseline* scenario reflects a substantial increase in electricity generation from 2010 to 2100 (more than five times) resulting from the fast growing regional demand. Fossil-fueled generation (mostly natural gas) becomes progressively more important reaching a share of ~49% of total generation in 2100 (versus ~41% in 2010). Although hydroelectricity production increases in absolute numbers, hydropower loses its role of main regional generation source responding for ~18% of total generation in 2100 (versus ~52% in 2010). Solar and wind technologies become more important over time collectively responding for ~21% of LAC's total generation in 2100. Supplementary Figure 3 shows similar electricity generation outputs by GCAM-LAC region.

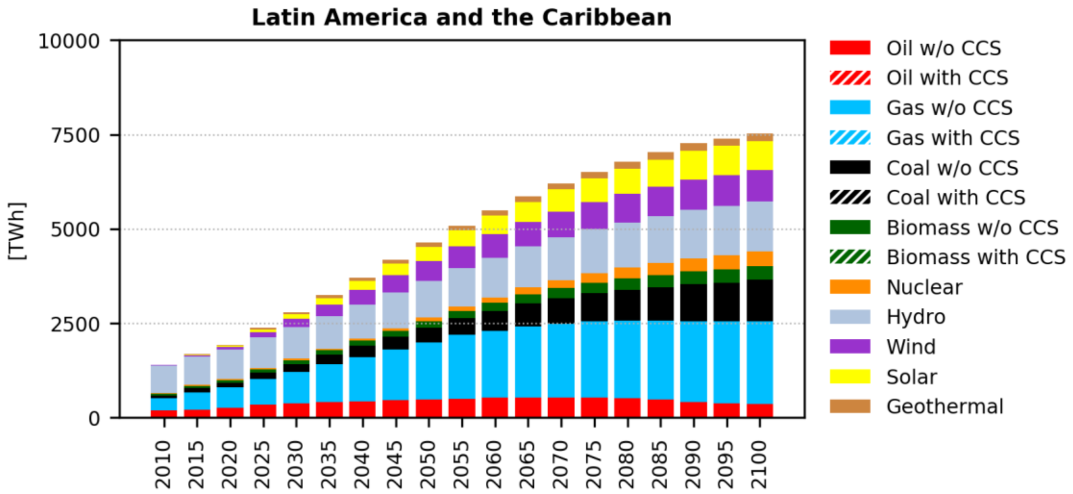


**Supplementary Figure 2.** Electricity generation by technology in the GCAM-LAC *Baseline (No Policy)* scenario. (Note that this scenario is identical to the *RCP60_Baseline: No-climate impacts* scenario mentioned in the Table 1 of the main text.)

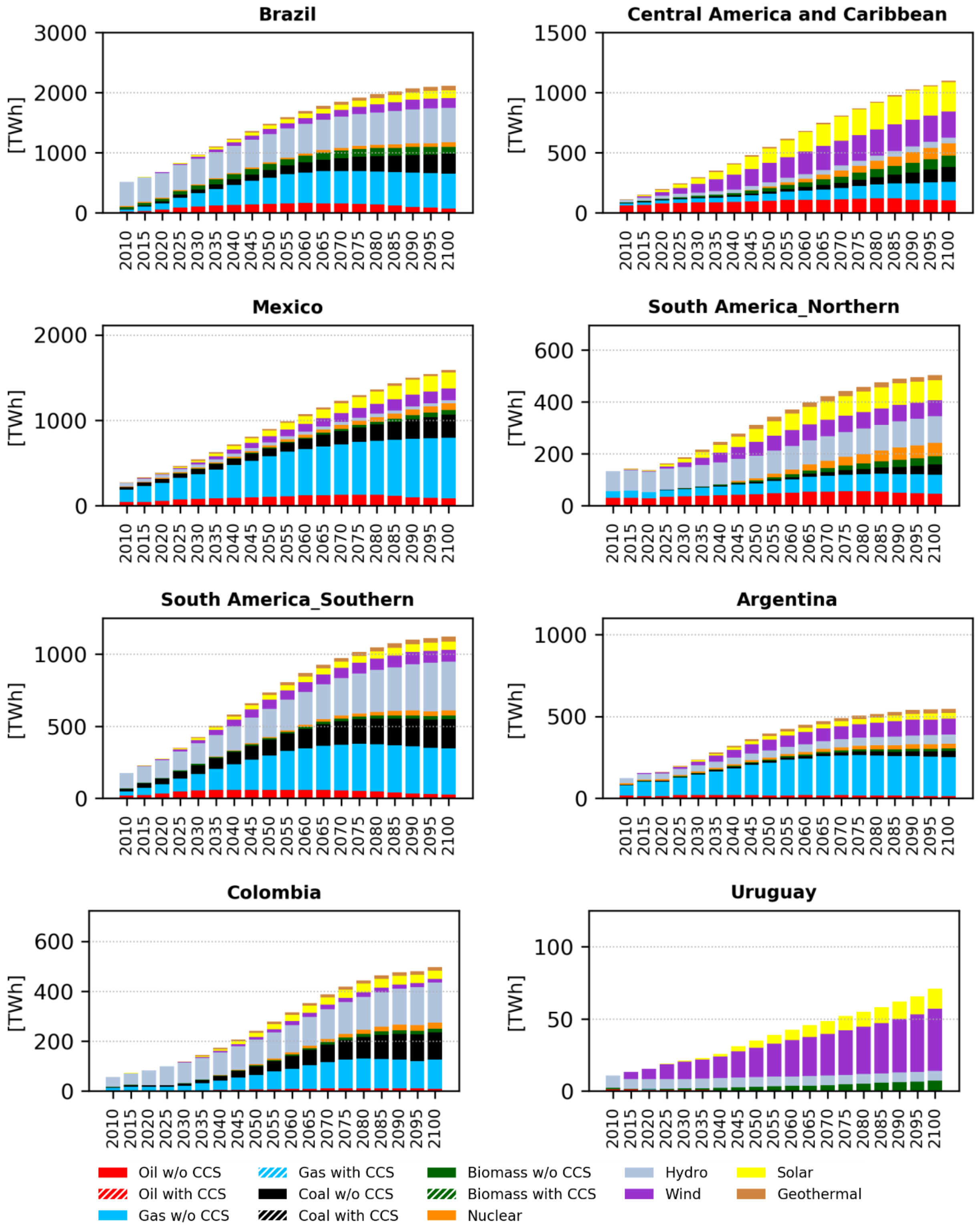


**Supplementary Figure 3.** Electricity generation by technology in the GCAM *Baseline (No Policy)* scenario for the eight LAC regions represented in GCAM-LAC. (Note that this scenario is identical to the *RCP60_Baseline: No-climate impacts* scenario mentioned in the Table 1 of the main text.)

Supplementary Figure 4 contrasts the long-term evolution of the electricity generation mix in LAC as projected by the *RCP60_Baseline* scenario and the two *RCP26* mitigation scenarios implemented in this study. As noted in the main text, the mitigation scenarios vary with respect to technology availability assumptions (see Table 1 in the main text). Although the mitigation scenarios defined in the main text also vary in assumptions about climate impacts on renewables, Supplementary Figure 4 focuses on the "No-climate impacts" assumptions. It is important to start by the "No-climate impacts" scenario outcomes because all climate-impact scenarios defined for this study are based on them, and because they serve as the benchmark for all comparisons (i.e., each scenario with climate-impacts are compared against its "No-climate impacts" counterpart). The role of the energy technology scenarios in this study is to provide long-term energy pathways with varying degrees of low-carbon technology deployments. Under these circumstances, power sector implications from climate impacts on the renewable supply may differ markedly. Supplementary Figures 5–6 show similar electricity generation outputs by GCAM-LAC region.

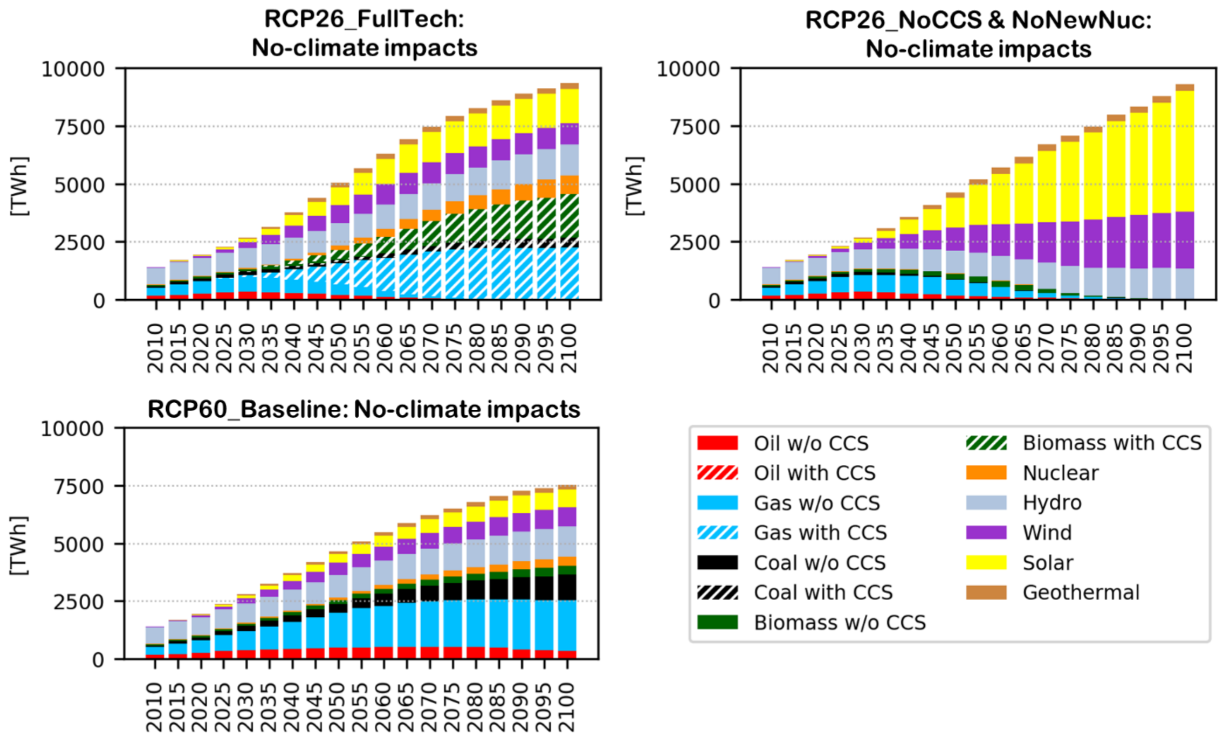


**Supplementary Figure 4.** Electricity generation by technology in the *RCP26_FullTech: No-climate impacts* scenario (top left), *RCP26_NoCCS & NoNewNuc: No-climate impacts* scenario (top right), and *RCP60_Baseline: No-climate impacts* scenario (bottom left) in LAC.

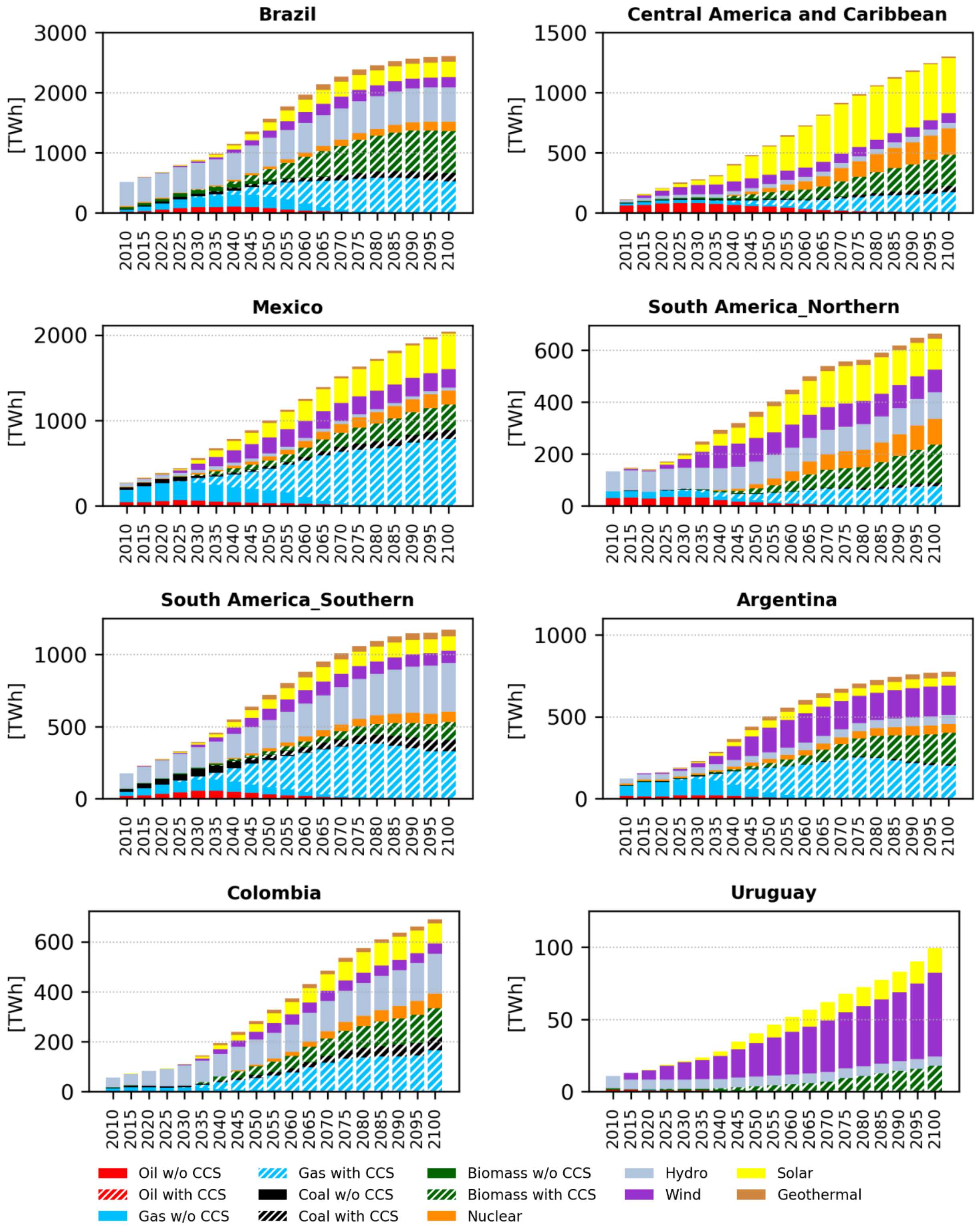


**Supplementary Figure 5.** Electricity generation by technology in the *RCP26_FullTech: No-climate impacts* scenario for the eight LAC regions represented in GCAM-LAC.

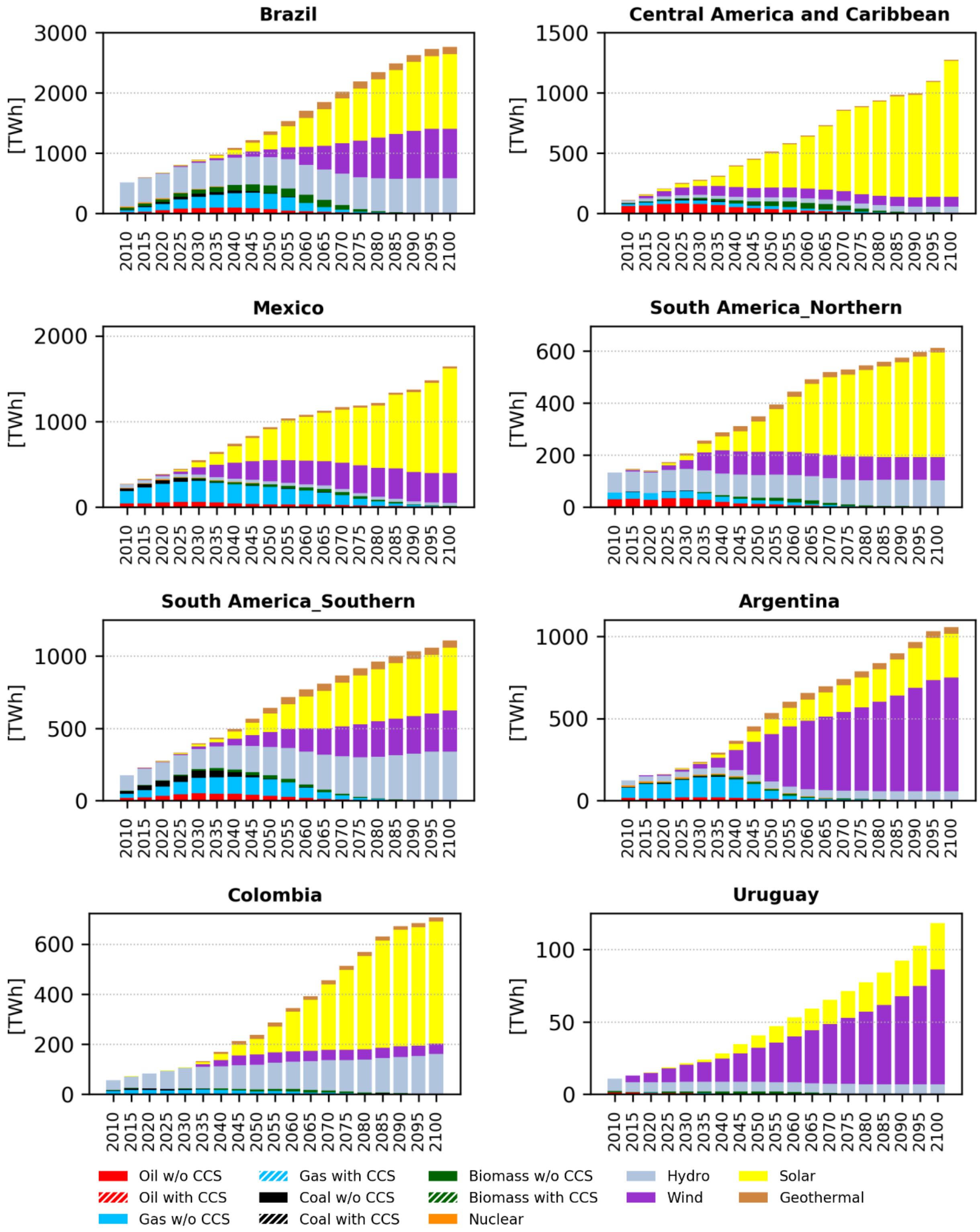


**Supplementary Figure 6.** Electricity generation by technology in the *RCP26_NoCCS & NoNewNuc: No-climate Impacts* scenario for the eight LAC regions represented in GCAM-LAC.

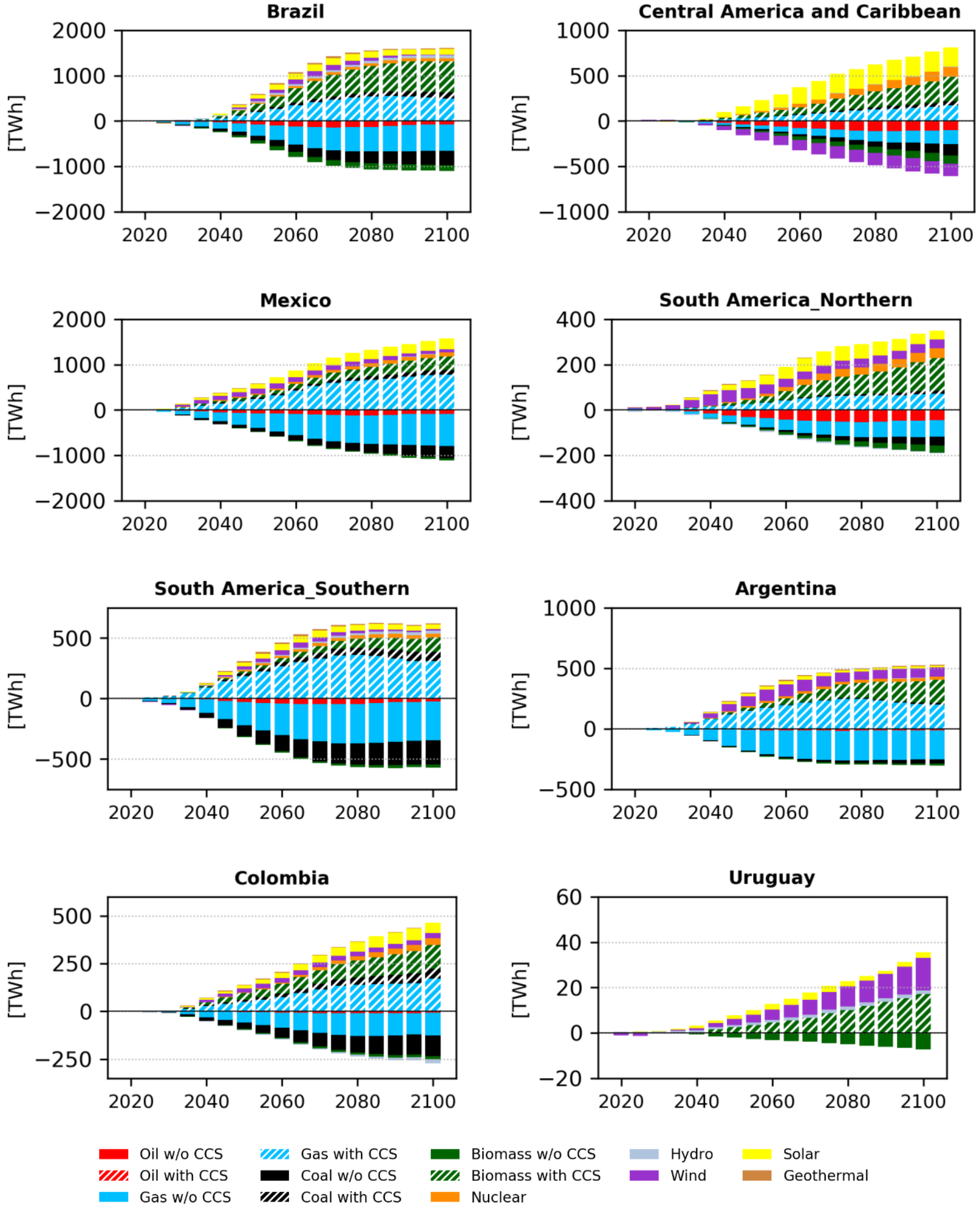


**Supplementary Figure 7.** Changes in electricity generation under the *RCP26_FullTech: Combined impacts* scenario relative to the GCAM *Baseline* scenario (which is identical to the *RCP60_Baseline: No-climate impacts* scenario).

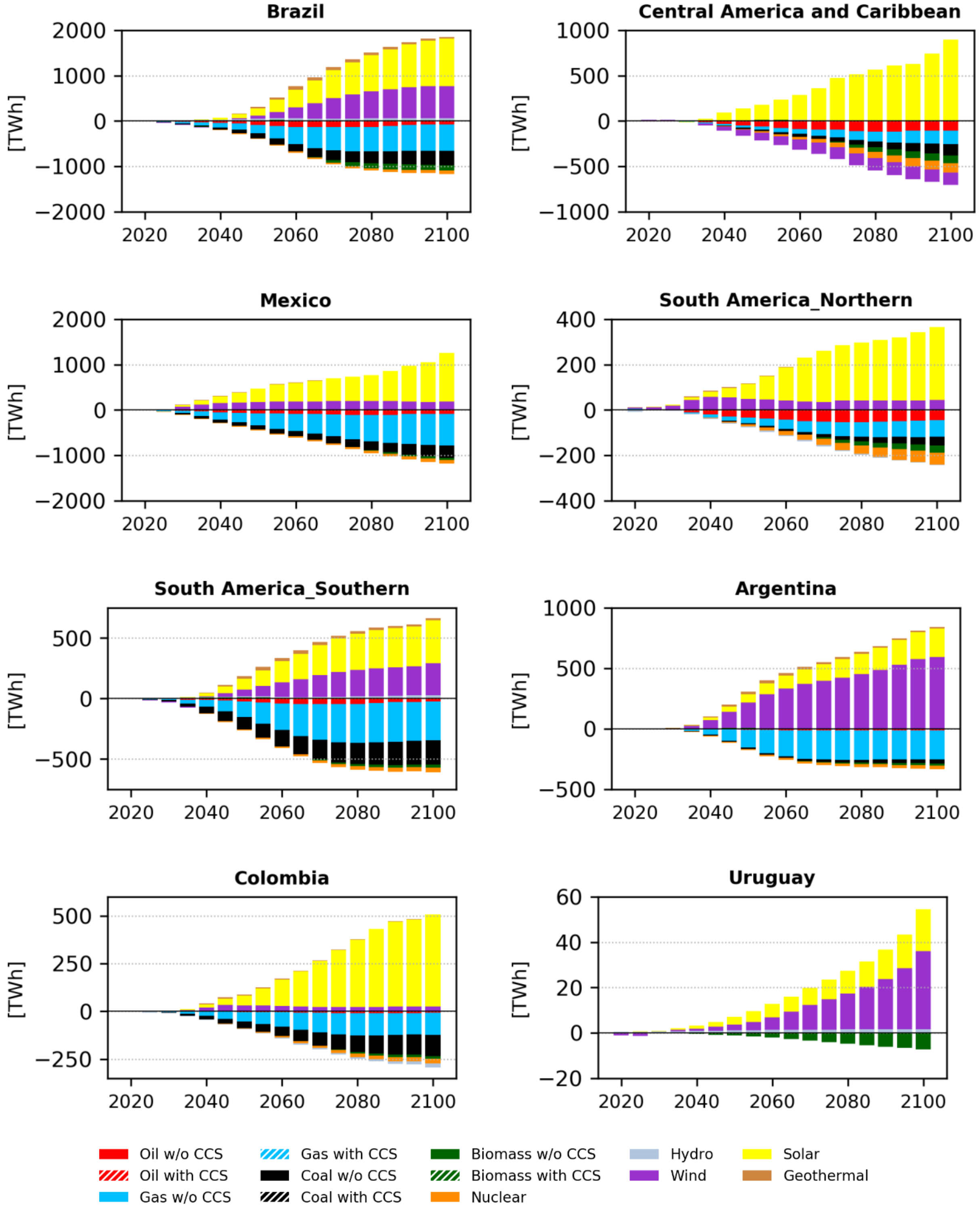


**Supplementary Figure 8.** Changes in electricity generation under the *RCP26_NoCCS & NoNewNuc: Combined impacts* scenario relative to the GCAM *Baseline* scenario (which is identical to the *RCP60_Baseline: No-climate impacts* scenario).

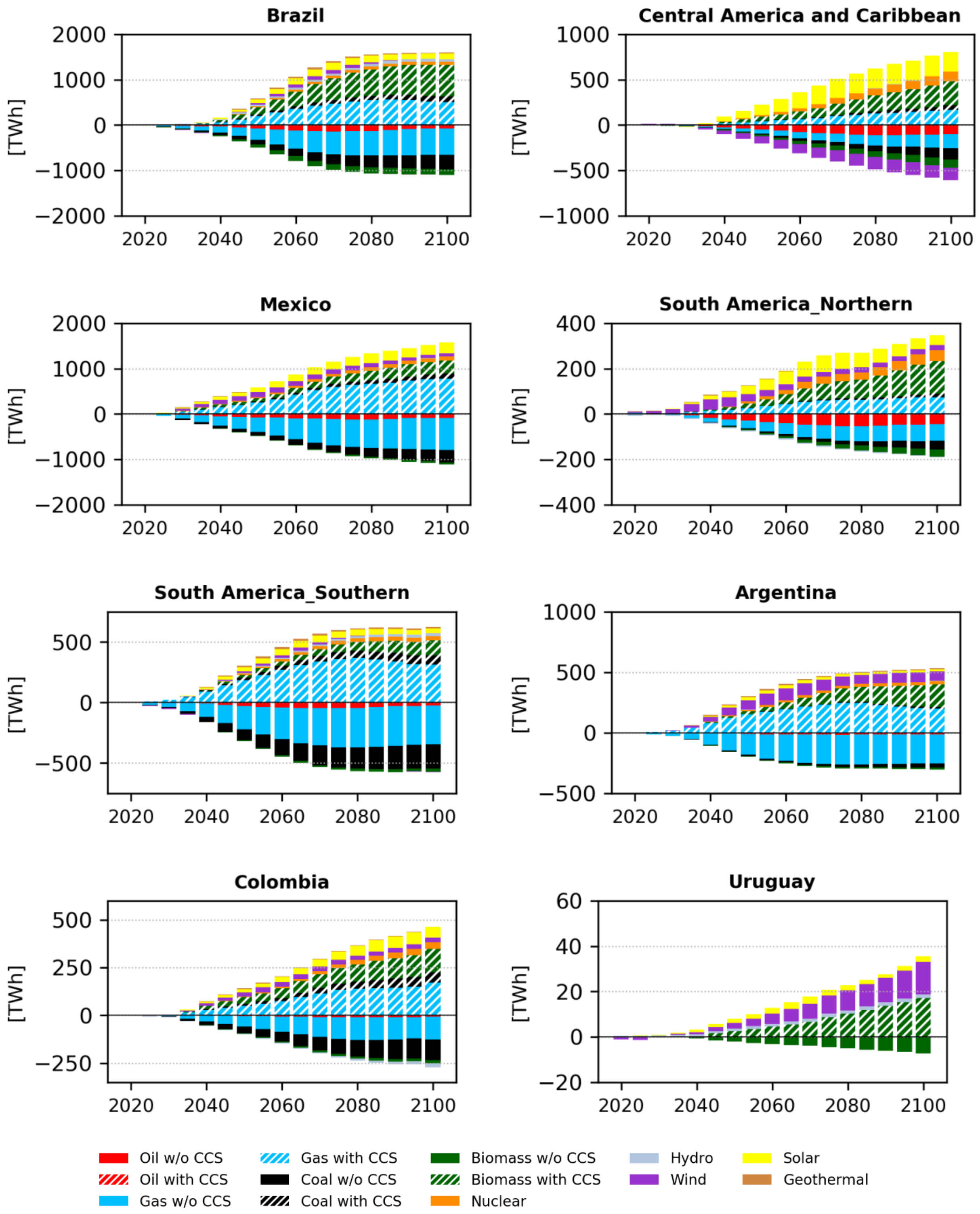


**Supplementary Figure 9.** Changes in electricity generation under the RCP26_*FullTech: Hydropower* scenario relative to the GCAM *Baseline* scenario (which is identical to the *RCP60_Baseline: No-climate impacts* scenario).

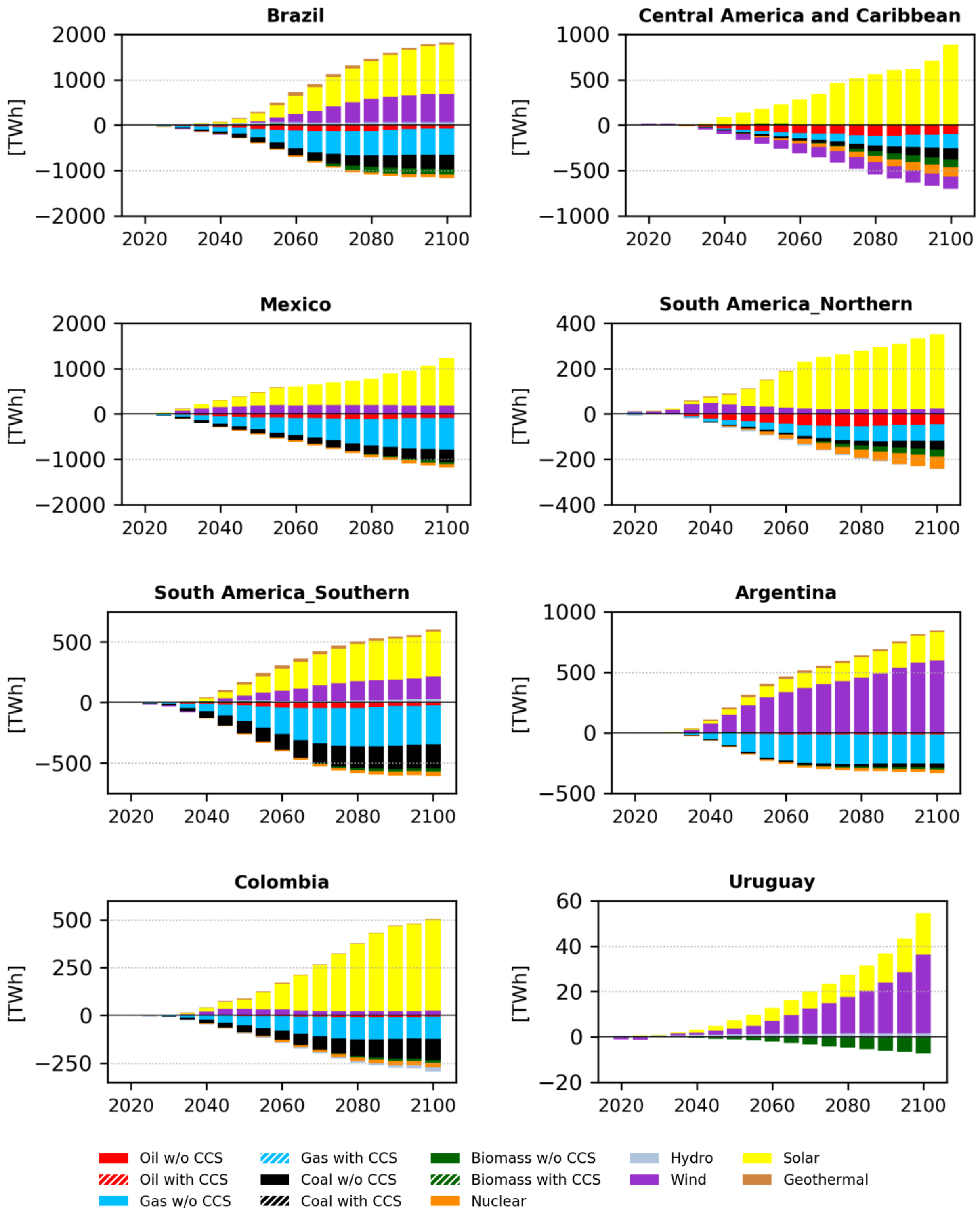


**Supplementary Figure 10.** Changes in electricity generation under the *RCP26_NoCCS & NoNewNuc: Hydropower* scenario relative to the GCAM *Baseline* scenario (which is identical to the *RCP60_Baseline: No-climate impacts* scenario).

Supplementary Figures 11–14 are discussed within the context of Supplementary Note 6. These figures contrast the direct effect of incorporating each RE climate-impact representation in GCAM individually (left panels) against the compounding effects of incorporating multiple climate impacts simultaneously (center panels). In response to the climate-impact representations incorporated in GCAM, changes in hydropower and wind-based generation are the most pronounced across LAC while changes in biomass and solar-based generation are mostly indirect responses to the changes induced by hydropower and solar (see Supplementary Note 6).

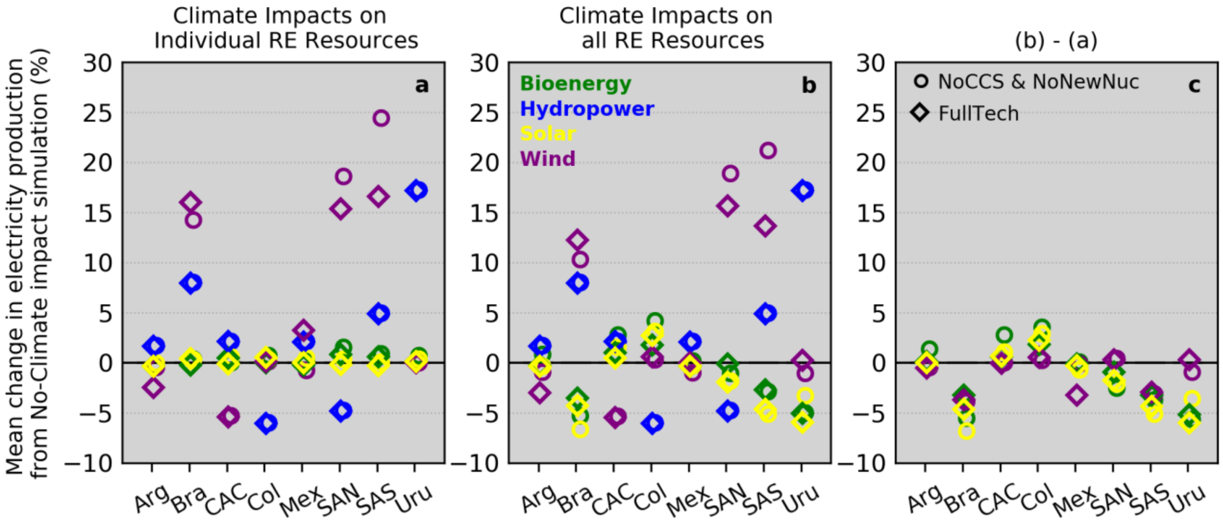


**Supplementary Figure 11.** Mean changes in electricity generation in LAC assuming climate change impacts on renewables. Changes represent the mean value across GCMs, and are calculated by technology scenario (defined in the main text; labelled in **c**) and RE generating source (labelled in **b**) using cumulative generation in the 2020 — 2100 period. Percent changes are relative to the corresponding *No-climate impacts* simulations (positive values indicate that scenarios with climate impacts on renewables show higher cumulative generation). **a**. Assumption of climate impacts on each individual renewable source separately. **b**. Assumption of climate impacts on all renewables (*Combined impacts* scenarios in Table 1 of the main text). **c**. Differences between outputs in **b** and **a** (hydropower is not plotted since the temporal evolution of hydroelectricity production per GCAM region is exogenously predetermined, i.e., fixed; thus differences between scenarios **a** and **b** are zero). GCAM LAC regions covered: Argentina (Arg), Brazil (Bra), Central America and the Caribbean (CAC), Mexico (Mex), South America_Northern (SAN), South America_Southern (SAS), Colombia (Col) and Uruguay (Uru) (Supplementary Table 4 provides a breakdown of countries per GCAM LAC region).

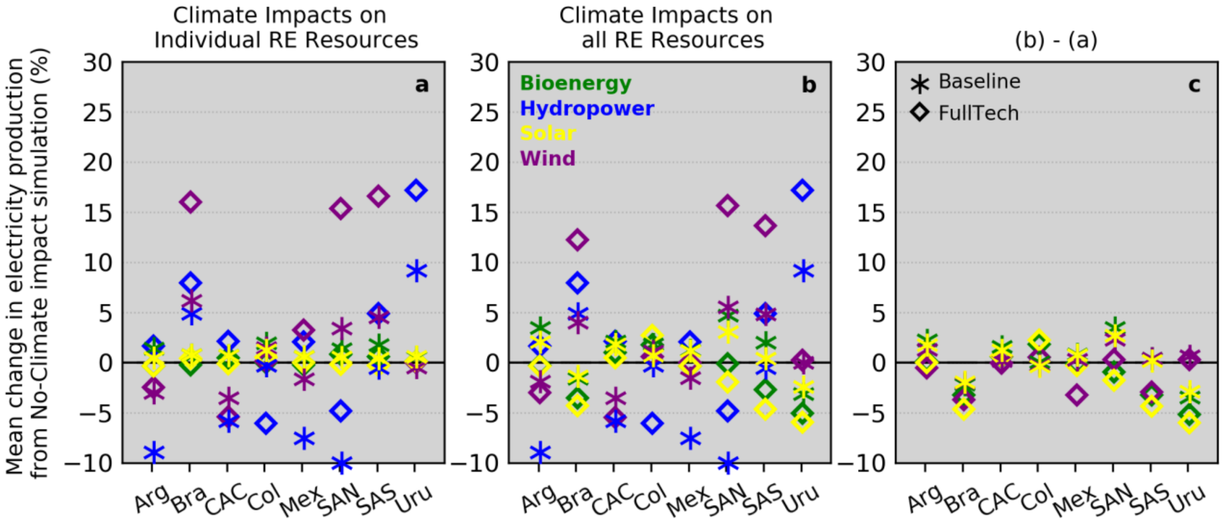


**Supplementary Figure 12.** As in Supplementary Figure 11 but showing results for the *Baseline* and *FullTech* scenarios.

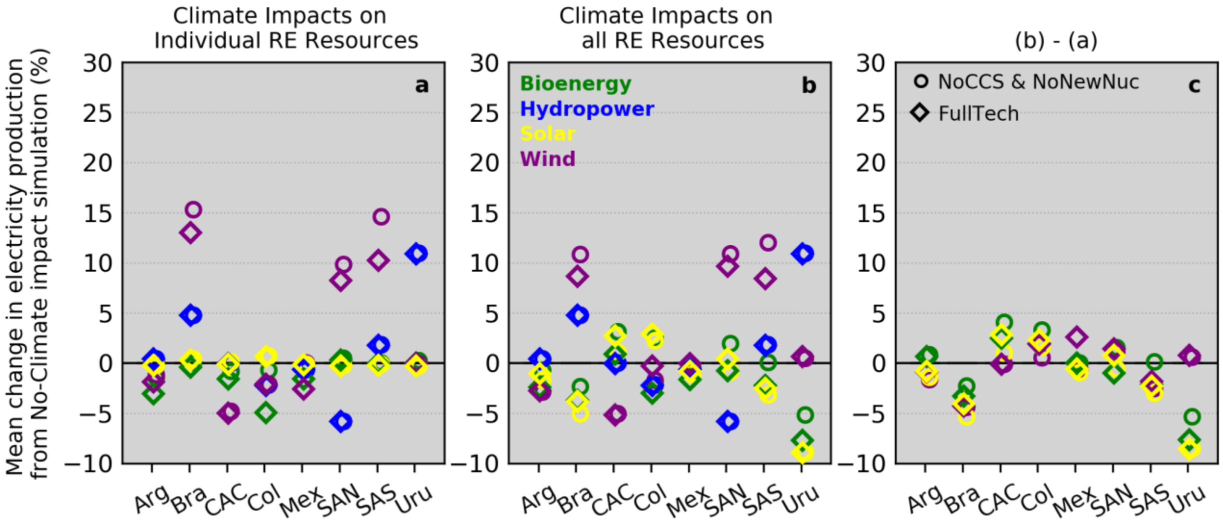


**Supplementary Figure 13.** As in Supplementary Figure 11 but for the 2020-2050 period.

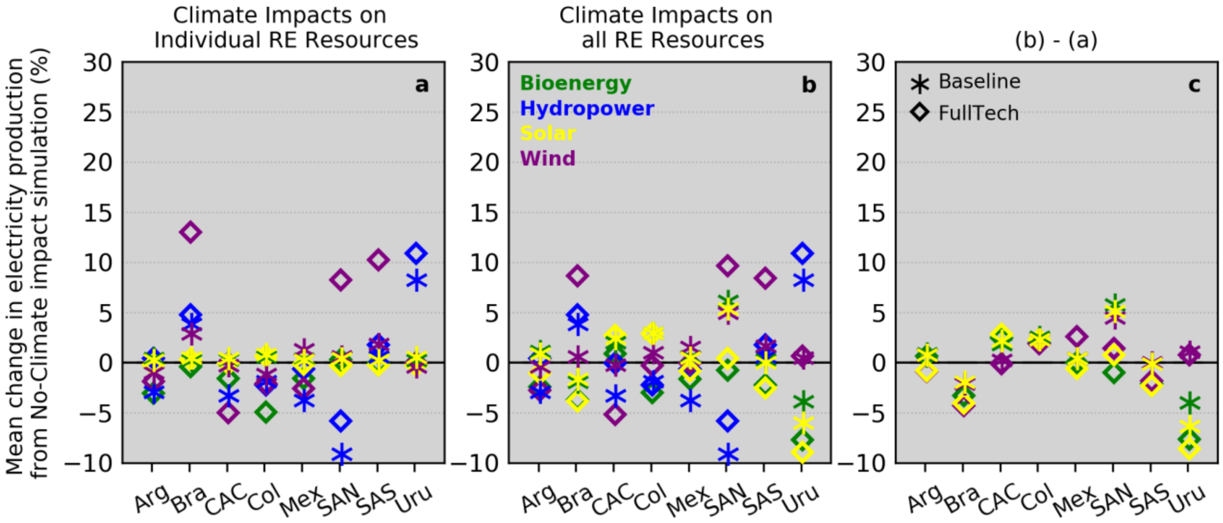


**Supplementary Figure 14.** As in Supplementary Figure 12 but for the 2020-2050 period.

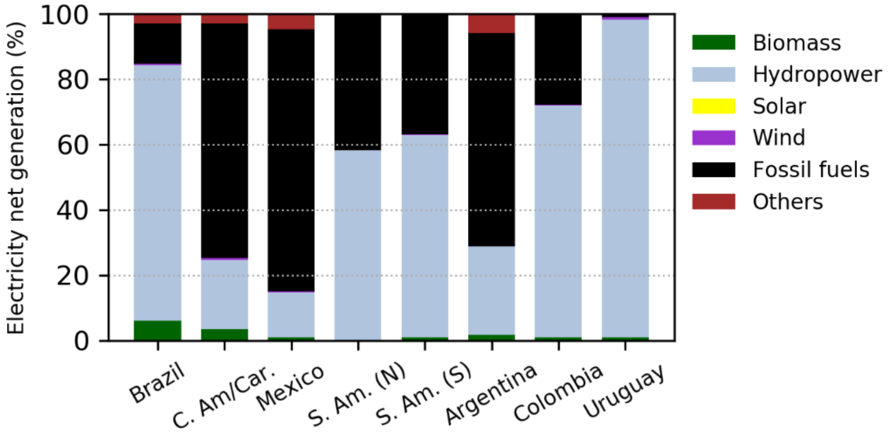


**Supplementary Figure 15.** Contribution of distinct electricity generating sources to total electricity generation per GCAM-LAC region, calculated on the basis of data from the GCAM-LAC model in the year 2010 (last calibrated year[1]).

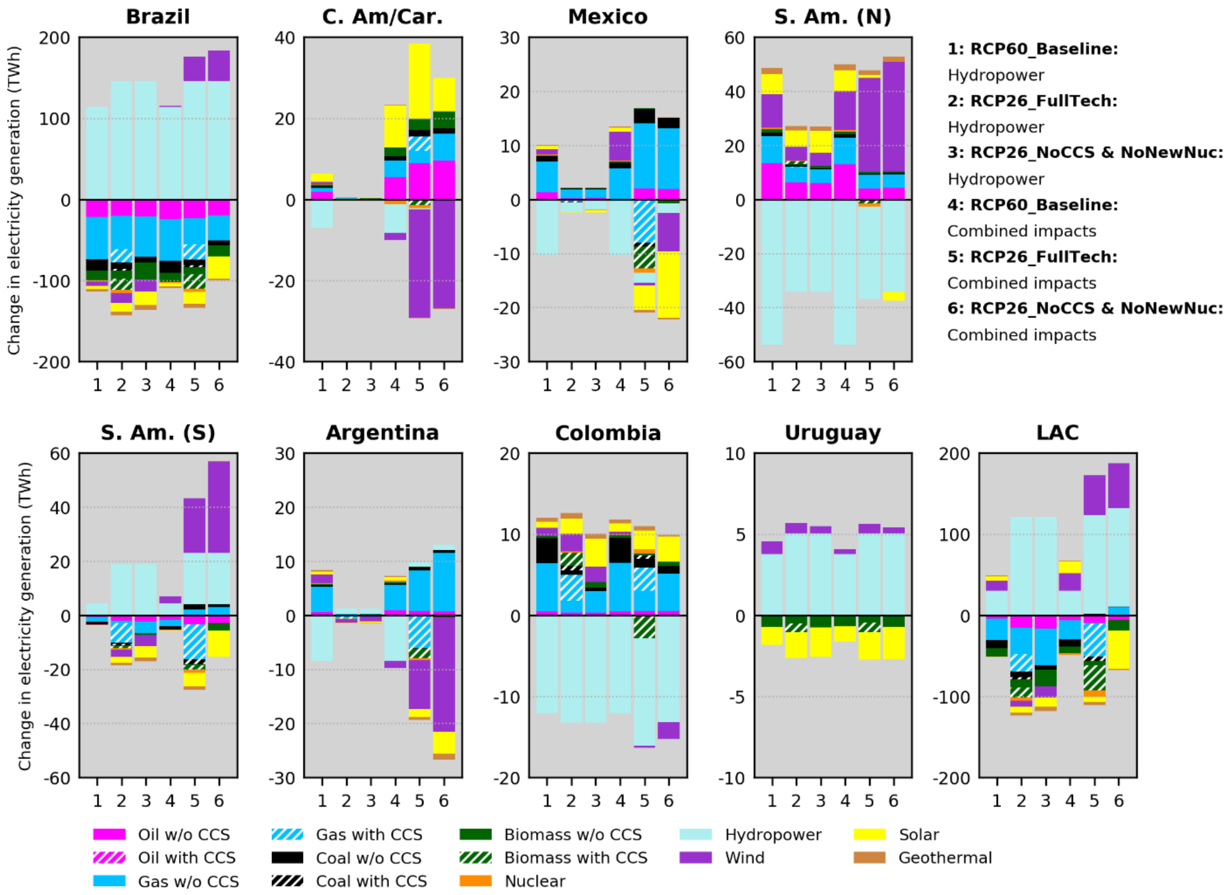


**Supplementary Figure 16.** Model mean differences in electricity production by technology in LAC assuming climate change impacts on renewables. Differences are calculated by technology using cumulative electricity generation (Terawatt-hours – TWh) during the 2020 – 2050 period and are relative to the corresponding *No-Climate impacts* scenarios. LAC regions covered include Brazil, Central America and the Caribbean (C. Am/Car.), Mexico, South America_Northern (S. Am. (N)), South America_Southern (S. Am. (S)), Argentina, Colombia and Uruguay (Supplementary Table 4 provides a breakdown of countries per GCAM LAC region). Note the different y axis scales across regions, and that the y axis scales do not match those presented in Fig. 1 of the main text.

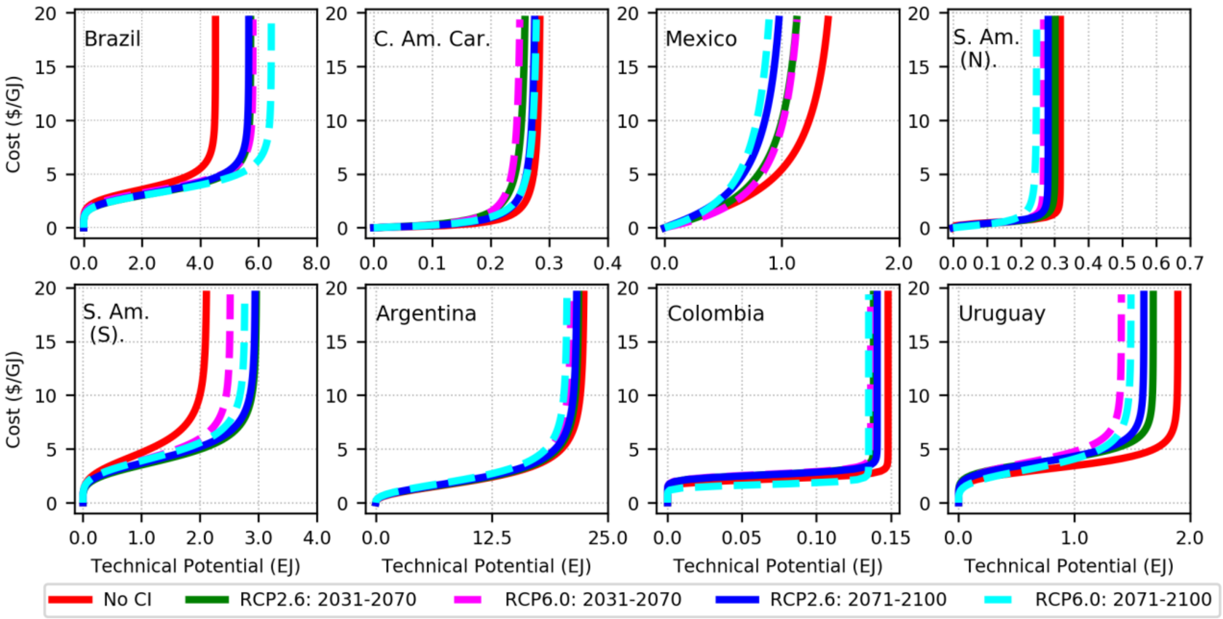


**Supplementary Figure 17.** Latin America and Caribbean cost-supply curves for wind energy using climate inputs from the GFDL-ESM2M model under RCPs 2.6 and 6.0. Cost-supply curves for three different periods were implemented in GCAM: two curves representing future climate states and one curve produced from data corresponding to the model historical period (labeled "No CI").

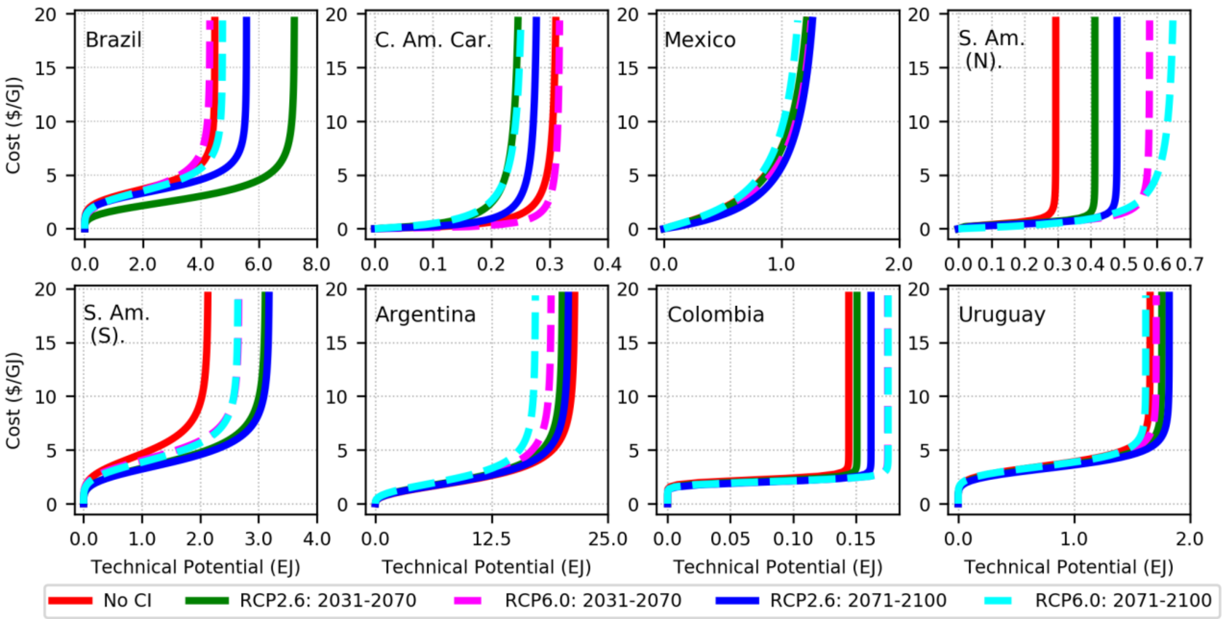


**Supplementary Figure 18.** As in Supplementary Figure 17 but using climate inputs from the HadGEM2-ES model.

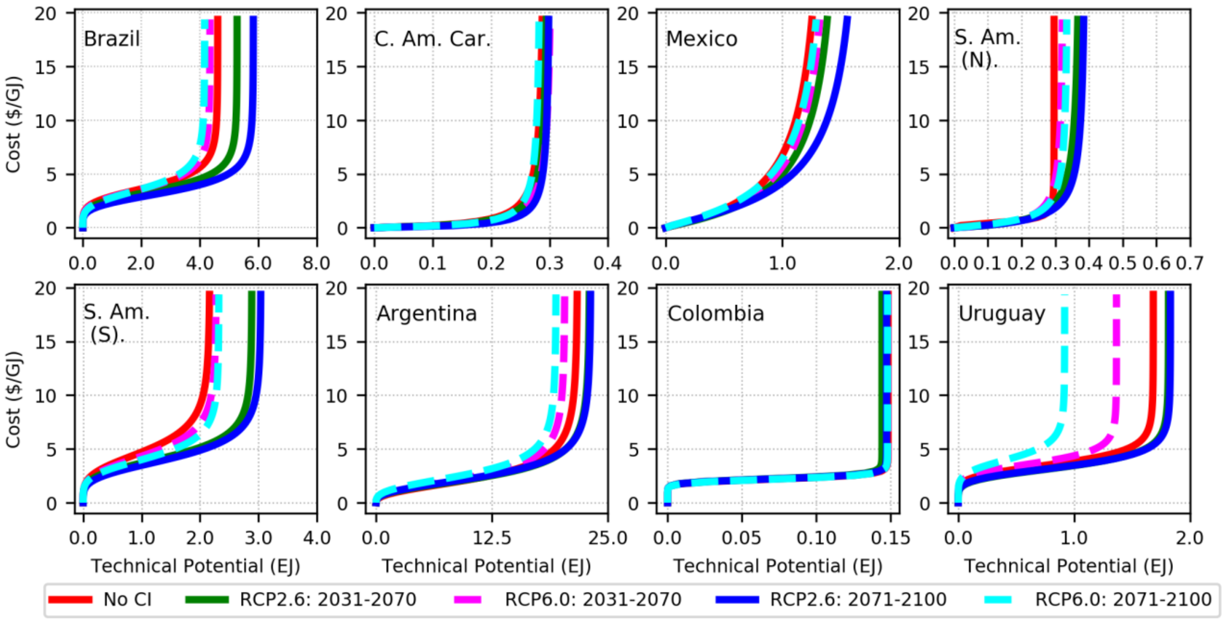


**Supplementary Figure 19.** As in Supplementary Figure 17 but using climate inputs from the IPSL-CM5A-LR model.

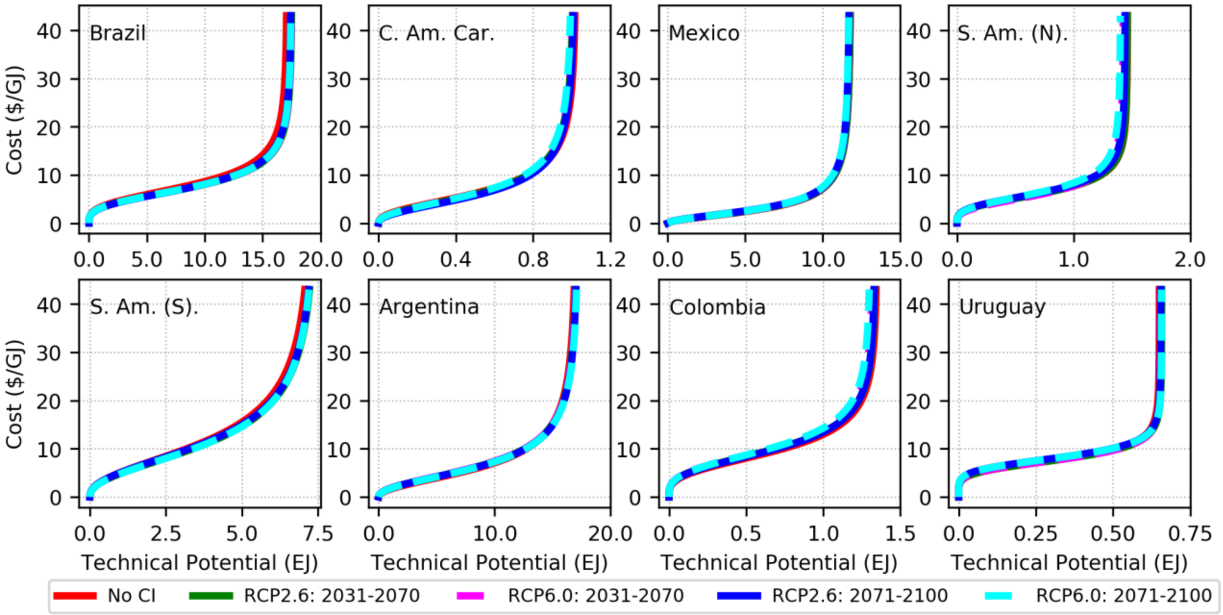


**Supplementary Figure 20.** Latin America and Caribbean cost-supply curves for solar (PV) energy using climate inputs from the GFDL-ESM2M model under RCPs 2.6 and 6.0. Cost-supply curves for three different periods were implemented in GCAM: two curves representing future climate states  and one curve produced from data corresponding to the model historical period (labeled "No CI").

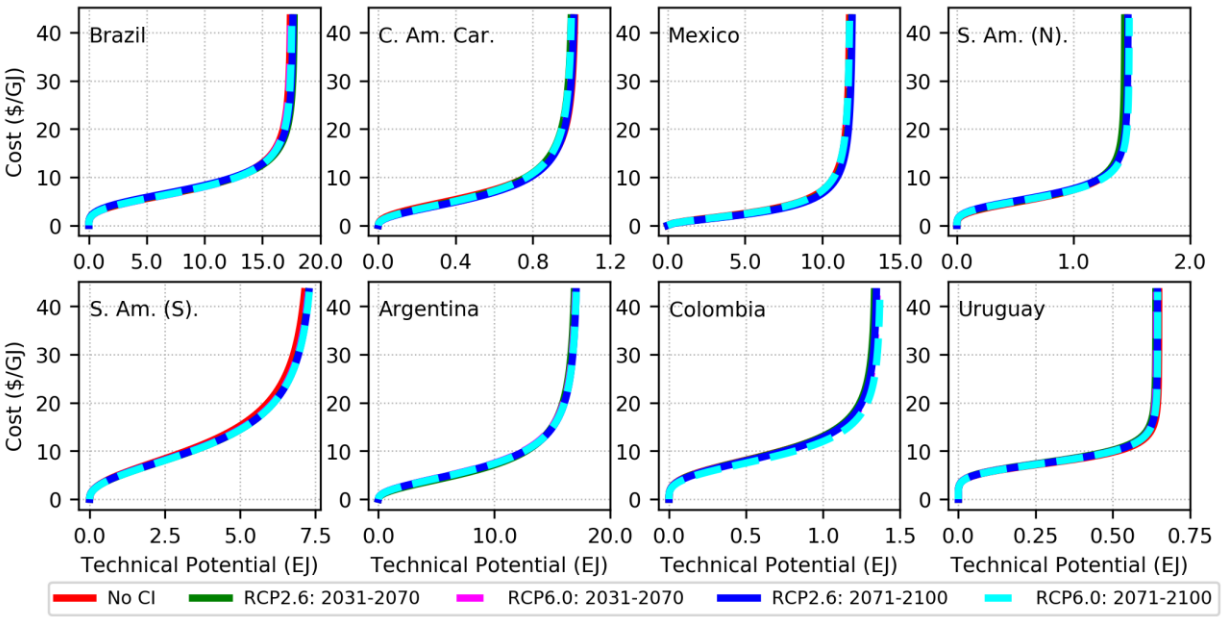


**Supplementary Figure 21.** As in Supplementary Figure 20 but using climate inputs from the HadGEM2-ES model.

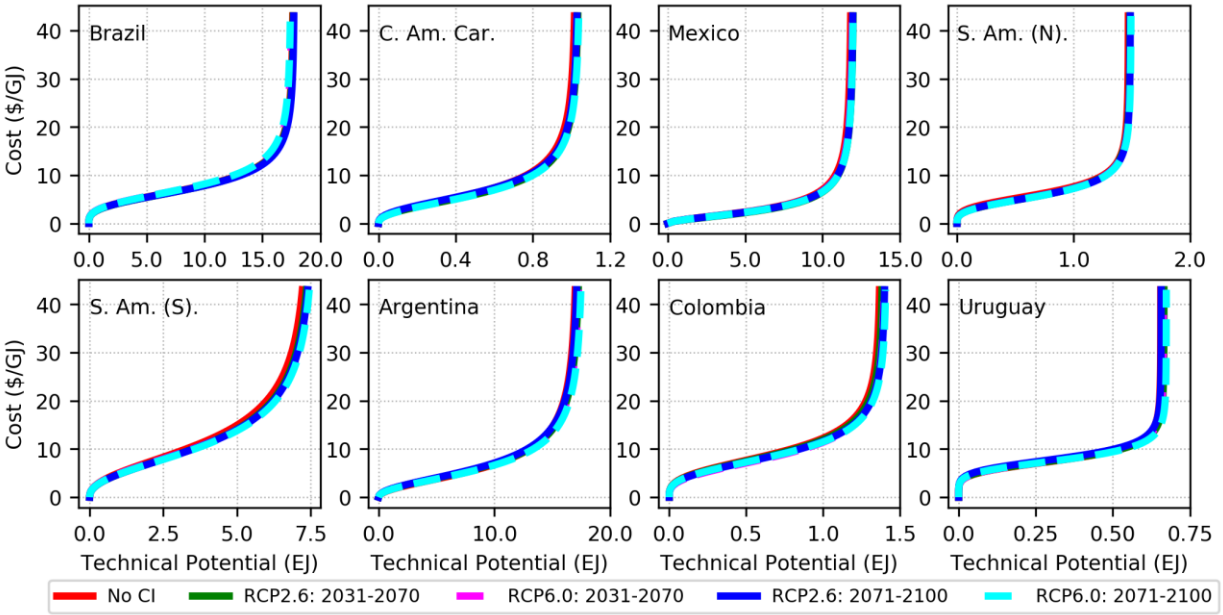


**Supplementary Figure 22.** As in Supplementary Figure 20 but using climate inputs from the IPSL-CM5A-LR model.

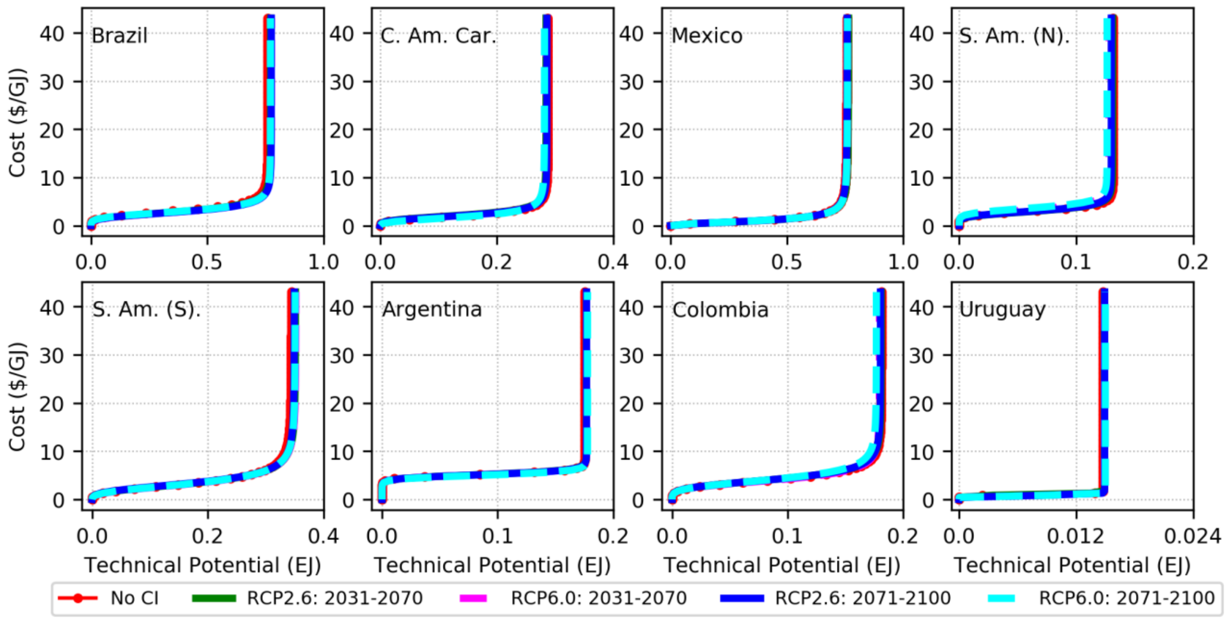


**Supplementary Figure 23.** Latin America and Caribbean cost-supply curves for solar (rooftop PV) energy using climate inputs from the GFDL-ESM2M model under RCPs 2.6 and 6.0. Cost-supply curves for three different periods were implemented in GCAM: two curves representing future climate states and one curve produced from data corresponding to the model historical period (labeled "No CI").

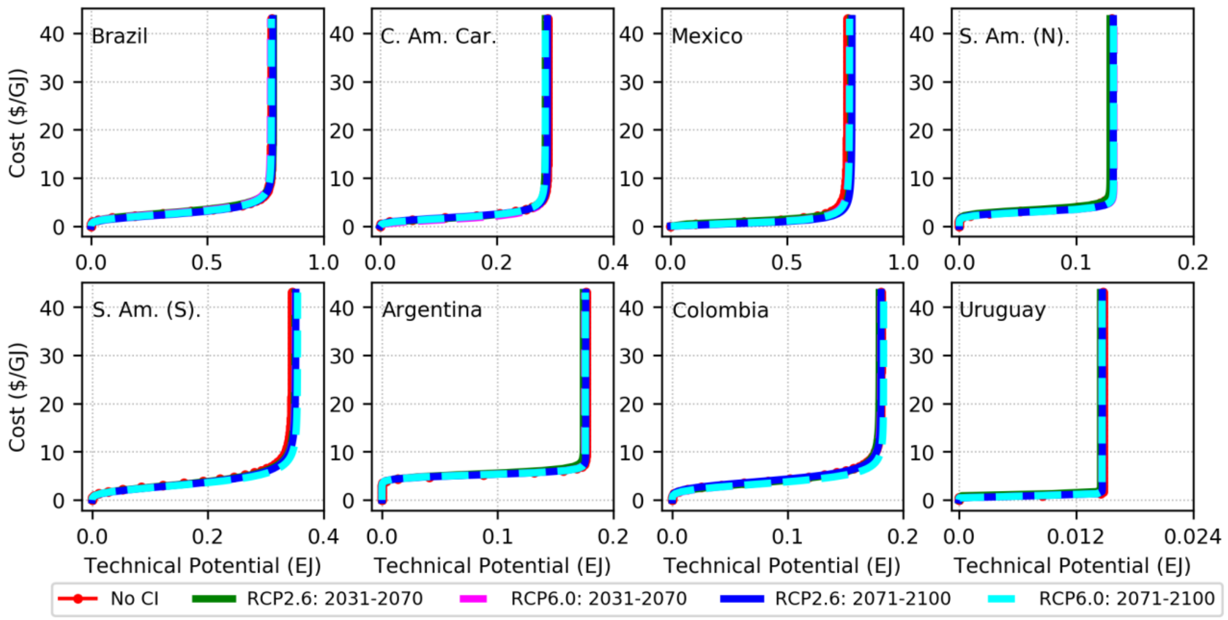


**Supplementary Figure 24.** As in Supplementary Figure 23 but using climate inputs from the HadGEM2-ES model.

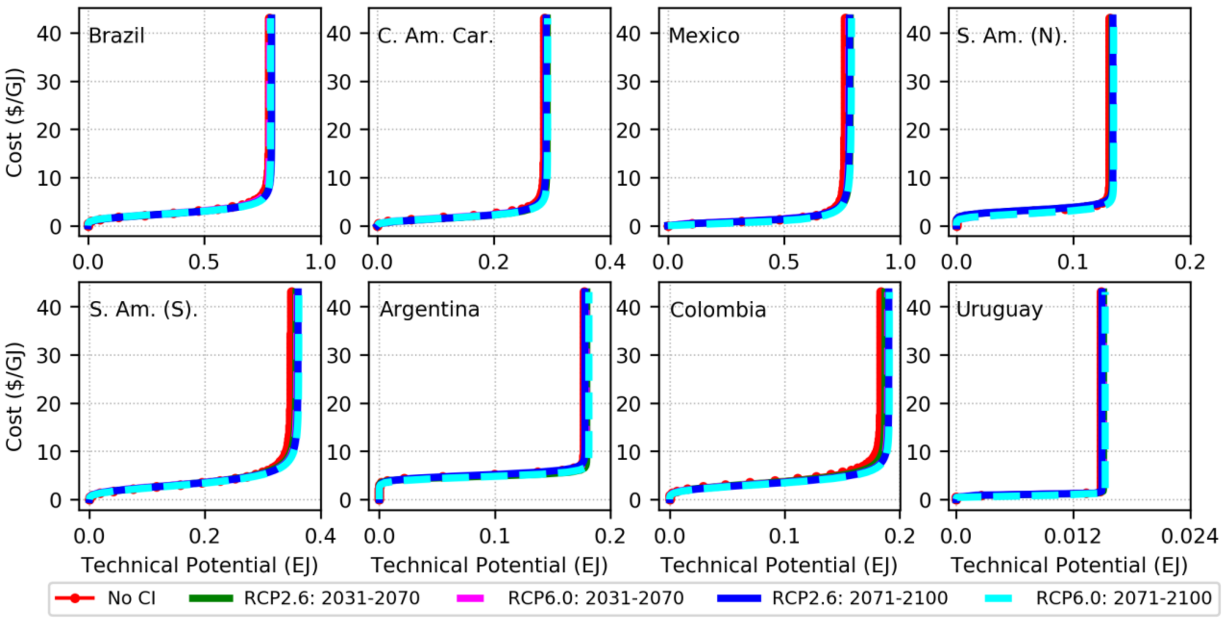


**Supplementary Figure 25.** As in Supplementary Figure 23 but using climate inputs from the IPSL-CM5A-LR model.

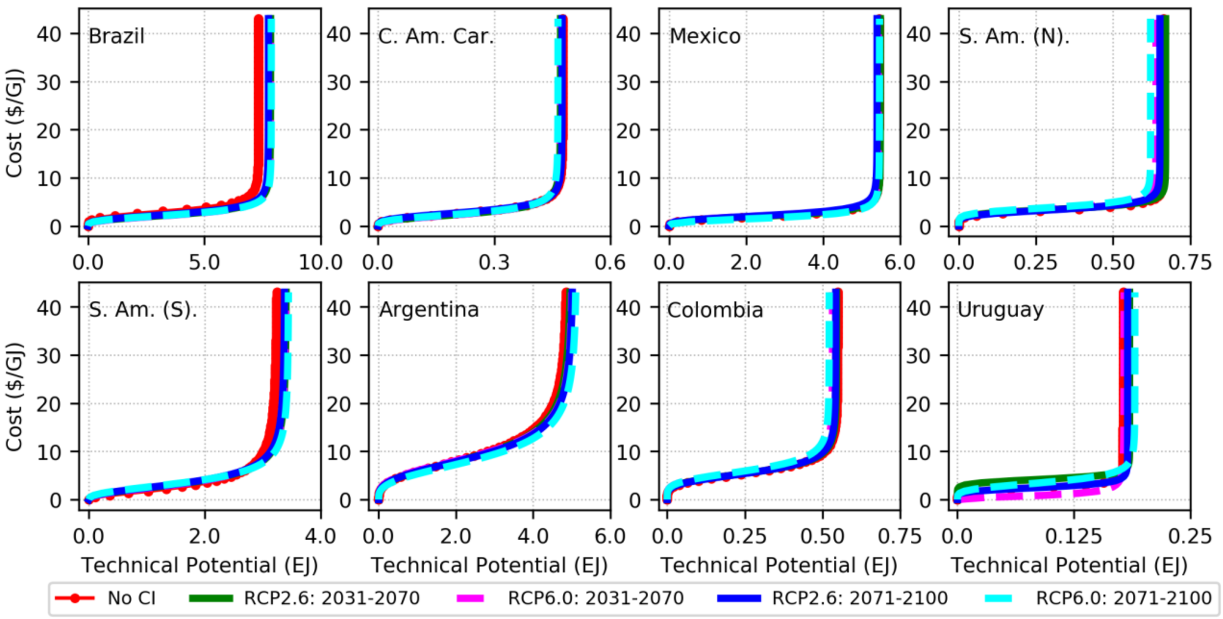


**Supplementary Figure 26.** Latin America and Caribbean cost-supply curves for solar (CSP) energy using climate inputs from the GFDL-ESM2M model under RCPs 2.6 and 6.0. Cost-supply curves for three different periods were implemented in GCAM: two curves representing future climate states and one curve produced from data corresponding to the model historical period (labeled "No CI").

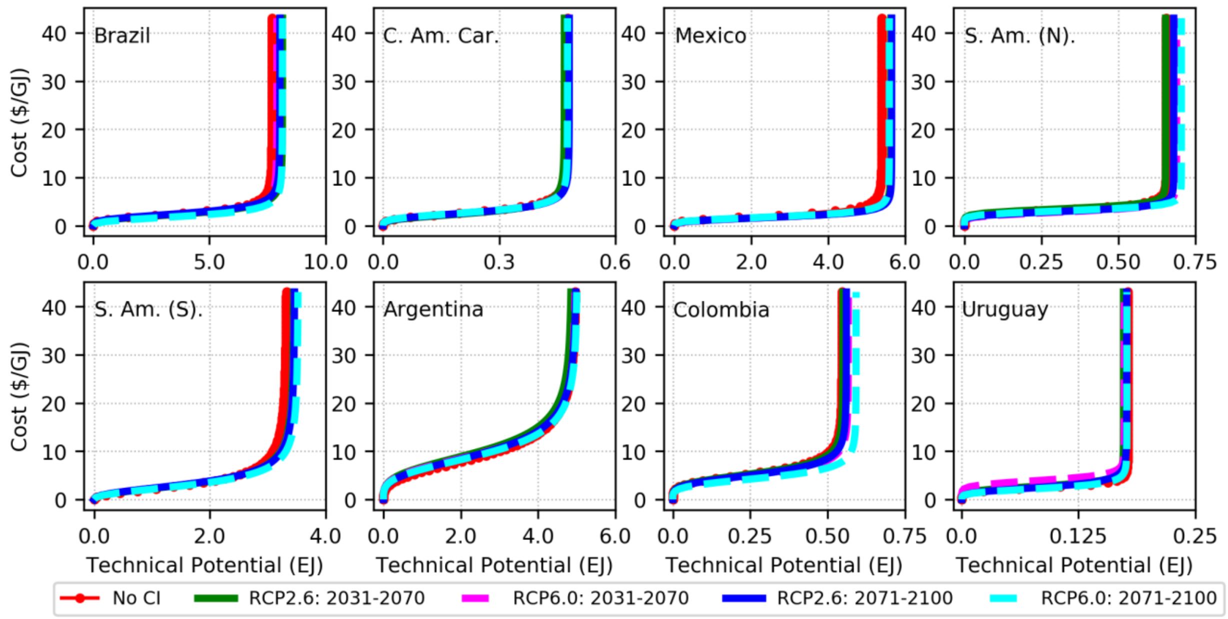


**Supplementary Figure 27.** As in Supplementary Figure 26 but using climate inputs from the HadGEM2-ES model.

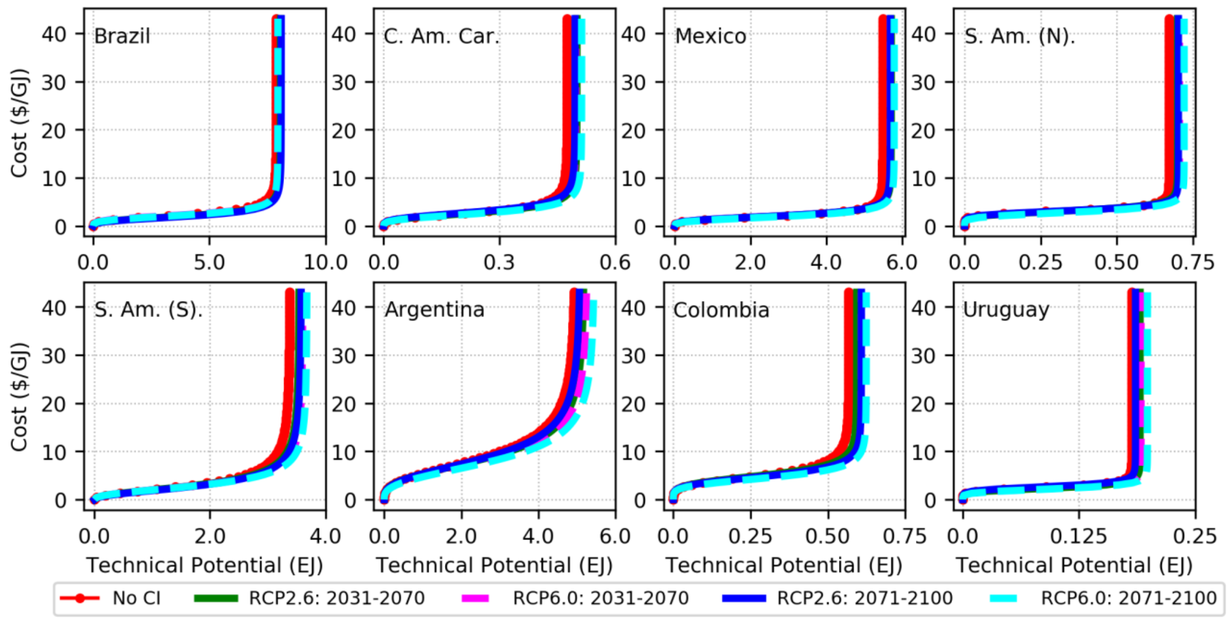


**Supplementary Figure 28.** As in Supplementary Figure 26 but using climate inputs from the IPSL-CM5A-LR model.

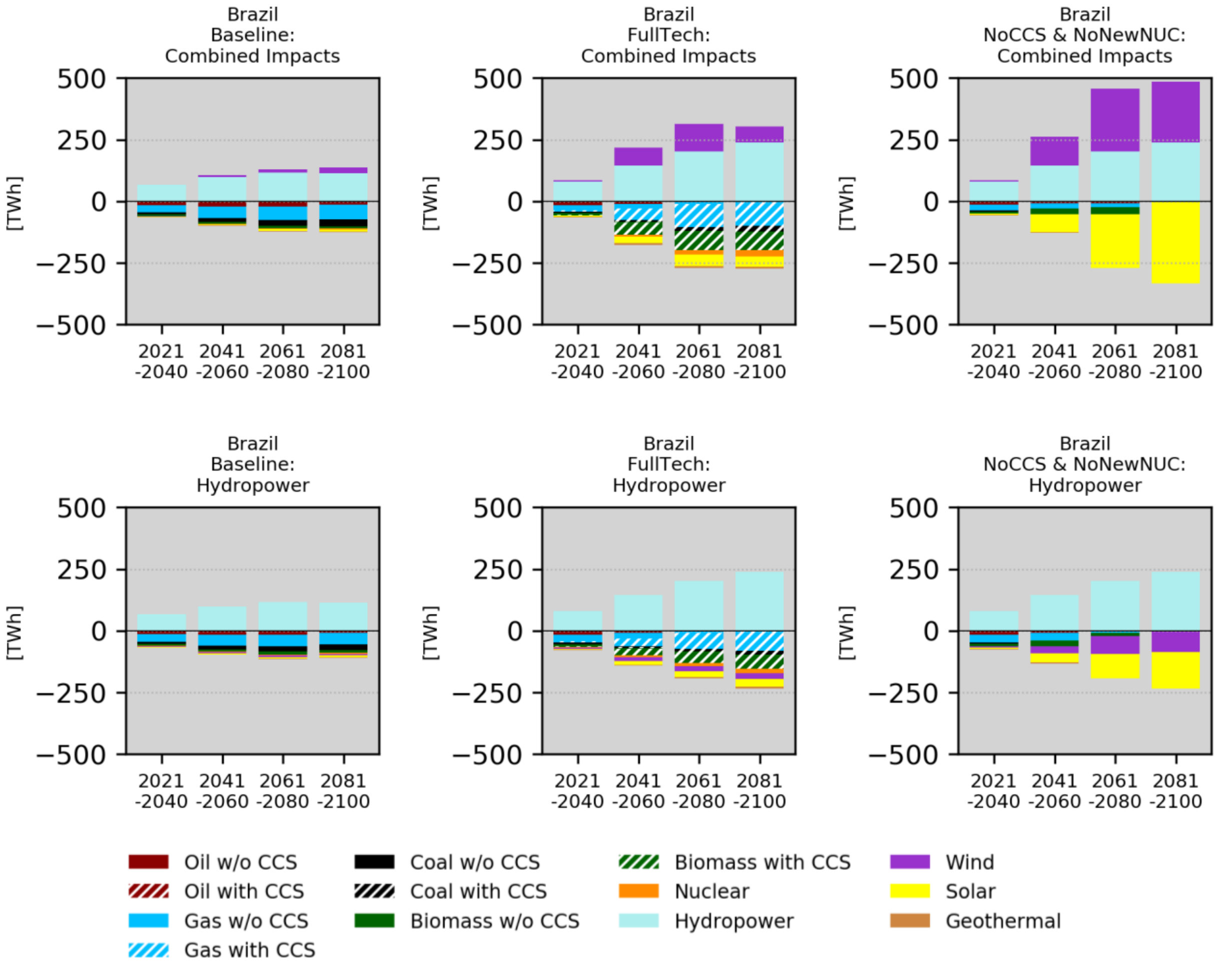


**Supplementary Figure 29.** Model mean differences in electricity production by technology in Brazil assuming climate change impacts on renewables for all climate-impact scenarios explored in this study. Differences are calculated by technology using cumulative generation changes by distinct periods (2021-2040, 2041-2060, 2061-2080, 2081-2100) and are relative to the corresponding *No-climate impacts* scenarios.

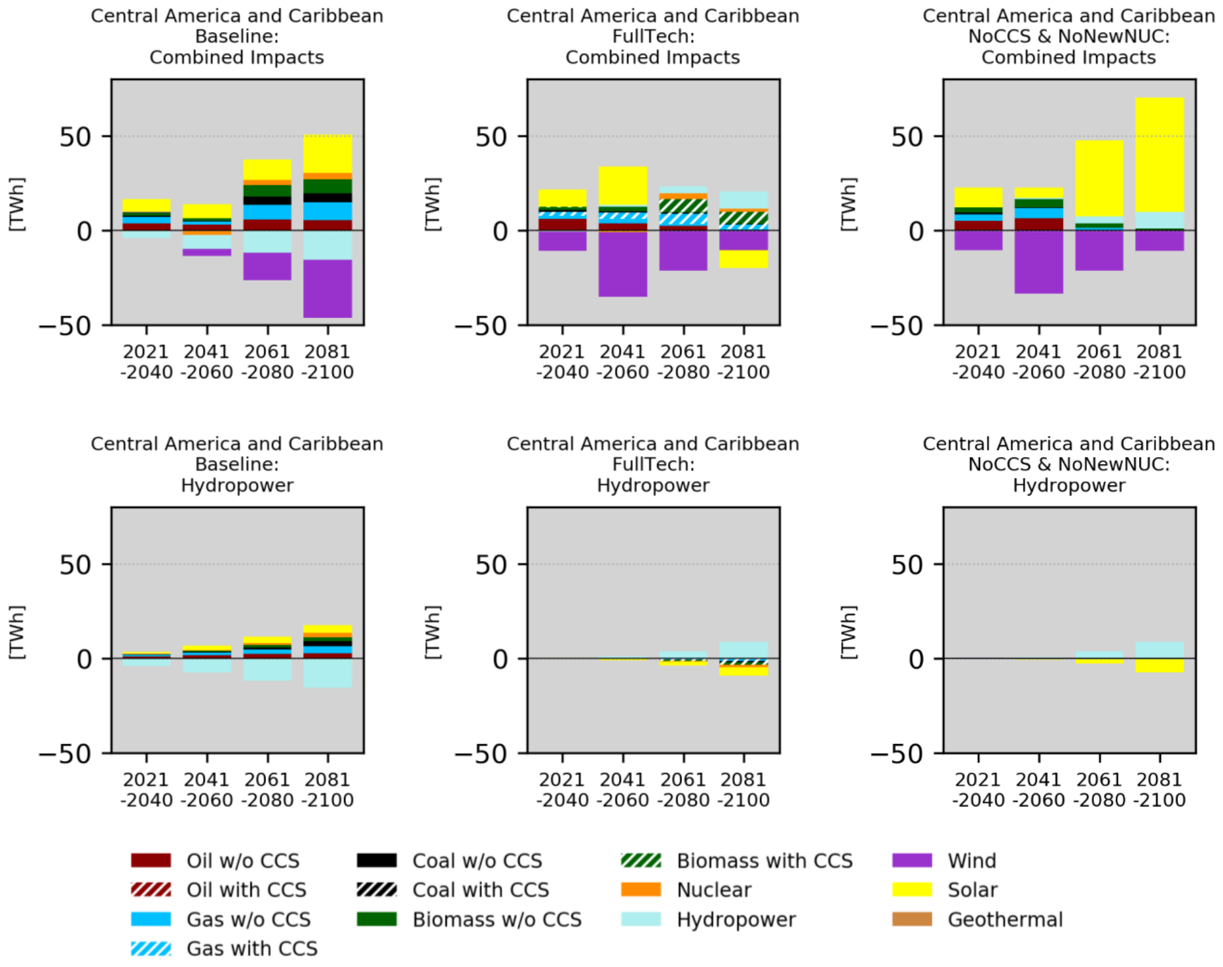


**Supplementary Figure 30.** As in Supplementary Figure 29 but for Central America and Caribbean.

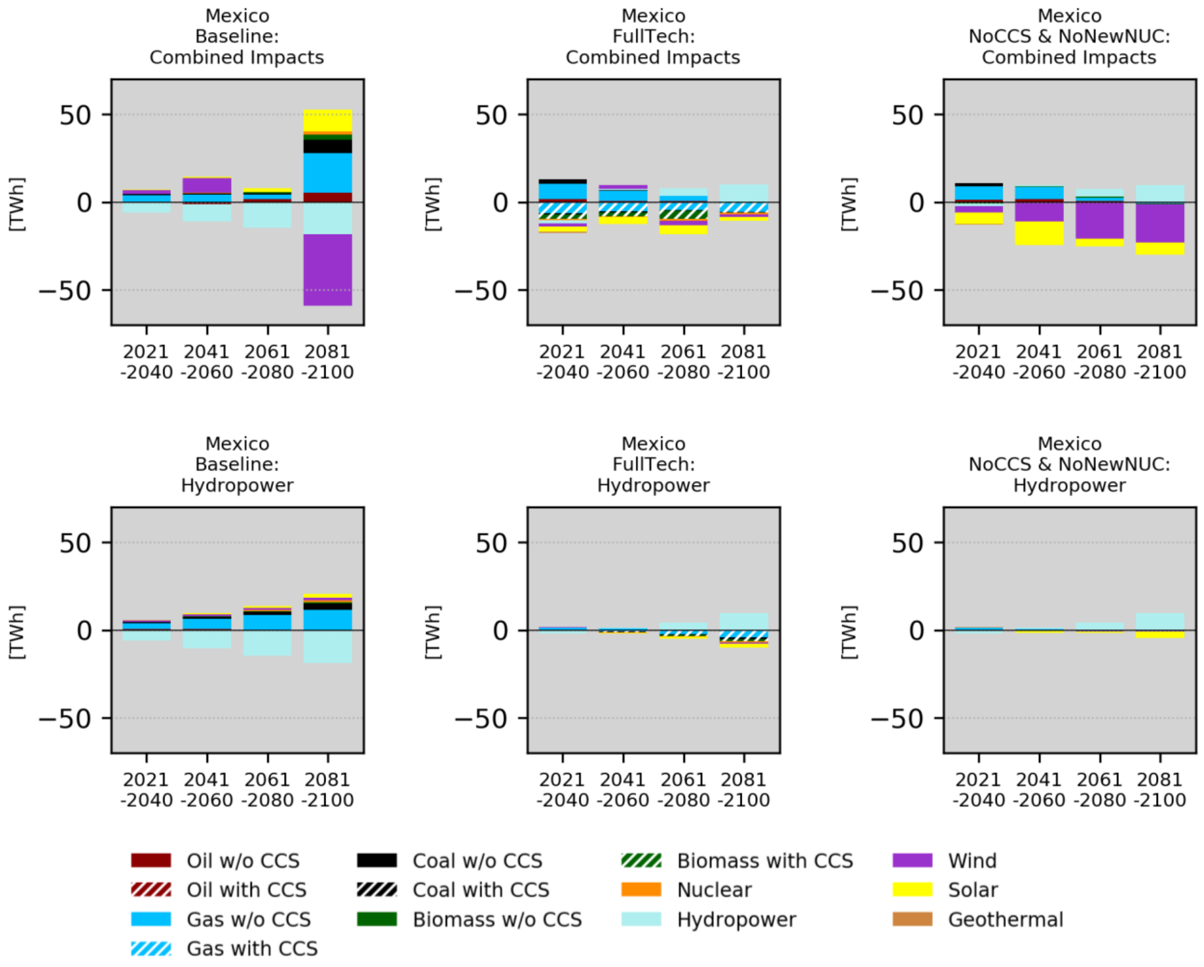


**Supplementary Figure 31.** As in Supplementary Figure 29 but for Mexico.

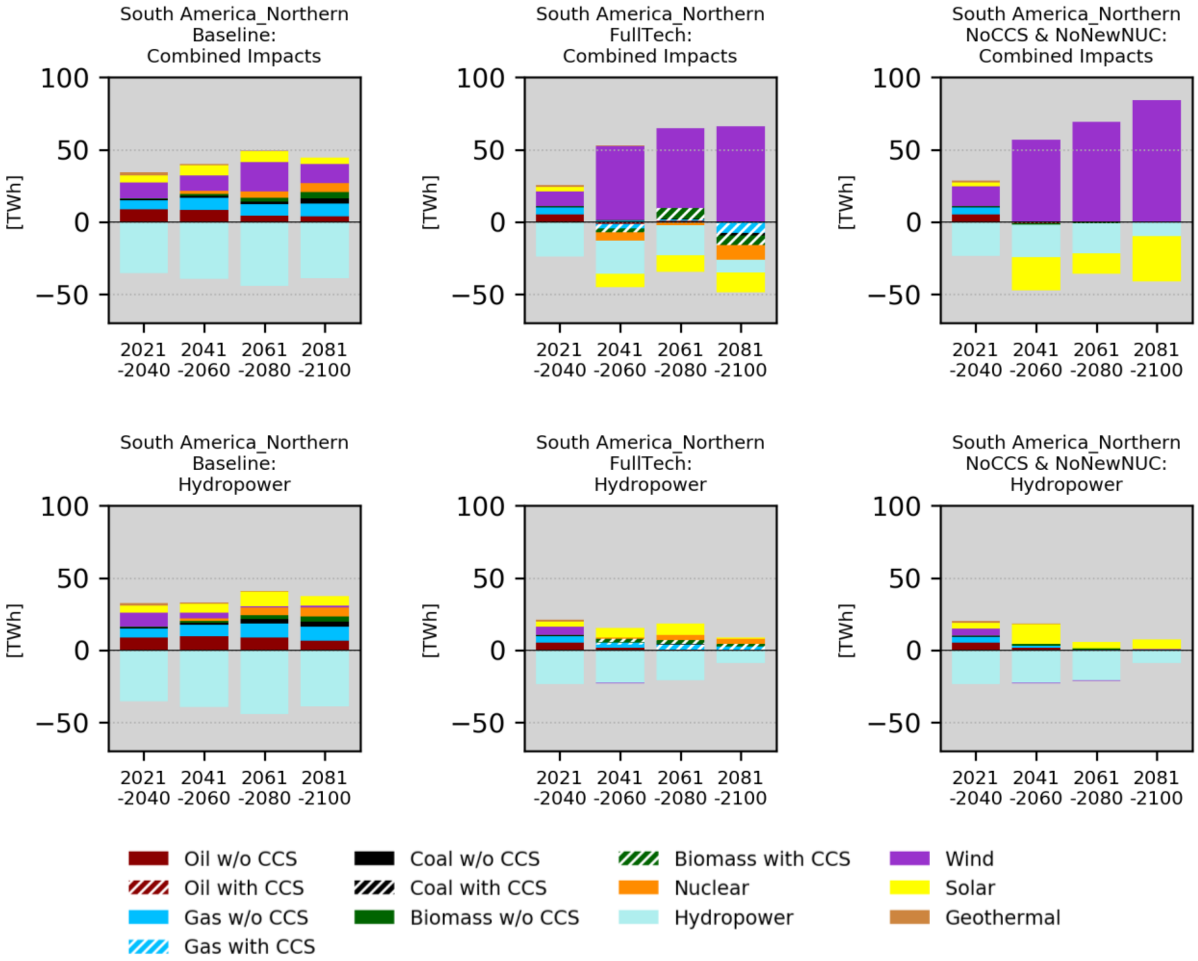


**Supplementary Figure 32.** As in Supplementary Figure 29 but for South America Northern.

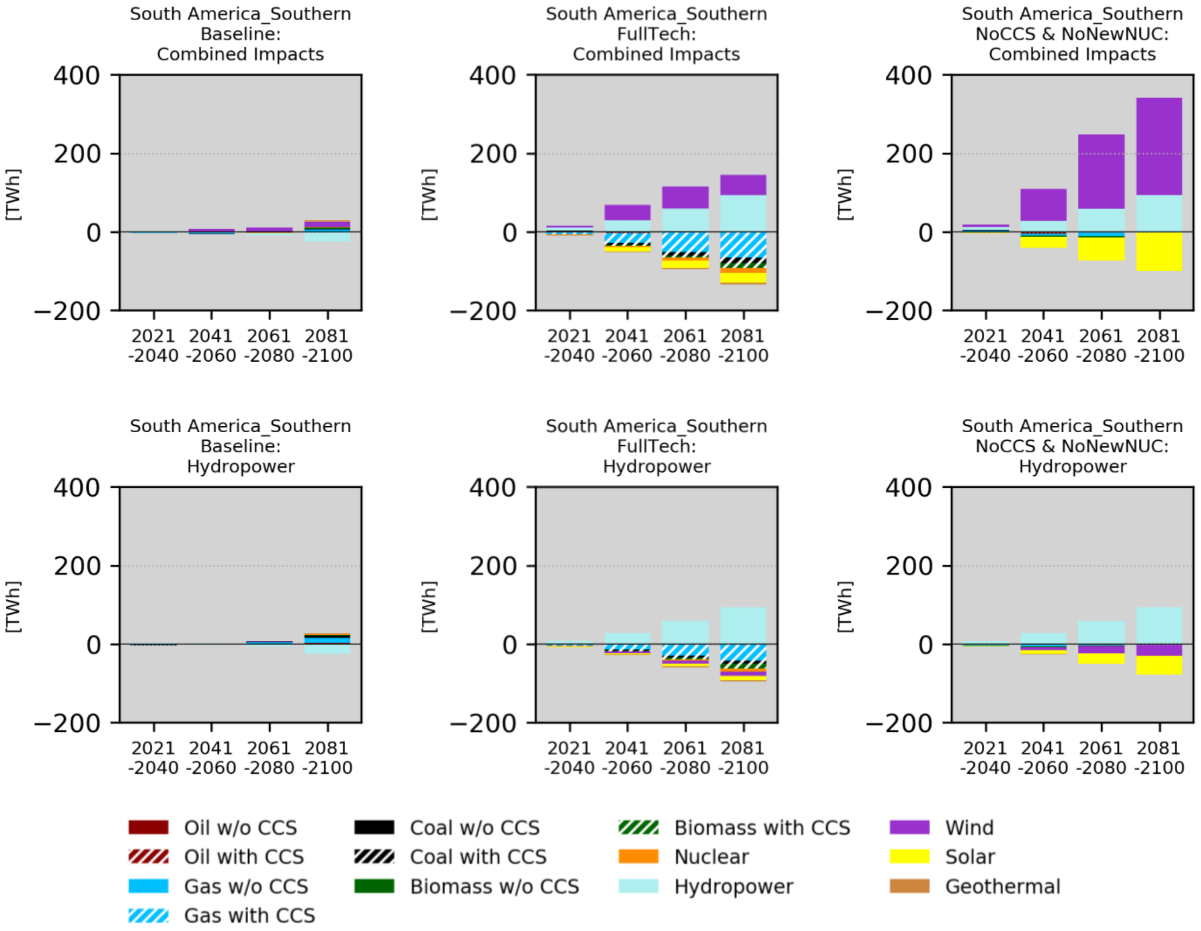


**Supplementary Figure 33.** As in Supplementary Figure 29 but for South America Southern.

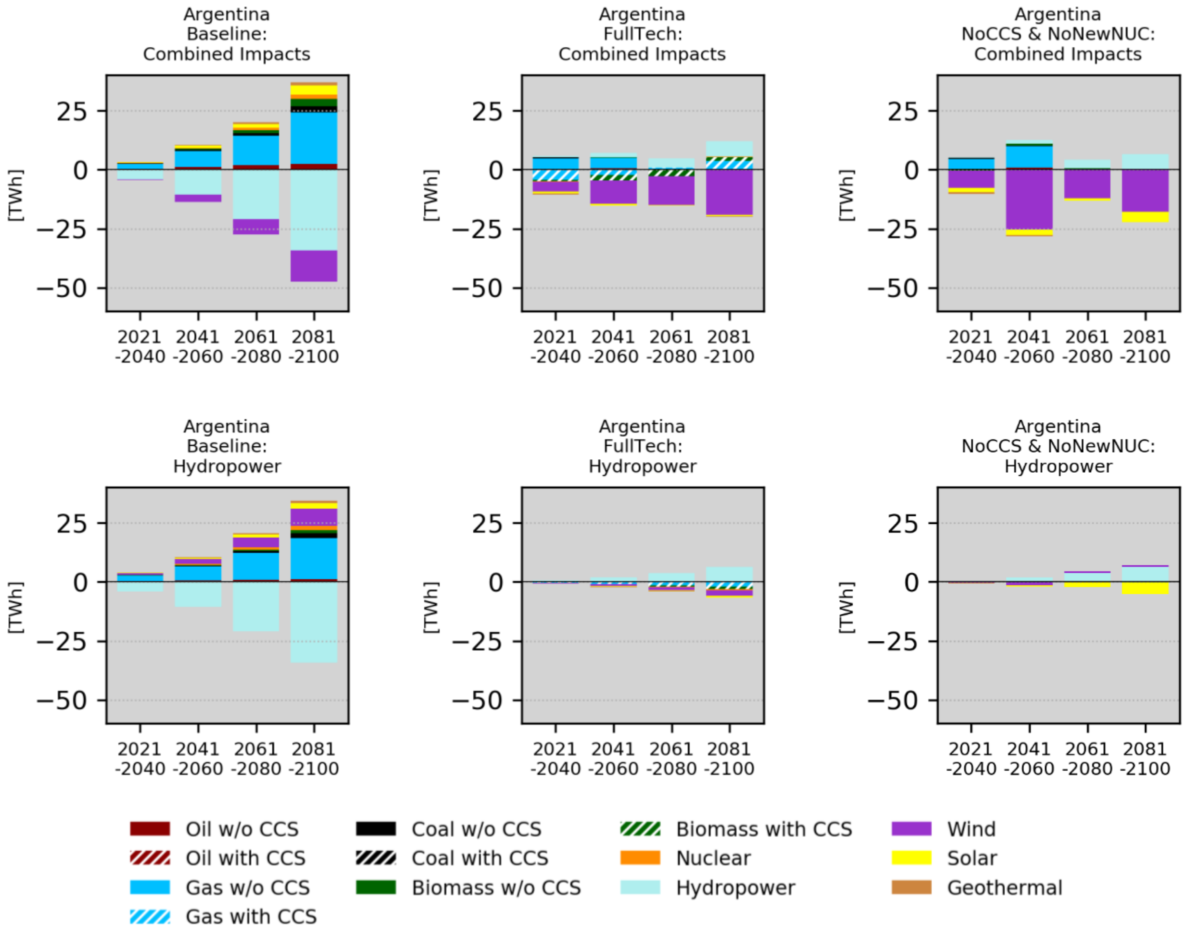


**Supplementary Figure 34.** As in Supplementary Figure 29 but for Argentina.

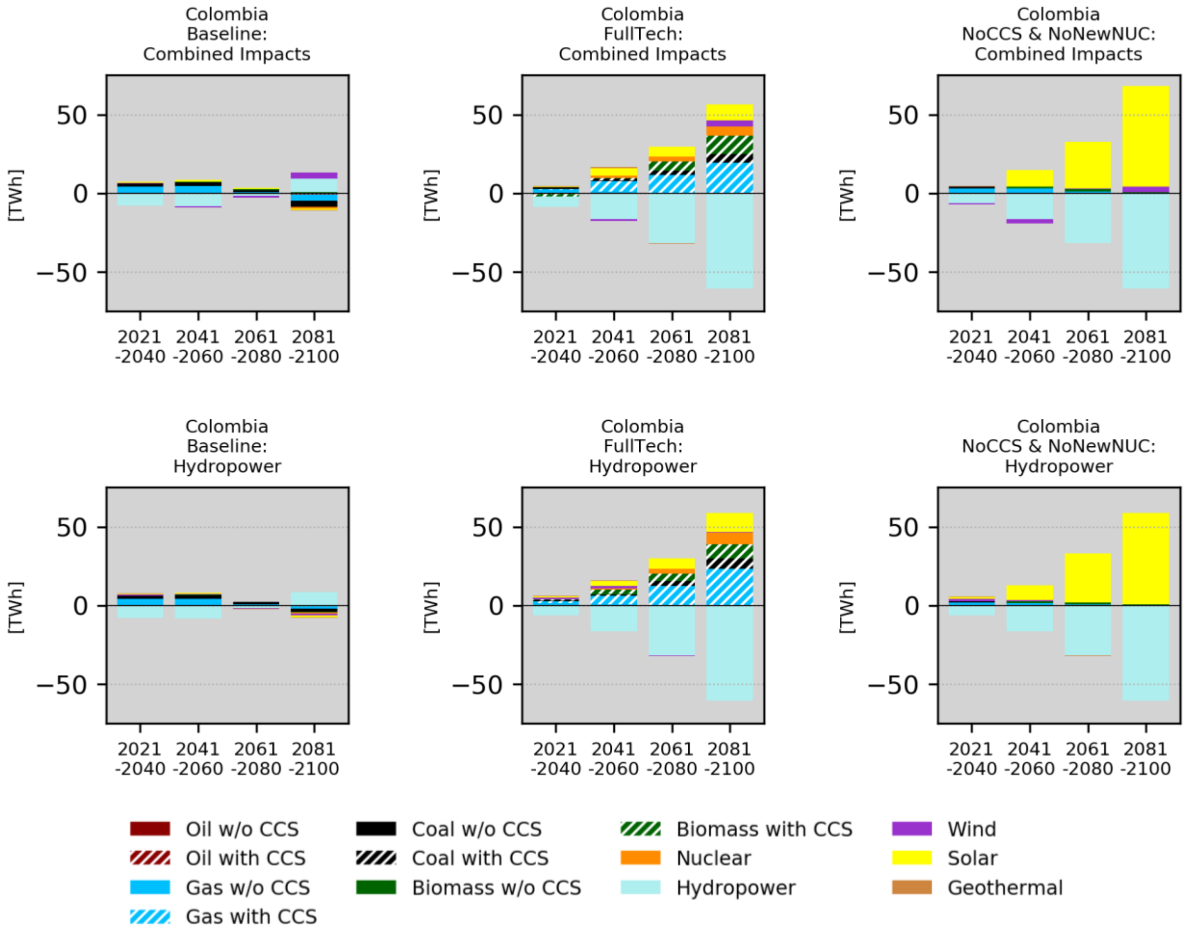


**Supplementary Figure 35.** As in Supplementary Figure 29 but for Colombia.

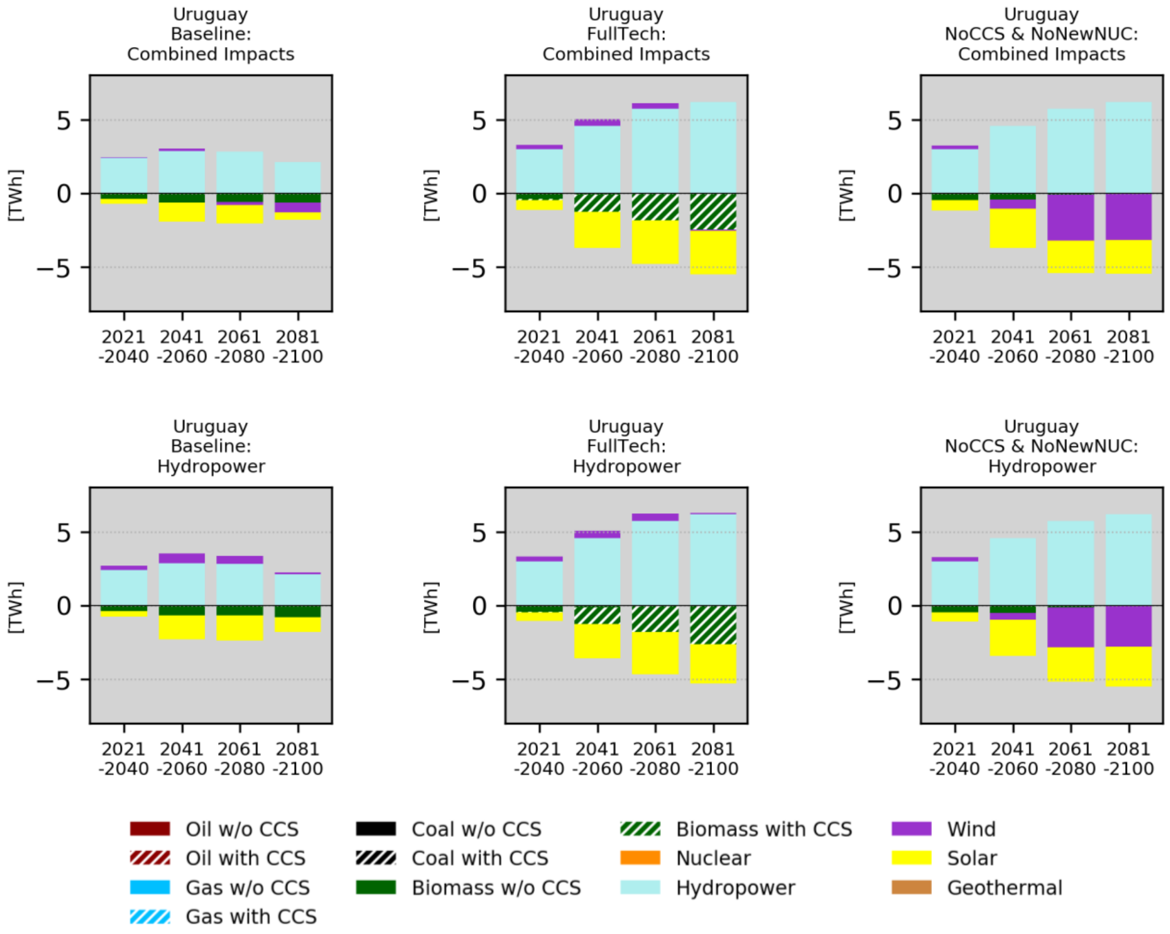


**Supplementary Figure 36.** As in Supplementary Figure 29 but for Uruguay.

Supplementary Figures 37–44 compare the annual rates of capacity additions projected by scenarios in this study with historical rates. Despite the distinct variation across GCAM-LAC regions, in general, the projected rates of capacity additions tend to be considerably larger than the historical averages, particularly for the *NoCCS & NoNewNUC* scenario. The larger rates of capacity additions in the GCAM scenarios relate to growing demands (exacerbated by larger end-use electrification in the mitigation scenarios) as well as the higher capacity requirements of intermittent renewables (i.e., intermittent renewables with lower capacity factors that require more capacity per unit of electricity generated compared with other technologies).

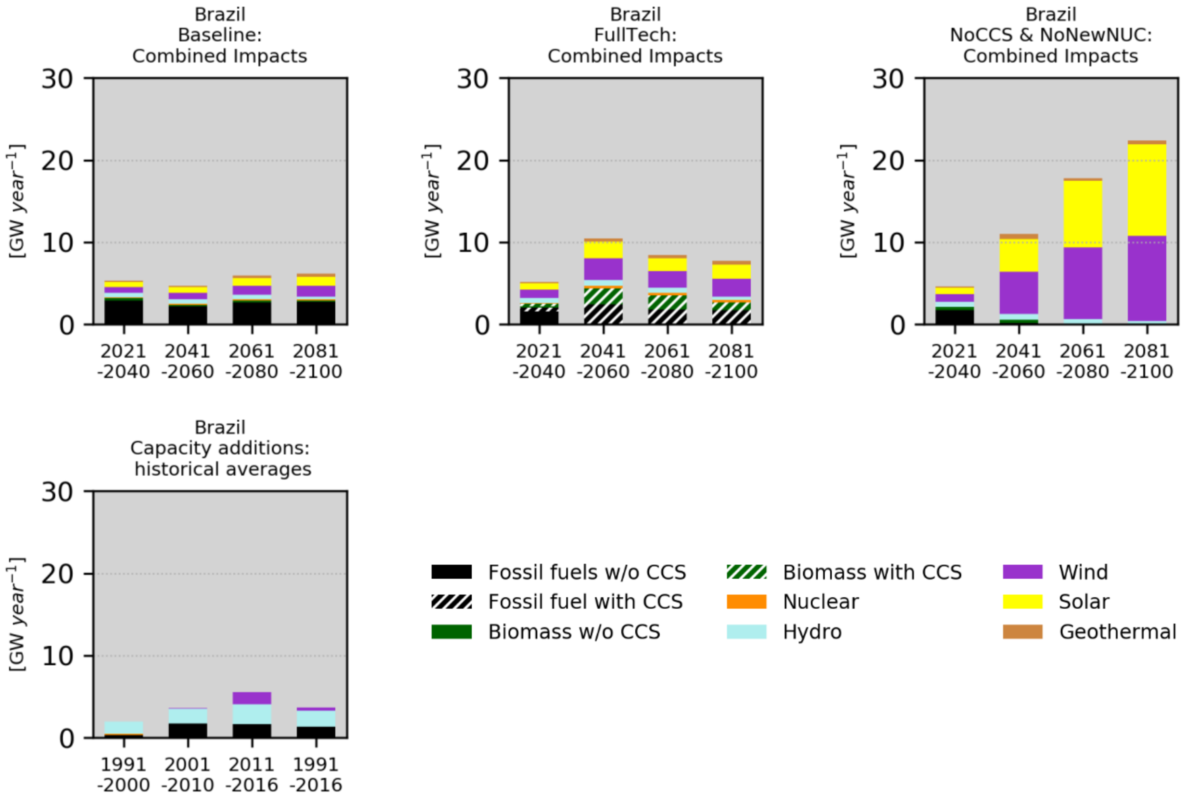


**Supplementary Figure 37.** Capacity additions by generating capacity in the electric power sector in Brazil assuming climate change impacts on all renewables. Historical averages are based on data from the Energy Statistics Database of the United Nations Statistics Division (available at http://data.un.org/Explorer.aspx). Note that data from the Energy Statistics Database: 1) do not disaggregate fossil fuels by distinct sources; 2) do not include biomass.

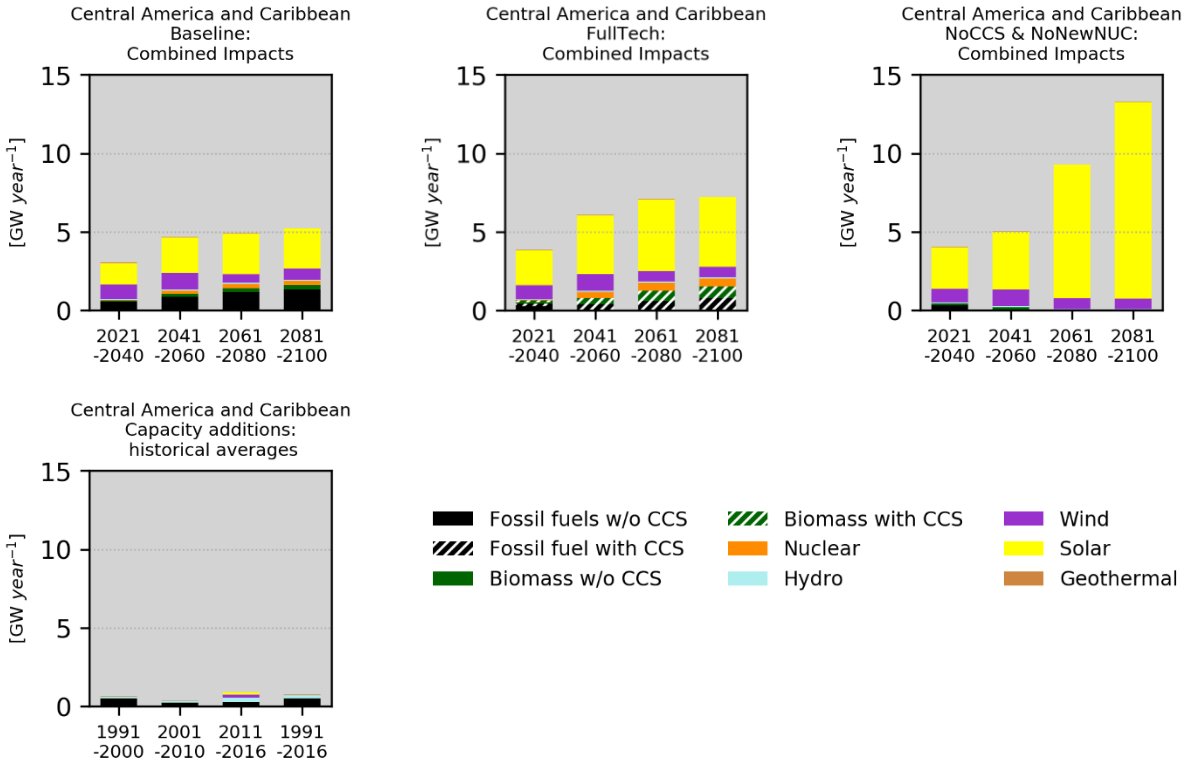


**Supplementary Figure 38.** As in Supplementary Figure 37 but for Central America and Caribbean.

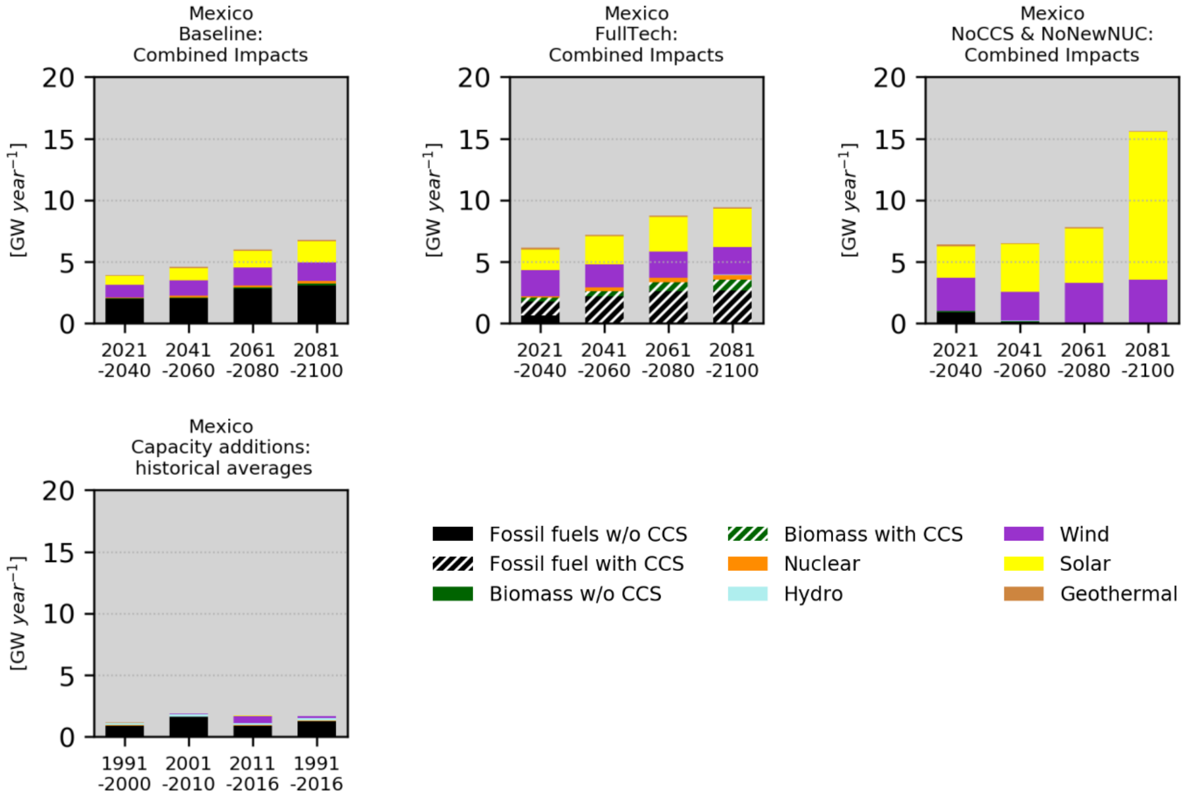


**Supplementary Figure 39.** As in Supplementary Figure 37 but for Mexico.

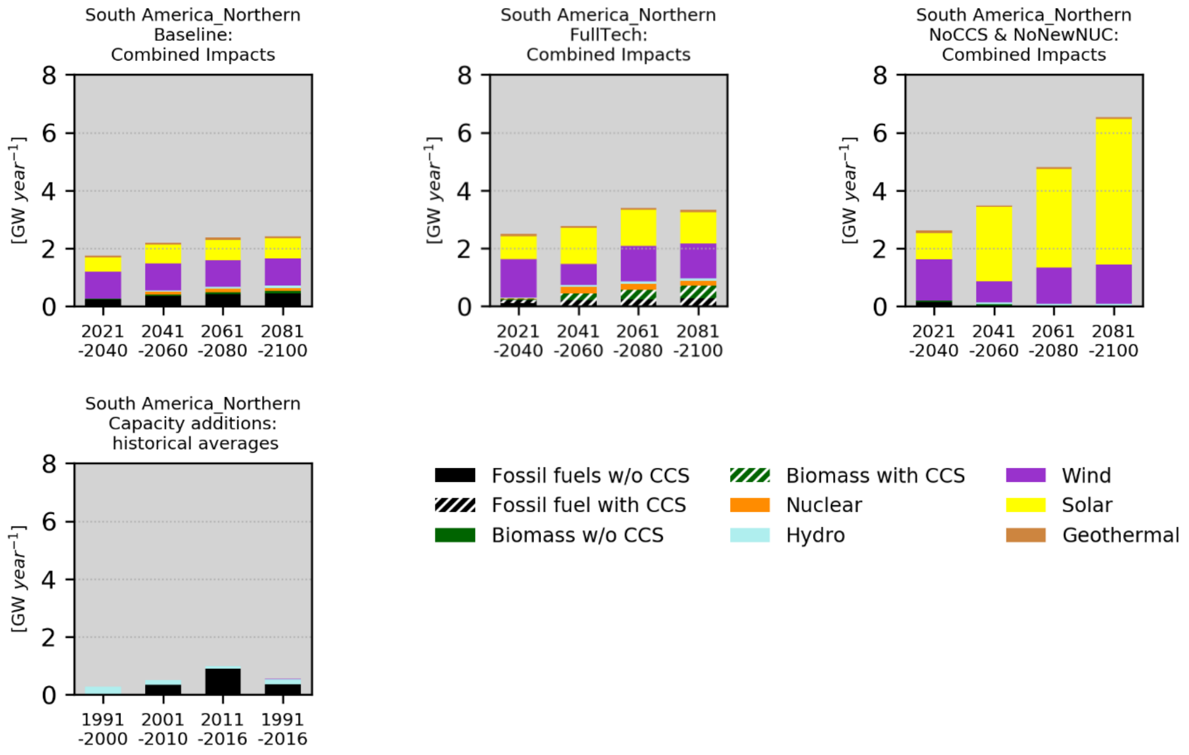


**Supplementary Figure 40.** As in Supplementary Figure 37 but for South America Northern.

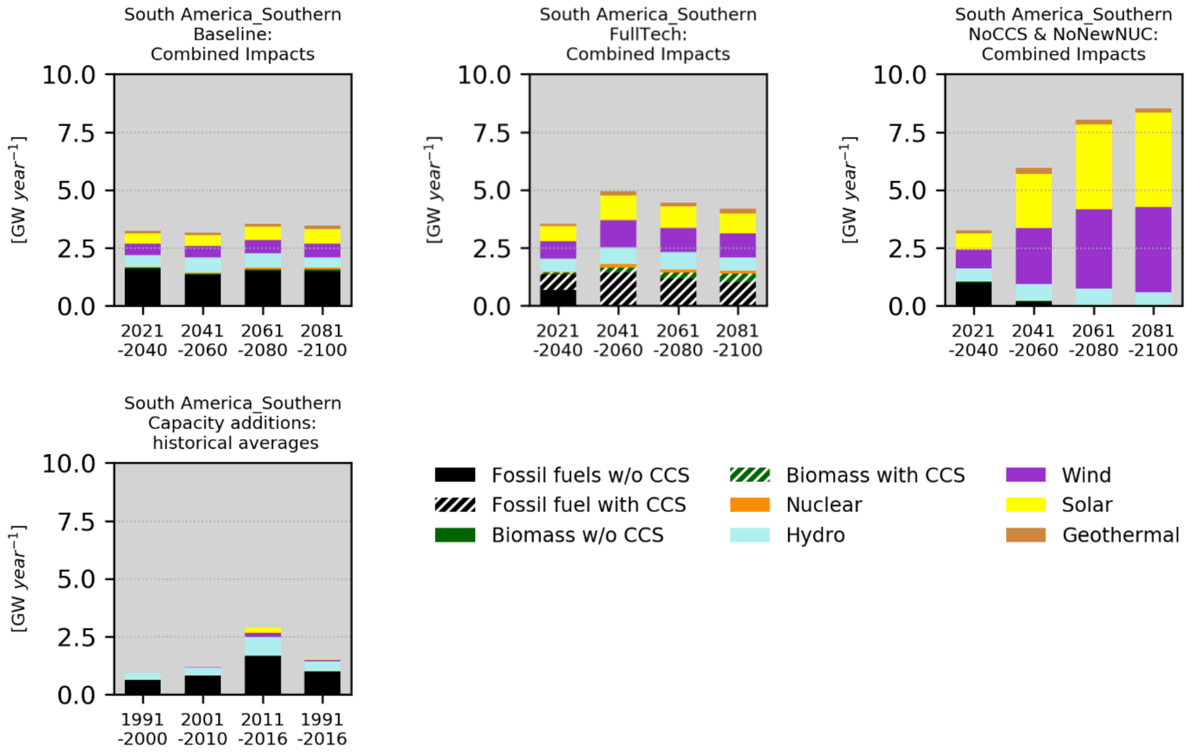


**Supplementary Figure 41.** As in Supplementary Figure 37 but for South America Southern.

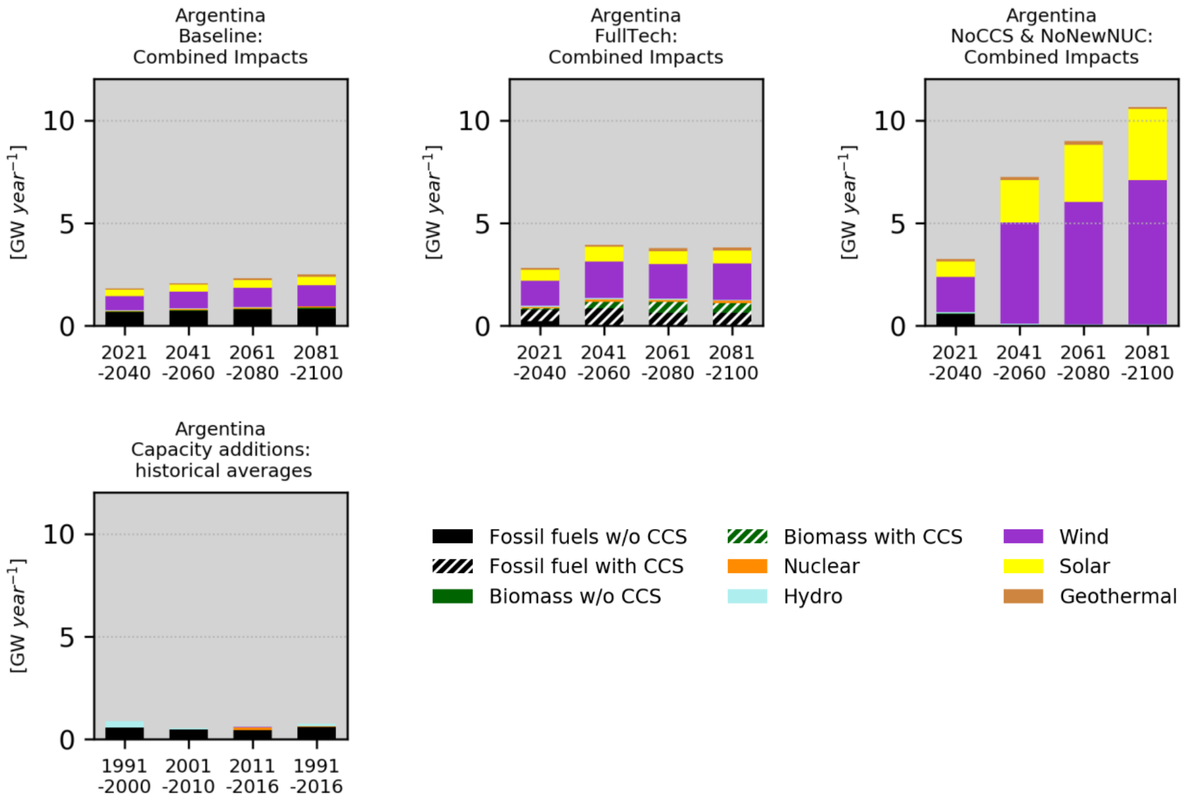


**Supplementary Figure 42.** As in Supplementary Figure 37 but for Argentina.

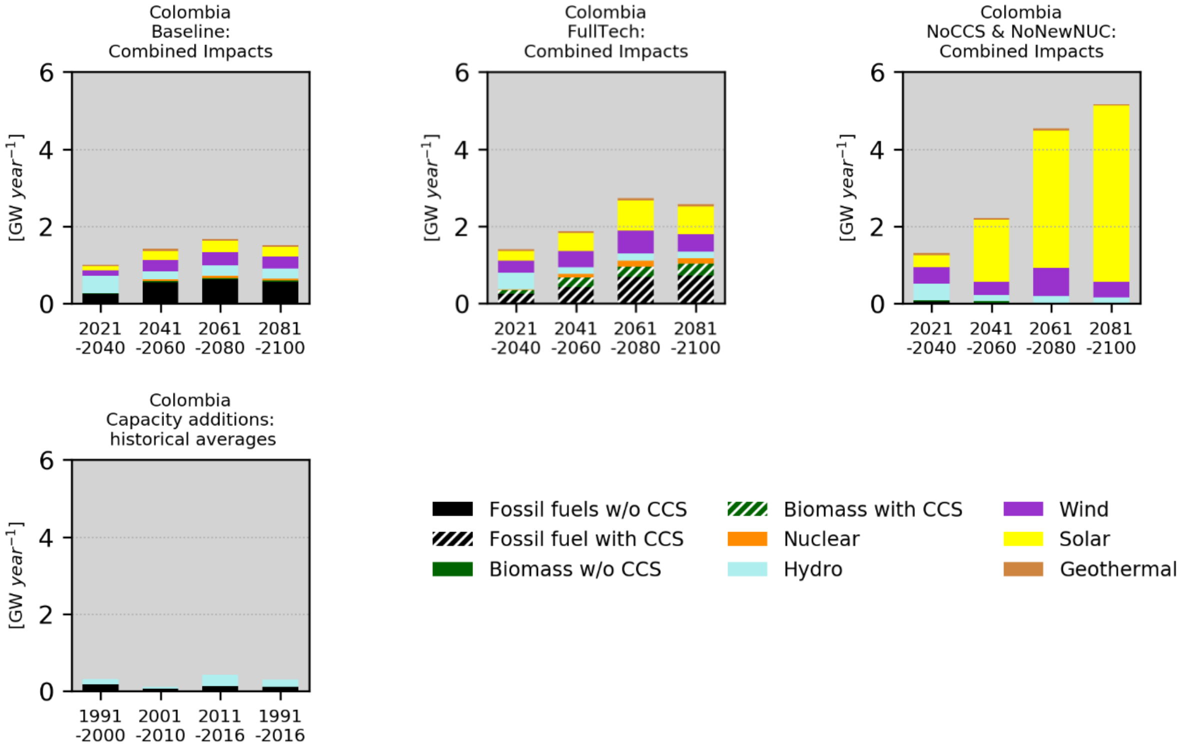


**Supplementary Figure 43.** As in Supplementary Figure 37 but for Colombia.

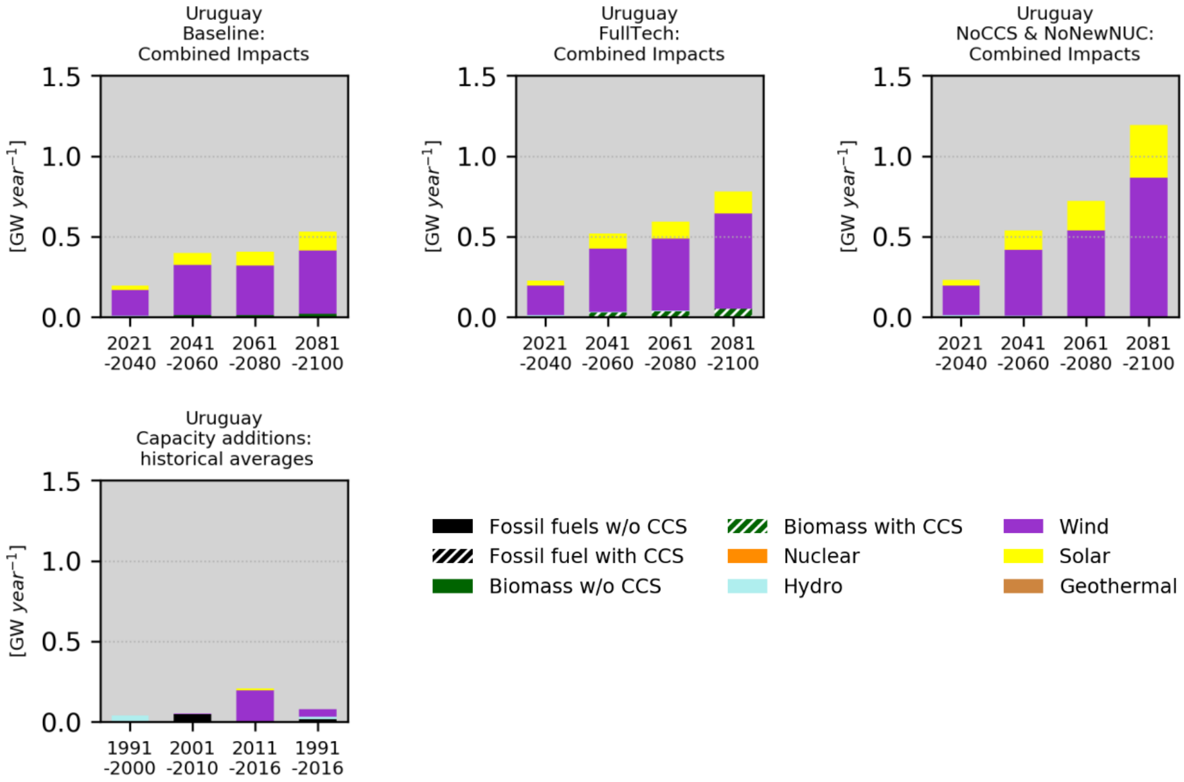


**Supplementary Figure 44.** As in Supplementary Figure 37 but for Uruguay.

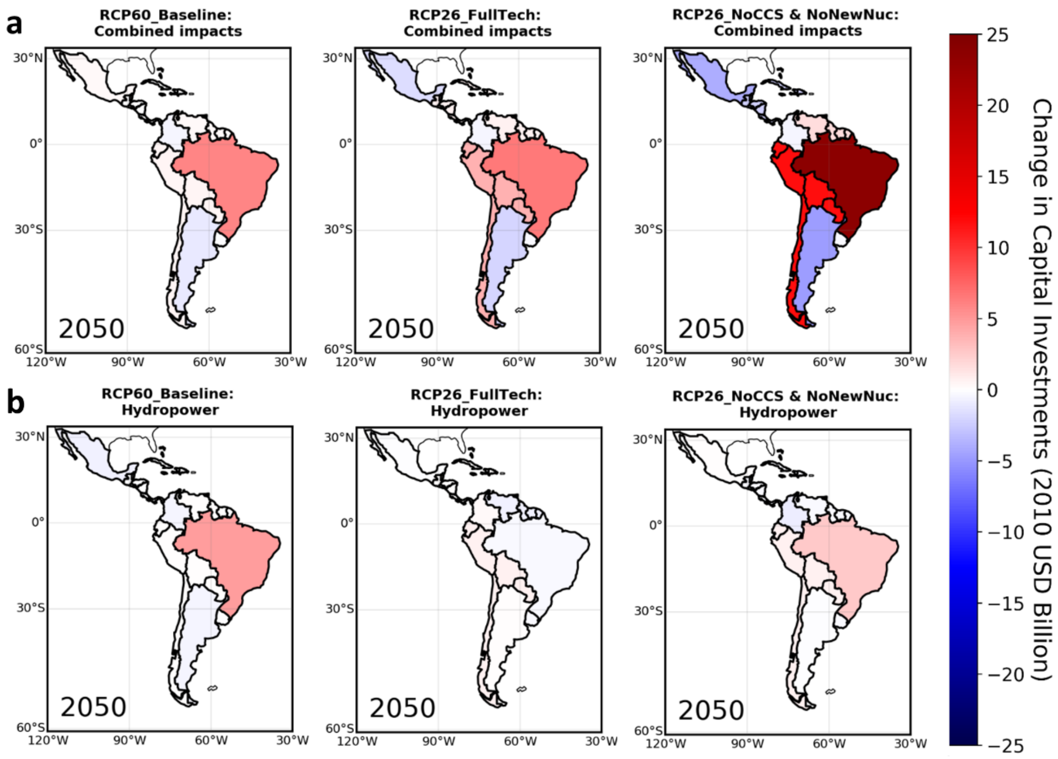


**Supplementary Figure 45.** Model mean changes in total capital investment requirements in LAC by scenario under distinct assumptions on climate change impacts on renewables. Absolute differences computed under the *Combined impacts* scenarios (**a**) and *Hydropower* scenarios (**b**). Changes are calculated using cumulative capital costs (United States dollar – USD) in the 2020 – 2050 period and are relative to the *No-climate impacts* simulation (i.e., positive values mean that scenarios with climate impacts on renewables show increased costs). Full range of estimated costs: USD -5 to +24 billion.

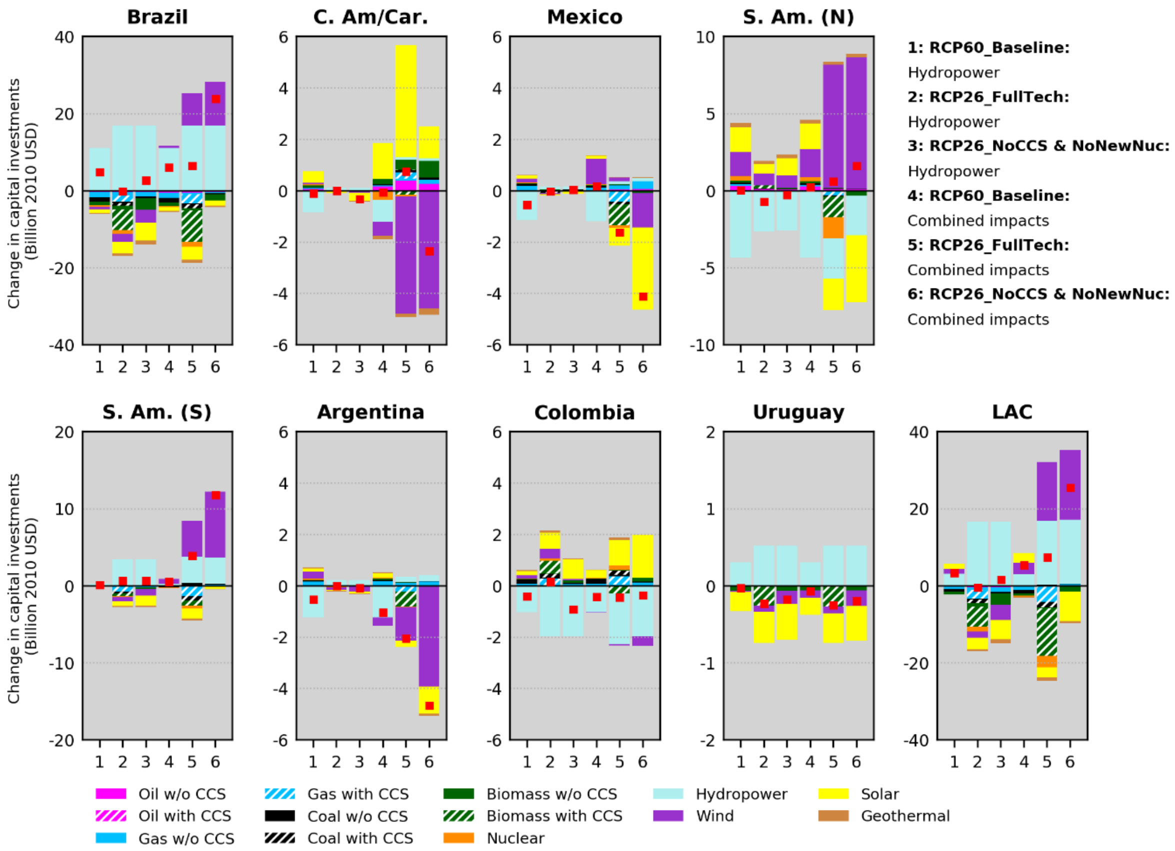


**Supplementary Figure 46.** Model mean differences in capital investments by technology in LAC assuming climate change impacts on renewables. Differences are calculated by technology using cumulative investments (USD) in the 2020 – 2050 period. Differences are relative to the *No-climate impacts* scenarios (i.e., positive values mean that scenarios with climate impacts show increased costs). The red squares indicate the net of the positive and negative changes for a given scenario (and are equal to the total investment changes plotted in Supplementary Fig. 45). Note the different y axis scales across regions, and that the y axis scales do not match those presented in Fig. 3 of the main text.

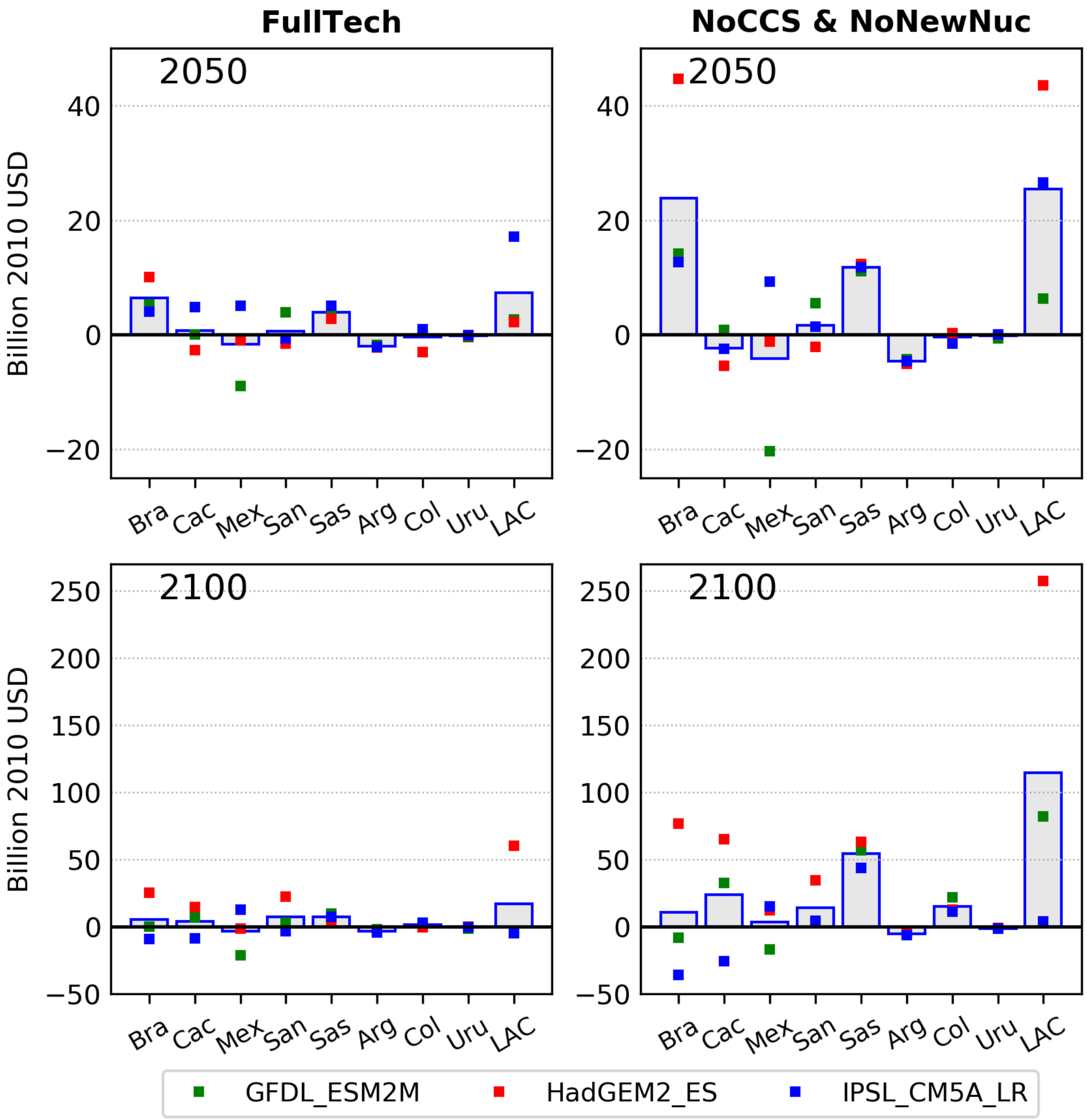


**Supplementary Figure 47.** Differences in total capital investments in LAC per technology scenario and GCM assuming climate change impacts on all renewables. Changes are calculated using cumulative capital investments in the 2020 – 2050 (top) and 2020 – 2100 (bottom) periods. Changes are relative to the *No-climate impacts* simulations (i.e., positive values mean that scenarios with climate impacts on renewables show incremental costs). GCAM LAC regions covered: Brazil (Bra), Central America and the Caribbean (Cac), Mexico (Mex), South America_Northern (San), South America_Southern (Sas), Argentina (Arg), Colombia (Col) and Uruguay (Uru).

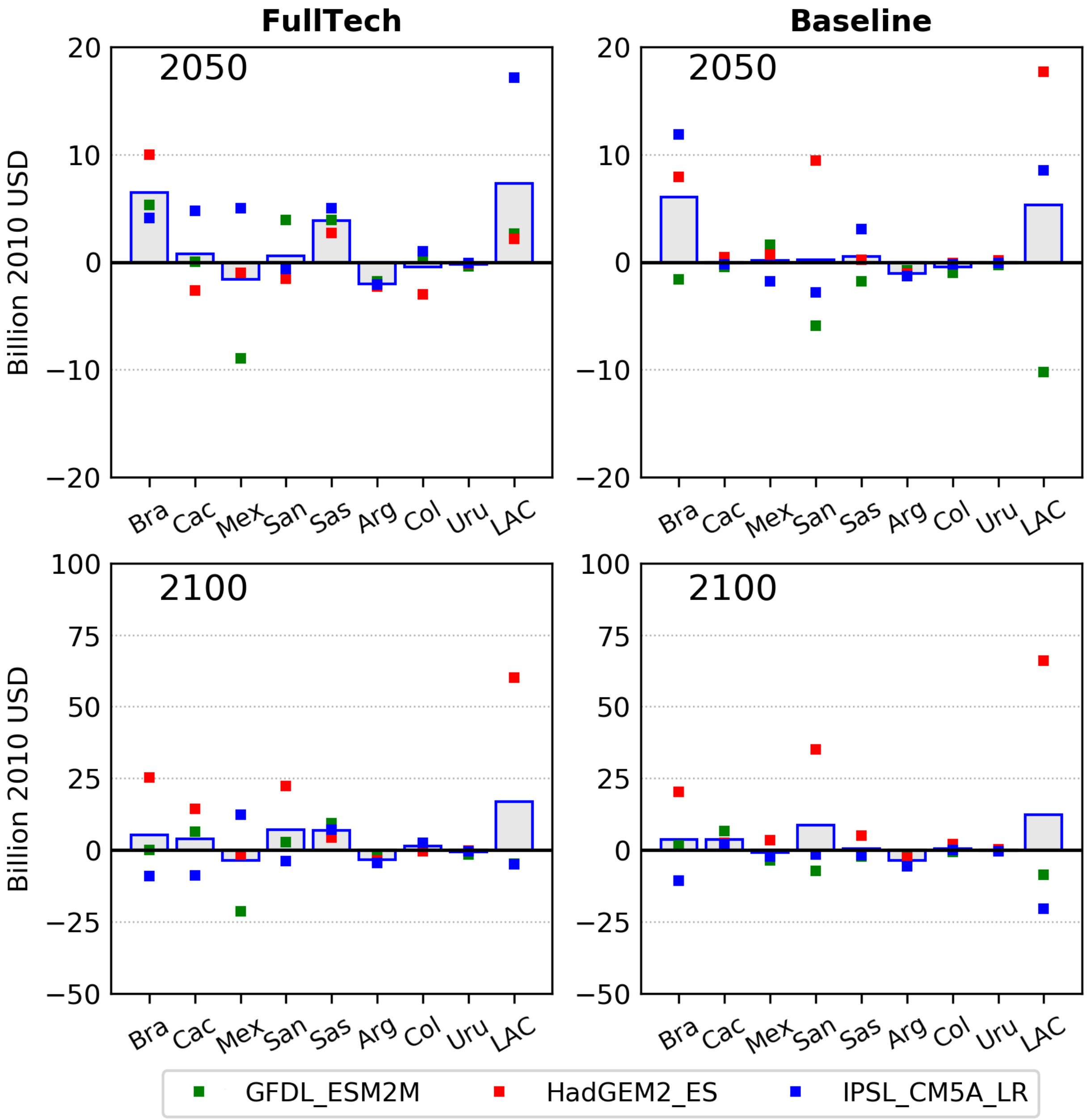


**Supplementary Figure 48.** As in Supplementary Figure 47 but comparing the *RCP26_FullTech: Combined impacts* and the *RCP60_Baseline: Combined impacts* scenarios. To improve visibility, the y axis scales do not match those presented in Supplementary Figure 47.

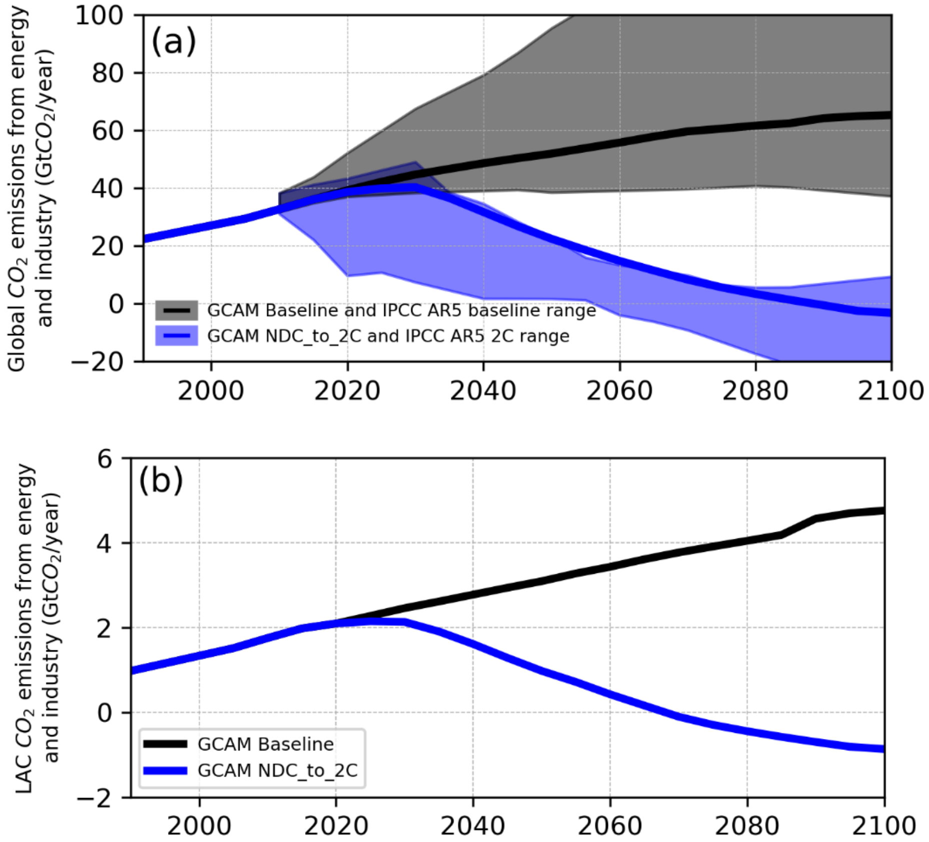


**Supplementary Figure 49.** Global (a) and LAC (b) $CO_2$ emissions from fossil fuel combustion and industrial processes in GCAM NDC_to_2C and Baseline scenarios (solid lines). Shaded areas in (a) represent scenarios included in the AR5 Scenario Database (available at https://secure.iiasa.ac.at/web-apps/ene/AR5DB/; documented in Krey et al.[3]), which maintains the long-term scenarios reviewed in the Fifth Assessment Report (AR5) by the Working Group III of the IPCC. The blue range comprises the subset of policy scenarios that fall within radiative forcing levels consistent with the RCP2.6, and limit global warming until 2100 to less than 2$^{o}$C with at a least a 66% chance[3,4]. The black range is formed by the baseline scenarios (i.e., scenarios that do not include any GHG mitigation policy throughout the century) associated with the mitigation scenarios in the blue range.

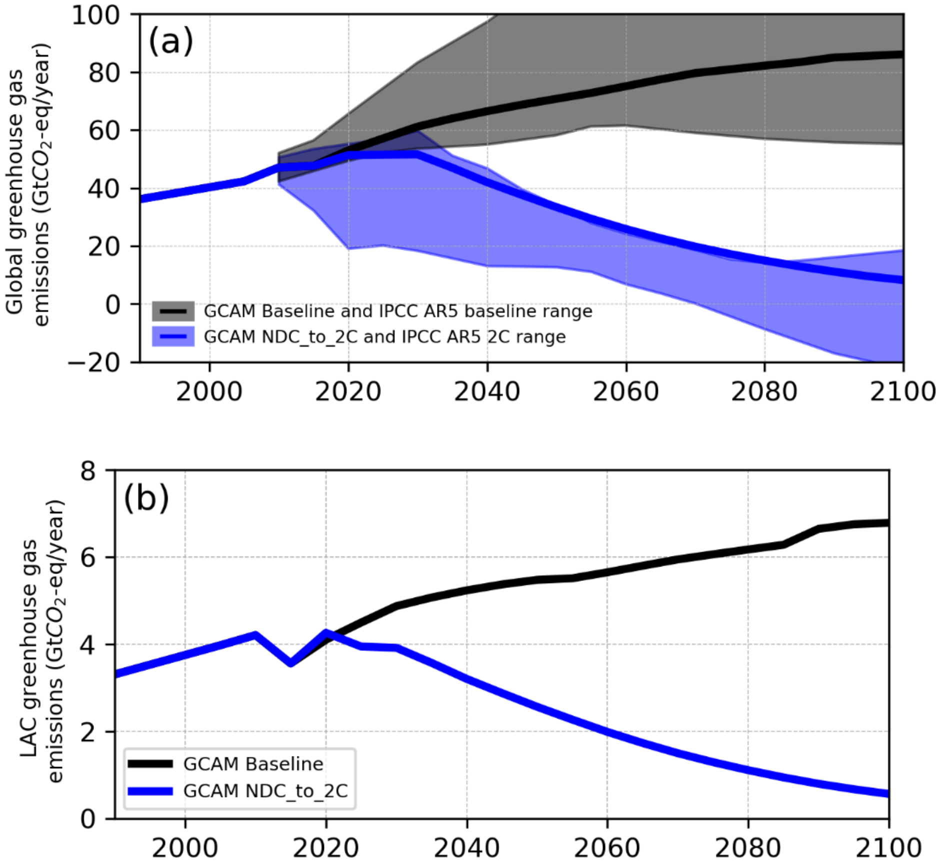


**Supplementary Figure 50.** Global (a) and LAC (b) greenhouse (GHG) gas emissions in GCAM NDC_to_2C and Baseline scenarios (solid lines). Shaded areas in (a) represent scenarios included in the AR5 Scenario Database (available at https://secure.iiasa.ac.at/web-apps/ene/AR5DB/; documented in Krey et al.[3]), which maintains the long-term scenarios reviewed in the Fifth Assessment Report (AR5) by the Working Group III of the Intergovernmental Panel on Climate Change (IPCC). The blue range comprises the subset of policy scenarios that fall within radiative forcing levels consistent with the RCP2.6, and limit global warming until 2100 to less than 2$^{o}$C with at a least a 66% chance[3,4]. The black range is formed by the baseline scenarios (i.e., scenarios that do not include any GHG mitigation policy throughout the century) associated with the mitigation scenarios in the blue range. [Note: $CO_2$-eq emissions include the basket of Kyoto gases (carbon dioxide ($CO_2$), methane ($CH_4$), nitrous oxide ($N_2O$) as well as fluorinated gases) aggregated using 100-year Global Warming Potential values from the IPCC Second Assessment Report.]

## Supplementary Tables

**Supplementary Table 1.** Plans and/or targets regarding renewable energy deployment in nationally determined contributions (NDCs) and long-term strategies (LTSs) submitted by LAC countries to the United Nations Framework Convention on Climate Change (UNFCCC).

| Country | Plans and/or targets regarding renewable energy sources[1] | Climate plan[2] and time coverage | Links |
|---|---|---|---|
| Argentina | Argentina's reduction emissions plan include actions linked to the promotion of biofuels and renewable energy. | NDC: 2025 or 2030 | https://www4.unfccc.int/sites/submissions/INDC/Published%20Documents/Argentina/1/Argentina%20INDC%20Non-Official%20Translation.pdf |
| Antigua and Barbuda | By 2030, 100% of electricity demand in the water sector and other essential services will be met through off-grid renewable sources (conditional contribution[3]). | NDC: 2025 or 2030 | https://www4.unfccc.int/sites/submissions/INDC/Published%20Documents/Antigua%20and%20Barbuda/1/INDC_Antigua_Barbuda.pdf |
| Bahamas | Goal is to achieve a minimum of 30% renewables in the energy mix by 2030 (conditional contribution). | NDC: 2025 or 2030 | https://www4.unfccc.int/sites/submissions/INDC/Published%20Documents/Bahamas/1/Bahamas%20INDC%20Submission.pdf |
| Belize | Belize expects to increase its share of renewable energy in the electricity mix by 85% by 2027 (conditional contribution). | NDC: 2025 or 2030 | https://www4.unfccc.int/sites/submissions/INDC/Published%20Documents/Belize/1/Belize%20INDCS.pdf |
| Bolivia | - Increase in the electric generation capacity through renewables (participation of renewable energy planned to increase to 79% by 2030 from 39% in 2010 with national efforts).<br>- Diversification of the energy matrix through the construction of hydropower (small and medium hydropower plants, large hydro and multipurpose) and boost alternative energy (wind, and biomass, geothermal and solar). | NDC: 2025 or 2030 | https://www4.unfccc.int/sites/submissions/INDC/Published%20Documents/Bolivia/1/INDC-Bolivia-english.pdf |
| Brazil | - Increase in the share of sustainable biofuels in the Brazilian energy mix to approximately 18% by 2030, by expanding biofuel consumption, increasing ethanol supply, including by increasing the share of advanced biofuels (second generation), and increasing the share of biodiesel in the diesel mix.<br>- Goal is to achieve 45% of renewables in the energy mix by 2030, including: expanding the use of renewable energy sources other than hydropower in the total energy mix to between 28% and 33% by 2030; expanding the use of non-fossil fuel energy sources domestically; and increasing the share of renewables (other than hydropower) in the power supply to at least 23% by 2030, by raising the share of wind, biomass and solar. | NDC: 2025 or 2030 | https://www4.unfccc.int/sites/submissions/INDC/Published%20Documents/Bolivia/1/INDC-Bolivia-english.pdf |
| Chile | National plans include target of 20% of the energetic matrix should be made up of non-conventional renewable energies by 2025. | NDC: 2025 or 2030 | https://www4.unfccc.int/sites/submissions/INDC/Published%20Documents/Chile/1/INDC%20Chile%20english%20version.pdf |

| Country | Climate goals and plans regarding renewable energy sources | Climate plan and time coverage | Links |
|---|---|---|---|
| Costa Rica | NDC: Goal is to achieve and maintain a 100% renewable energy matrix by 2030 (conditional contribution).<br><br>LTS: Costa Rica's long-term strategies include 10 "axes of decarbonization", in which four relate to renewable energy:<br><br>1. Development of a mobility system based on safe, efficient and renewable public transport, and on active mobility schemes.<br>2. Transformation of the light-duty vehicles fleet to have zero emissions, sustained by renewable energy.<br>3. Consolidation of the national electric system with capacity, flexibility, intelligence, and resilience necessary to supply and manage renewable energy at competitive cost.<br>4. Transformation of the industrial sector through processes and technologies that use energy from renewable sources or other efficient and sustainable methods that have low or zero-emissions | NDC: 2025 or 2030<br><br>LTS: 2050 | https://www4.unfccc.int/sites/submissions/INDC/Published%20Documents/Costa%20Rica/1/INDC%20Costa%20Rica%20Version%202%200%20final%20ENG.pdf<br><br>https://unfccc.int/sites/default/files/resource/NationalDecarbonizationPlan.pdf |
| Cuba | Cuba plans to build renewable energy capacity composed of 19 bioelectric plants, 13 wind farms with 633 MW, 700 MW of photovoltaic capacity and 74 small hydroelectric power plants (conditional contribution). | NDC: 2025 or 2030 | https://www4.unfccc.int/sites/submissions/INDC/Published%20Documents/Cuba/1/Republic%20of%20Cuba-INDCs-Nov2015.pdf |
| Dominica | By 2030, total emission reductions in the energy industries sector will be 98.6% below 2014 levels principally from harnessing of geothermal resources (conditional contribution). | NDC: 2025 or 2030 | https://www4.unfccc.int/sites/submissions/INDC/Published%20Documents/Dominica/1/Commonwealth%20of%20Dominica-%20Intended%20Nationally%20Determined%20Contributions%20(INDC).pdf |
| Ecuador | Ecuador aims to restructure the energy matrix by, among other criteria, increasing the share of renewable energy. | NDC: 2025 or 2030 | https://www4.unfccc.int/sites/submissions/INDC/Published%20Documents/Ecuador/1/Ecuador%20INDC%2001-10-2015%20-%20english%20unofficial%20translation.pdf |
| El Salvador | Goal is to increase the use of renewables by 2025 (minimum of 12% increase with respect to the total electricity generated in the country in 2014) (conditional contribution). | NDC: 2025 or 2030 | https://www4.unfccc.int/sites/submissions/INDC/Published%20Documents/El%20Salvador/1/EL%20SALVADOR-INTENDED%20NATIONALLY%20DETERMINED%20CONTRIBUTION.pdf |
| Grenada | Grenada plans a 30% reduction in emissions through electricity production by 2025 with 10% from renewables and 20% from energy efficiency measures (conditional contribution). | NDC: 2025 or 2030 | https://www4.unfccc.int/sites/submissions/INDC/Published%20Documents/Grenada/1/Grenada%20INDC.pdf |

| Country | Climate goals and plans regarding renewable energy sources | Climate plan and time coverage | Links |
|---|---|---|---|
| Guatemala | Country intends to promote the use of renewable sources. By 2030, it is expected that electricity generation will be 80% from renewable sources compared to today's level of about 70%. | NDC: 2025 or 2030 | https://www4.unfccc.int/sites/submissions/INDC/Published%20Documents/Guatemala/1/Gobierno%20de%20Guatemala%20INDC-UNFCCC%20Sept%202015.pdf |
| Guyana | Guyana will implement policies to encourage the use of renewable energy. Country's goal is to develop a mix of wind, solar, biomass and hydropower. | NDC: 2025 or 2030 | https://www4.unfccc.int/sites/submissions/INDC/Published%20Documents/Guyana/1/Guyana's%20revised%20iNDC%20-%20Final.pdf |
| Haiti | - Haiti plans to install by 2020, an additional 37.5 MW of hydroelectricity;<br>- conditional contribution: Haiti plans to increase the share of renewable energy in the electricity mix to 47% by 2030 (hydro 24.5%, wind 9.4%, solar 7.5%, biomass 5.6%). | NDC: 2025 or 2030 | https://www4.unfccc.int/sites/submissions/INDC/Published%20Documents/Haiti/1/CPDN_Republique%20d'Haiti.pdf |
| Jamaica | Jamaica plans to increase the share of renewable sources of energy in its primary energy mix to 20% by 2030. | NDC: 2025 or 2030 | https://www4.unfccc.int/sites/submissions/INDC/Published%20Documents/Jamaica/1/Jamaica's%20INDC_2015-11-25.pdf |
| Mexico | Mexico's long-term strategies include "lines of action" intended to:<br>- encourage wind power and to benefit from its onshore and offshore potential;<br>- promote investment in photovoltaic systems in high potential regions of the country;<br>- encourage distributed generation by using photovoltaic systems in industrial, residential, and service sectors;<br>- harness existing electric power potential by installing new large hydropower plants; and<br>- encourage power generation in small, mini, and micro hydroelectric plants. | LTS: 2050 | https://unfccc.int/files/focus/long-term_strategies/application/pdf/mexico_mcs_final_cop22nov16_red.pdf |
| Panama | Panama plans to increase the percentage of electricity generation from solar, wind and biomass to 30% in 2050 compared to 2014. According to this plan, the participation of these renewable energy sources in the electrical matrix will be 15% by 2030. | NDC: 2025 or 2030 | https://www4.unfccc.int/sites/submissions/INDC/Published%20Documents/Panama/1/Panama_NDC.pdf |
| Paraguay | - Incorporation of technologies for the exploitation of new sources of sustainable energy (includes solar, wind and biomass energy).<br>- Increase in the consumption of renewable energy by 60% (% participation in the energy matrix). | NDC: 2025 or 2030 | https://www4.unfccc.int/sites/submissions/INDC/Published%20Documents/Paraguay/1/Documento%20INDC%20Paraguay%2001-10-15.pdf |
| Saint Kitts and Nevi | Goal is increase the use of renewable energy sources by 50% by 2030 (conditional contribution). | NDC: 2025 or 2030 | https://www4.unfccc.int/sites/submissions/INDC/Published%20Documents/Saint%20Kitts%20and%20Nevis/1/St.%20Kitts%20and%20Nevis%20INDC.pdf |
| Saint Lucia | 35% Renewable Energy Target by 2025 and 50% by 2030 based on a mix of geothermal, wind and solar energy sources (conditional contribution). | NDC: 2025 or 2030 | https://www4.unfccc.int/sites/submissions/INDC/Published%20Documents/Saint%20Lucia/1/Saint%20Lucia's%20INDC%2018th%20November%202015.pdf |

| Country | Climate goals and plans regarding renewable energy sources | Climate plan and time coverage | Links |
|---|---|---|---|
| Saint Vincent and Grenadines | Country is renovating existing hydropower facilities to improve efficiency and generation capacity as well as enabling and encouraging the installation of small-scale photovoltaics in the private and public sectors. | NDC: 2025 or 2030 | https://www4.unfccc.int/sites/submissions/INDC/Published%20Documents/Saint%20Vincent%20and%20Grenadines/1/SVG_INDC_Final.pdf |
| Suriname | NDC states that several initiatives are already in an advanced stage such as solar energy for communities in the hinterland, a study on waste-to-energy at the national landfill, and micro-hydro power projects in the Interior. Other forms of renewable energy to be explored are wind energy as well as biomass-to-energy. | NDC: 2025 or 2030 | https://www4.unfccc.int/sites/submissions/INDC/Published%20Documents/Suriname/1/INDC-2-Suriname%20300915.pdf |
| Uruguay | - In the NDC dated from 2015, the country expected to achieve in 2017 an absolute emissions reduction of 88% within the power sector compared to the 2005-2009 annual average. This would be achieved with 40% of non-conventional renewable energy sources (mainly wind, but also photovoltaic and biomass waste), in addition to 55% hydropower.<br>- For the following decades, NDC states that power sector emissions could remain close to the 2017 value if storage systems were to be incorporated through additional means of implementation to be provided by external sources (conditional contribution). | NDC: 2025 or 2030 | https://www4.unfccc.int/sites/submissions/INDC/Published%20Documents/Uruguay/1/INDC%20Uruguay%20(English-unofficial%20translation).pdf |

**Notes:**

1. Plans vary largely in the level of details reported, and can include just a general objective, specific lines of actions or quantitative targets.
2. At the time of this writing, only two LAC countries have submitted LTSs: Costa Rica and Mexico.
3. A conditional contribution is contingent on international support in the form of finance, investment, technology development and transfer and capacity-building for its fulfilment. UNFCCC parties submitted NDCs (1) without any requirement of external support (unconditional NDCs), (2) entirely dependent on international support (conditional NDCs), and (3) including both unconditional and conditional contributions.

**Supplementary Table 2.** Mean change (%) in electricity generation by RE source under the *RCP26_FullTech: Combined impacts* scenario. Changes represent the mean value across GCMs and are relative to the *RCP26_FullTech: No-climate impacts* scenario. As mentioned in the Supplementary Note 6, each RE climate impact was implemented individually in GCAM.

| | | | | |
|---|---|---|---|---|
| | | **2050** | | |
| | **Biomass** | **Hydropower** | **Solar** | **Wind** |
| Brazil | -0.42 | 4.71 | 0.24 | 12.98 |
| C. Am/Car. | -1.62 | -0.09 | -0.15 | -5.02 |
| Mexico | -1.64 | -0.70 | -0.23 | -2.65 |
| S. Am. (N) | 0.24 | -5.89 | -0.32 | 8.23 |
| S. Am. (S) | -0.11 | 1.74 | -0.25 | 10.22 |
| Argentina | -3.07 | 0.35 | -0.25 | -1.90 |
| Colombia | -4.95 | -2.27 | 0.60 | -2.13 |
| Uruguay | -0.03 | 10.85 | -0.31 | -0.12 |
| | | **2100** | | |
| | **Biomass** | **Hydropower** | **Solar** | **Wind** |
| Brazil | -0.26 | 7.91 | 0.40 | 15.95 |
| C. Am/Car. | 0.41 | 2.07 | -0.17 | -5.43 |
| Mexico | -0.25 | 2.04 | -0.06 | 3.21 |
| S. Am. (N) | 0.81 | -4.87 | -0.20 | 15.35 |
| S. Am. (S) | 0.53 | 4.85 | -0.33 | 16.57 |
| Argentina | -0.40 | 1.61 | -0.37 | -2.48 |
| Colombia | -0.03 | -6.12 | 0.47 | 0.09 |
| Uruguay | 0.11 | 17.16 | 0.07 | -0.05 |

**Supplementary Table 3.** Mean change (%) in electricity generation by RE source under the *RCP26_NoCCS & NoNewNuc: Combined impacts* scenario. Changes represent the mean value across GCMs and are relative to the *RCP26_NoCCS & NoNewNuc: No-climate impacts* scenario. As mentioned in the Supplementary Note 6, each RE climate impact was implemented individually in GCAM.

| | **2050** | | | |
|---|---|---|---|---|
| | **Biomass** | **Hydropower** | **Solar** | **Wind** |
| Brazil | -0.03 | 4.71 | 0.31 | 15.23 |
| C. Am/Car. | -0.93 | -0.09 | 0.05 | -4.92 |
| Mexico | -0.53 | -0.70 | -0.19 | -0.09 |
| S. Am. (N) | 0.40 | -5.89 | -0.29 | 9.76 |
| S. Am. (S) | -0.06 | 1.74 | -0.17 | 14.52 |
| Argentina | -1.55 | 0.35 | -0.29 | -1.25 |
| Colombia | -0.81 | -2.26 | 0.63 | -2.21 |
| Uruguay | 0.17 | 10.85 | -0.38 | -0.12 |
| | **2100** | | | |
| | **Biomass** | **Hydropower** | **Solar** | **Wind** |
| Brazil | 0.25 | 7.91 | 0.20 | 14.19 |
| C. Am/Car. | -0.05 | 2.07 | 0.50 | -5.36 |
| Mexico | 0.13 | 2.04 | 0.41 | -0.87 |
| S. Am. (N) | 1.47 | -4.87 | 0.30 | 18.51 |
| S. Am. (S) | 0.81 | 4.85 | -0.02 | 24.35 |
| Argentina | -0.49 | 1.61 | -0.15 | -0.52 |
| Colombia | 0.63 | -6.11 | 0.23 | 0.05 |
| Uruguay | 0.64 | 17.16 | 0.28 | -0.08 |

**Supplementary Table 4.** LAC countries per GCAM region (note: GCAM-LAC version)

| GCAM region | Country |
|---|---|
| Argentina | Argentina |
| Brazil | Brazil |
| Central America and Caribbean | Aruba, Anguilla, Netherlands Antilles, Antigua & Barbuda, Bahamas, Belize, Bermuda, Barbados, Costa Rica, Cuba, Cayman Islands, Dominica, Dominican Republic, Guadeloupe, Grenada, Guatemala, Honduras, Haiti, Jamaica, Saint Kitts and Nevis, Saint Lucia, Montserrat, Martinique, Nicaragua, Panama, El Salvador, Trinidad and Tobago, Saint Vincent and the Grenadines |
| Colombia | Colombia |
| Mexico | Mexico |
| South America_Northern | French Guiana, Guyana, Suriname, Venezuela |
| South America_Southern | Bolivia, Chile, Ecuador, Peru, Paraguay |
| Uruguay | Uruguay |

**Supplementary Table 5.** Model Parameters Updated in GCAM-LAC

| Sector \| Category | Regions | Parameter(s) Affected | GCAM-LAC Input File(s)[1] |
|---|---|---|---|
| Socioeconomics | Argentina, Colombia and Uruguay | Population and GDP assumptions | Argentina_refPop_gSSP2.xml \| Colombia_refPopGDP_gSSP2.xml \| Uruguay_refPopGDP_socioeconomics_gSSP2.xml |
| Energy sector \| Electricity: hydropower | Colombia and Uruguay | Hydroelectricity production (EJ) per GCAM LAC region and time step | Colombia_ref_hydro.xml \|Uruguay_refElecHydro.xml |
| Water sector \|Livestock | Colombia | Coefficient of water demand for livestock production per types/technologies of animal production | Colombia_ref_livestock_water_demand_coeff.xml |
| Energy sector \| Electricity | Global | Share-weight[2] interpolation rule for the Natural gas (combined-cycle) technology | LACglobal_ref_gas_cc_sw_interp.xml |
| Energy sector \| Final Energy Consumption: Transportation | Global | Inclusion of battery electric vehicle (BEV) technology in "Freight Truck" mode | LACglobal_ref_transportation_UCD_CORE.xml |
| Energy sector \| Final Energy Consumption: Transportation | Global | Inclusion of battery electric vehicle (BEV) technology in "Passenger Bus" mode | LACglobal_ref_transportation_UCD_CORE.xml |
| Energy sector \| Final Energy Consumption: Transportation | Global | Updated input-costs for liquids and natural gas technologies in "Freight Truck" mode | LACglobal_ref_transportation_UCD_CORE.xml |

| Sector \| Category | Regions | Parameter(s) Affected | GCAM-LAC Input File(s)[1] |
|---|---|---|---|
| Energy sector \| Final Energy Consumption: Transportation | Global | Updated input-costs for liquids and natural gas technologies in "Passenger Bus" mode | LACglobal_ref_transportation_UCD_CORE.xml |
| Energy sector \| Final Energy Consumption: Transportation | Global | Updated share-weights to allow higher preference for BEV technology in "Passenger Light duty vehicle (LDV)" mode and to gradually reduce preference for liquids and natural gas technologies | LACglobal_ref_transportation_UCD_CORE.xml |
| Water sector \| Primary Energy | Colombia | Water demand coefficients (consumption and withdrawal) of primary energy production for the following fuels: coal, oil (conventional and unconventional), natural gas, and uranium | Colombia_ref_L210.TechCoef_mod.xml |
| Energy sector \| Electricity | Colombia | Share-weight interpolation rule for nuclear, geothermal and coal sources | Colombia_ref_L223.SubsectorShrwt.xml |
| Water sector \| Industry Manufacturing | Colombia | Industrial manufacturing sector's water demand coefficients (withdrawal) | Colombia_ref_L232.TechCoef_mod.xml |
| Water sector \| Agriculture | Colombia | Water withdrawal coefficients by crops, land unit and irrigation management option ("irrigation_high" and "irrigation_low") | Colombia_ref_L2072.AgCoef_IrrWaterWdraw_ag_mgmt_mod2.xml |
| Energy Sector \| Energy Final Demand | Colombia | Cement income-elasticity[3] (affects cement demand responses to changes in income) | Colombia_refPopGDP_cement_incelas_gssp2.xml |
| Land sector | Colombia | Demand-related parameters (Income-elasticity and price-elasticity[4]) of agricultural products ("Food" and "NonFood" categories) | Colombia_refPopGDP_demand_input.xml |

| Sector \| Category | Regions | Parameter(s) Affected | GCAM-LAC Input File(s)[1] |
|---|---|---|---|
| Energy Sector \| Energy Final Demand | Colombia | Industry income-elasticity (affects industry-sector demand responses to changes in income) | Colombia_refPopGDP_industry_incelas_gssp2.xml |
| Energy sector \| Electricity | Global | Share-weights and share-weight interpolation rules for coal, natural gas and refined liquids; Implementation of the structure of the electricity sector for Uruguay (supply sectors, subsectors and technology parameters specified in each period) | LACglobal_ref_electricity_water.xml |
| Energy sector \|Electricity | Uruguay | Share-weight interpolation rule for refined liquids, geothermal, gas, nuclear, coal, solar, wind and biomass sources | Uruguay_refElecShareWeights_L223.SubsectorShrwt.xml |
| Energy Sector \| Energy Final Demand | Uruguay | Parameters related to the final demand of energy by end-use sectors: 1) income elasticities for the buildings, industry, transportation sectors and cement production; 2) share-weights for the industrial energy use sector (divided in electricity, refined liquids and biomass), and for the residential and commercial sectors by aggregate services (heating, cooling, and other) | Uruguay_refFinalNrg.xml |
| Energy Sector \| Energy Final Demand | Uruguay | Buildings Income-elasticity | Uruguay_refPopGDP_bld_agg_gSSP2.xml |

| Sector \| Category | Regions | Parameter(s) Affected | GCAM-LAC Input File(s)[1] |
|---|---|---|---|
| Energy Sector \| Energy Final Demand | Uruguay | Cement income-elasticity | Uruguay_refPopGDP_cement_incelas_gssp2.xml |
| Land sector | Uruguay | Demand-related parameters (Income-elasticity and price-elasticity) of agricultural products ("Food" and "NonFood" categories) | Uruguay_refPopGDP_demand_input.xml |
| Energy Sector \| Energy Final Demand | Uruguay | Industry income-elasticity | Uruguay_refPopGDP_industry_incelas_gssp2.xml |
| Energy Sector \| Energy Final Demand | Uruguay | Transportation income-elasticity | Uruguay_refPopGDP_trn_agg_gSSP2.xml |
| Water sector \| Agriculture | Uruguay | Water consumption coefficients by crops, land unit and irrigation management option ("irrigation_high" and "irrigation_low") | Uruguay_refWat_L2072.AgCoef_IrrWaterCons_ag_mgmt.xml |
| Water sector \| Agriculture | Uruguay | Water withdrawal coefficients by crops, land unit and irrigation management option ("irrigation_high" and "irrigation_low") | Uruguay_refWat_L2072.AgCoef_IrrWaterWdraw_ag_mgmt.xml |
| Water sector \| Industry Manufacturing | Uruguay | Industrial manufacturing sector's water demand coefficients (consumption and withdrawal) | Uruguay_refWater.xml |

**Notes:** [1] GCAM-LAC model can be downloaded from http://doi.org/10.5281/zenodo.4048788. The .xml files listed above are located at: <path_to_GCAM-LAC_folder>/input/idb/reference.

[2] "Share-weight" is a calibration parameter used in GCAM to provide for new technologies to be phased in gradually and to allow gradual movement away from the base year's calibrated share weight values.

[3] Income-elasticity represents the percent change in demand of a good divided by the percent change in GDP. This parameter affects how the demand for a good responds to changes in income.

[4] Price-elasticity represents the percent change in demand of a good divided by the percent change in the price. This parameter affects how the demand for a good responds to changes in price.

**Supplementary Table 6.** NDCs quantification in LAC*

| GCAM Region | Country | 2010 GHG emissions ($MtCO_2e$) | GHG percent change relative to 2010 levels | Emissions assumption in NDC ($MtCO_2e$) | Year |
|---|---|---|---|---|---|
| Argentina | Argentina | 389.4 | 20% | 467.3 | 2030 |
| Brazil | Brazil | 1285.0 | -1% | 1272.2 | 2025 |
| Central America and Caribbean | Costa Rica | 5.2 | -28% | 3.7 | 2030 |
| | Dominican Republic | 31.7 | -25% | 23.8 | 2030 |
| | Grenada | 1.8 | -30% | 1.3 | 2025 |
| Colombia | Colombia | 224.0 | 5% | 235.2 | 2030 |
| Mexico | Mexico | 746.0 | -5% | 708.7 | 2030 |
| South America Northern | Venezuela | 200 | 36% | 272.0 | 2030 |
| South America Southern | Chile | 84.9 | 11% | 94.2 | 2030 |
| | Peru | 170.6 | 22% | 208.1 | 2030 |

*Notes:

1. NDCs quantification based on Vrontisi et al.[5].
2. Table only includes countries with quantifiable NDCs in terms of percent changes in emissions relative to 2010 levels. For this reason, Ecuador, which is listed in Vrontisi et al., is not represented.
3. When implemented in GCAM, it is assumed that NDCs cover GHGs from all sectors, including $CO_2$ emissions from land-use and land-cover change.

**Supplementary Table 7.** Summary of general assumptions for the NDC 2020-2030 period*

<table>
<tr><th>GCAM Region</th><th>Country</th><th>2020 GHG emissions</th><th>2025 GHG emissions</th><th>2030 GHG emissions</th></tr>
<tr><td>Argentina</td><td>Argentina</td><td rowspan="10">GHG emissions follow reference scenario</td><td>linear interpolation between 2020 and 2030 levels</td><td>Vrontisi et al (2018)</td></tr>
<tr><td>Brazil</td><td>Brazil</td><td>Vrontisi et al (2018)</td><td>Assumed 1200 $MtCO_2e$ as in Brazil's NDC[1]</td></tr>
<tr><td rowspan="3">Central America and Caribbean</td><td>Costa Rica</td><td rowspan="2">linear interpolation between 2020 and 2030 levels</td><td rowspan="2">Vrontisi et al (2018)</td></tr>
<tr><td>Dominican Republic</td></tr>
<tr><td>Grenada</td><td>Vrontisi et al (2018)</td><td>GHG emissions follow reference scenario</td></tr>
<tr><td>Colombia</td><td>Colombia</td><td rowspan="5">linear interpolation between 2020 and 2030 levels</td><td rowspan="5">Vrontisi et al (2018)</td></tr>
<tr><td>Mexico[2]</td><td>Mexico</td></tr>
<tr><td>South America Northern</td><td>Venezuela</td></tr>
<tr><td rowspan="2">South America Southern</td><td>Chile</td></tr>
<tr><td>Peru</td></tr>
<tr><td>Globe</td><td>–</td><td>52.71 $GtCO_2e$[3]</td><td>linear interpolation between 2020 and 2030 levels</td><td>53.7 $GtCO_2e$ (Vrontisi et al (2018))</td></tr>
</table>

*Notes:

1. Brazil's NDC submission available at: https://www4.unfccc.int/sites/submissions/INDC/Submission%20Pages/submissions.aspx

2. Following Vrontisi et al.[5], Mexico is assumed not to achieve its Copenhagen pledge.

3. Average level between the “High” and “Low” global GHG emissions estimated by Riahi et al.[6] as resulting from 2020 Copenhagen pledges.

**Supplementary Table 8.** Technology capacity factor assumptions

| Technology | Capacity Factor |
|---|---|
| Biomass (conv) | 0.85 |
| Biomass (IGCC) | 0.8 |
| Biomass (conv CCS) | 0.85 |
| Biomass (IGCC CCS) | 0.8 |
| Coal (conv pul) | 0.85 |
| Coal (IGCC) | 0.8 |
| Coal (conv pul CCS) | 0.8 |
| Coal (IGCC CCS) | 0.8 |
| Gas (CC) | 0.85 |
| Gas (steam/CT) | 0.8 |
| Gas (CC CCS) | 0.8 |
| Refined liquids (steam/CT) | 0.8 |
| Refined liquids (CC) | 0.85 |
| Refined liquids (CC CCS) | 0.8 |
| Gen II LWR (Nuclear) | 0.9 |
| Gen III (Nuclear) | 0.9 |
| CSP | varies by region (Supplementary Table 9) |
| CSP with storage | varies by region (Supplementary Table 9) |
| PV | varies by region (Supplementary Table 9) |
| PV with storage | varies by region (Supplementary Table 9) |
| Wind | varies by region (Supplementary Table 9) |
| Wind with storage | varies by region (Supplementary Table 9) |
| Rooftop PV | varies by region (Supplementary Table 9) |
| Geothermal | 0.9 |
| Hydropower | 0.54 |

**Supplementary Table 9.** Technology capacity factor assumptions in the LAC region

| GCAM-LAC Region | Capacity Factor by Technology | | | | | | |
|---|---|---|---|---|---|---|---|
| | CSP | CSP with storage | PV | PV with storage | Wind | Wind with storage | Rooftop PV |
| Argentina | 0.25 | 0.65 | 0.23 | 0.23 | 0.37 | 0.37 | 0.23 |
| Brazil | 0.25 | 0.65 | 0.25 | 0.25 | 0.34 | 0.34 | 0.25 |
| Central America and Caribbean | 0.25 | 0.65 | 0.25 | 0.25 | 0.38 | 0.38 | 0.25 |
| Colombia | 0.25 | 0.65 | 0.22 | 0.22 | 0.32 | 0.32 | 0.22 |
| Mexico | 0.25 | 0.65 | 0.27 | 0.27 | 0.38 | 0.38 | 0.27 |
| South America_Northern | 0.25 | 0.65 | 0.24 | 0.24 | 0.35 | 0.35 | 0.24 |
| South America_Southern | 0.25 | 0.65 | 0.24 | 0.24 | 0.36 | 0.36 | 0.24 |
| Uruguay | 0.25 | 0.65 | 0.20 | 0.20 | 0.37 | 0.37 | 0.20 |

**Supplementary Table 10.** Electric power sector capital cost assumptions (2010$/kW)

| | **2020** | **2030** | **2050** | **2100** |
|---|---|---|---|---|
| Biomass (conv)* | 3951 | 3861 | 3703 | 3425 |
| Biomass (IGCC)* | 5746 | 5339 | 4819 | 4335 |
| Biomass (conv CCS)* | 7318 | 6766 | 6169 | 5665 |
| Biomass (IGCC CCS)* | 8338 | 7579 | 6721 | 6059 |
| Coal (conv pul)* | 2863 | 2799 | 2686 | 2482 |
| Coal (IGCC)* | 3832 | 3560 | 3215 | 2889 |
| Coal (conv pul CCS)* | 5504 | 5078 | 4619 | 4238 |
| Coal (IGCC CCS)* | 6195 | 5604 | 4945 | 4464 |
| Gas (CC)* | 1036 | 1014 | 972 | 901 |
| Gas (steam/CT)* | 742 | 723 | 694 | 642 |
| Gas (CC CCS)* | 1992 | 1837 | 1672 | 1533 |
| Refined liquids (steam/CT)* | 742 | 723 | 694 | 642 |
| Refined liquids (CC)* | 1036 | 1014 | 972 | 901 |
| Refined liquids (CC CCS)* | 2356 | 2153 | 1937 | 1775 |
| Gen II LWR (Nuclear)* | 5501 | 5501 | 5501 | 5501 |
| Gen III (Nuclear)* | 5433 | 5307 | 5094 | 4710 |
| CSP* | 4367 | 3770 | 3199 | 2905 |
| CSP with storage* | 7431 | 6617 | 5772 | 5216 |
| PV | 1788 | 1662 | 1501 | 1349 |
| PV with storage | 4213 | 3916 | 3535 | 3180 |
| Wind | 1914 | 1778 | 1608 | 1446 |
| Wind with storage | 5555 | 5162 | 4661 | 4190 |
| Rooftop PV | 4500 | 4183 | 3777 | 3396 |
| Geothermal* | 4348 | 4248 | 4074 | 3767 |
| Hydropower | 2600 | 2600 | 2600 | 2600 |

*For these generation technologies, the final overnight capital costs increase depending on the cooling technology deployed. The following cooling system options are available in GCAM: once through, seawater, recirculating, cooling pond and dry cooling. These cooling technologies can add between 19 (once through) and 200 (dry cooling) 2010$/KW to the final overnight capital costs of electricity generation technologies. However, the base costs presented in the table represent the major portion of the total overnight capital costs.

**Supplementary Table 11.** Regionally aggregated changes in total capital investments in the LAC electric power sector under the *Combined impacts* scenarios. Changes represent the mean value (absolute and percentage) across GCMs (the standard deviation of the absolute model mean change is also shown), and are calculated using cumulative investments in the 2020 ─ 2050 period. Changes are relative to the *No-climate impacts* scenarios (i.e., positive values mean that scenarios with climate impacts on renewables show increased costs).

| Region | RCP60_Baseline | | | RCP26_FullTech | | | RCP26_NoCCS & NoNewNuc | | |
|---|---|---|---|---|---|---|---|---|---|
| | 2050 | | | 2050 | | | 2050 | | |
| | Mean ($Bill.) | Mean (%) | Std. ($Bill.) | Mean ($Bill.) | Mean (%) | Std. ($Bill.) | Mean ($Bill.) | Mean (%) | Std. ($Bill.) |
| **Brazil** | 6.04 | 2.06 | 6.92 | 6.46 | 1.21 | 3.13 | 23.82 | 7.05 | 18.05 |
| **Central America and Caribbean (C. Am/Car.)** | -0.07 | -0.03 | 0.47 | 0.74 | 0.23 | 3.76 | -2.35 | -0.82 | 3.14 |
| **Mexico** | 0.17 | 0.09 | 1.76 | -1.62 | -0.41 | 7.01 | -4.12 | -1.22 | 14.99 |
| **South America_Northern (S. Am. (N))** | 0.22 | 0.28 | 8.13 | 0.59 | 0.38 | 2.93 | 1.61 | 1.14 | 3.77 |
| **South America_Southern (S. Am. (S))** | 0.51 | 0.28 | 2.44 | 3.89 | 1.45 | 1.15 | 11.76 | 5.18 | 0.63 |
| **Argentina** | -1.04 | -1.08 | 0.25 | -2.05 | -0.96 | 0.23 | -4.67 | -1.79 | 0.37 |
| **Colombia** | -0.43 | -0.56 | 0.51 | -0.46 | -0.33 | 2.21 | -0.38 | -0.34 | 1.03 |
| **Uruguay** | -0.07 | -0.46 | 0.20 | -0.23 | -1.07 | 0.17 | -0.20 | -0.99 | 0.42 |
| **LAC** | 5.34 | 0.45 | 14.22 | 7.32 | 0.36 | 8.51 | 25.46 | 1.48 | 18.63 |

## Supplementary Notes

### Supplementary Note 1. Climate change impacts on the renewable energy supply and potential effects in the Latin America and the Caribbean region

A brief review on major climate change effects on the RE supply follows. For a more comprehensive review focusing on the whole energy sector, including other relevant aspects such as climate effects on thermoelectric efficiency and transmission systems, extreme events, among others, the reader is referred to Schaeffer et al.[7], Cronin et al.[8] and Solaun et al.[9]. We aim at emphasizing the impacts projected for Latin America and the Caribbean (LAC) published on peer-reviewed articles after 2010, albeit somewhat limited by the weak literature coverage in the region. Such a gap is evident in the literature reviews by Cronin et al.[8] and Solaun et al.[9], which show a predominance of regional studies on climate impacts on the energy sector focusing on Europe and North America.

### Bioenergy

Assessing climate impacts on the agricultural system, that can affect biomass production, is complex due to the potential plants exposure to a range of biological and environmental stresses, and to the large uncertainty within the impacts modeling chain that involves general circulation model (GCM) outputs driving responses of crop models spanning different structures, assumptions and approaches. Overall, changes in agricultural yields can be positive or negative depending on the warming levels, rainfall changes and $CO_2$ fertilization with responses varying widely by crop type and region. $CO_2$ fertilization is acknowledged as a key modulating mechanism that can partially offset additional plants transpiration requirements by promoting increased water use efficiency[10]. Although there might be some benefits for certain crops at mid- and high-latitude zones at moderate-to-medium levels of local warming (1-3$^{o}$C), for a wide range of regions and crops, impacts tend to be predominantly negative, particularly in the tropical regions[4,11]. Under higher warming levels, crops can also be more susceptible to deleterious effects associated with plant diseases and pest outbreaks, elevated tropospheric ozone concentration and higher risk of occurrence of extreme events (i.e., heat stress, droughts, floods)[12].

In LAC, the two most important bioenergy feedstocks are sugarcane and soybeans employed in bioethanol and biodiesel production, respectively. According to the last Intergovernmental Panel on Climate Change (IPCC) report[13], both crops are, in general, likely to respond positively to $CO_2$ concentration and temperature changes projected for the region, even considering a decrease in water availability. However, a large variability of impacts at smaller sub-regional scales is expected. For example, Marin et al.[14] used a sugarcane growth model that includes $CO_2$ fertilization forced by downscaled outputs from two GCMs under high and low emissions scenarios of the IPCC Special Report on Emissions Scenarios (SRES), and found increases in productivity and better water use efficiency for rainfed sugarcane in southern Brazil in all scenarios. Projected yield increases in this region (which is currently the main sugarcane plantation area in the country) ranges between 15 and 59% in 2050 relative to present-day average yield. On the other hand, the modeling study of Carvalho et al.[15], focusing on a subdomain within the northeastern Brazil (a region considered particularly vulnerable to climate

change where droughts events are recurrent), found sugarcane yield reductions by 2040 and 2100 for the moderate A1B SRES emissions scenario. Rolla et al.[16] employed the DSSAT cropping system model forced by the CCSM4 climate model, and found significant yield increases of rainfed soybean crops in Central Argentina for the near (2015–2039) and far (2075-2099) horizons relative to present-day conditions under representative concentration pathways (RCPs) 4.5 and 8.5. The yield increases were found to be associated with projected increases in summer rainfalls in the region.

**Hydropower**

Climate change can modify surface runoff owing to shifts in mean annual and seasonal precipitation, in evapotranspiration patterns and in the amount and seasonal cycle of snowmelt [7], which would affect the mean river-flow and flow seasonality. In addition, more frequent flooding events may affect the safety of dams' infrastructure. There is also great complexity in assessing climate impacts on hydropower that relate to very region-specific climate responses and local interactions with socioeconomic agents that compete for water resources [17]. Moreover, the inherent GCMs uncertainty adds up to those arising from distinct global hydrological models (GHMs). The latter can result in a larger spread in simulated streamflow than the spread originating from GCMs, as noted by van Vliet et al.[18] and references therein.

Due to the strong non-uniform distribution of projected temperature and precipitation changes around the globe [4], current understanding is that hydropower potentials may increase in certain areas whereas other regions may face a decline. For instance, the study by Hamududu and Killingtveit (2012) [19] project small global and regional changes in hydropower generation by 2050, with slight increases in some regions (e.g., Americas by 0.05%), and reductions in other areas (e.g., Europe by –0.16%). Nevertheless, many of the global and regional studies summarized by Cronin et al. and Solaun et al.[8,9] point out to larger climate-induced changes in runoff, streamflow, hydropower potentials or generation depending on the study scope. According to Cronin et al.[8], positive impacts tend to be located in high latitude areas (e.g., Canada, Russia, northern Europe, northeast China) whereas negative impacts tend to be located in regions such as southern Europe, southern USA, southeast China and southern South America.

Within the LAC domain, the review by Solaun et al.[9] indicates potential hydropower generation losses in Brazil, Chile, Colombia and Costa Rica, and substantial uncertainties surrounding Ecuador's hydrological projections. In the particular case of Brazil (one of the largest hydroelectricity producers in the world), a recent study by Ruffato-Ferreira et al.[20] used the Eta regional model to dynamically downscale projections from the global model HadGEM2-ES under RCPs 4.5 and 8.5. By computing the variation of the water balance index (function of precipitation minus evapotranspiration rates) up to 2100 for the eight main watersheds in the country, the study highlighted a decreasing trend in water availability for watersheds located in the north and center of the country and an increasing trend for southern watersheds in both RCPs. The most pronounced trend of increased water scarcity was found for the northeastern Brazil. In face of a potential marked sub-regional variation of impacts on water availability – as suggested by the study of Ruffato-Ferreira et al.[20] – it can be expected that the net effect of climate change on the total hydropower potential in Brazil will depend on the location of the installed capacity as well as on the planned capacity additions. Currently, the majority of

hydroelectric power plants are in the Midwest and Southern regions, with the Southwest region accounting for 70% of the hydropower storage capacity[21]. However, the current government plans of hydropower expansion in Brazil for the next decade focus on the Northwest (i.e., Amazon) region[22].

As noted in the main text, climate impacts on hydropower have been represented in IAMs. For example, Turner et al.[23] forced a coupled global hydrological and dam model with an ensemble of 16 GCMs (RCPs 4.5 and 8.5), incorporating the projected changes in hydropower potentials in the Global Change Analysis Model (GCAM). The multi-model mean response signaled losses in future hydropower generation in northern South America, Argentina, southern South America, Colombia, and Brazil, albeit with a lack of agreement in the direction of changes for the latter three regions. This same methodology and GCM forcings were employed in regional studies covering Brazil [24] and Colombia [25], in which the median changes in hydropower generation up to the mid-century resulted to be negative. However, the spread in simulated 2050 hydropower generation was large, spanning from -13% to +4% in RCP 4.5, and -12% to 2% in RCP 8.5 in Brazil, and from about -20% to 0% for both RCPs in Colombia. In Uruguay, the modeling framework of Zarrar et al.[26] (based on hydrology simulations forced by 5 GCMS and 4 RCPs incorporated in GCAM) projected increases in runoff and hydropower by 2050 across the 20 simulations performed.

**Solar**

Solar large-scale electricity generation comprises two main technologies: photovoltaics (PV) and concentrating solar power (CSP). In both cases, the primary climate impact derives from alterations in the spatial and temporal distribution of the incident solar radiation at the surface. Downwelling solar radiation is attenuated through absorption by atmospheric gases (in which water vapor plays an important role) and aerosol particles. Moreover, the incident radiative flux is attenuated through scattering by aerosols, cloud droplets and ice crystals. The aerosol burden of the atmosphere can also influence the amount of incident solar radiation indirectly by affecting cloud properties such as cloud albedo and lifetime. All these atmospheric constituents are subject to changes due to the anthropogenic interference on the climate system. Overall, CSP systems are considered to be more vulnerable than the PV counterparts since the former relies exclusively on the direct component of solar radiation, whereas the latter utilizes both direct and diffuse solar radiation [17]. Within a global perspective, multi-model end-of-century projections from the Coupled Model Intercomparison Project phase 5 (CMIP5) show consistent reductions in annual mean total cloud cover in low to mid-latitudes under increasing warming levels, notably the RCP8.5 [27]. However, other effects also play relevant roles with fundamental differences between both technologies.

PV systems rely on panels or modules connecting several PV cells manufactured from a semiconductor material, predominantly silicon. For this technology, increasing mean temperatures negatively affect cell efficiency reducing power output. In general, PV cell efficiency drops approximately 0.5% per 1$^{o}$C increase considering PV technologies such as crystalline silicon and thin-film modules [17]. PV efficiency is also influenced by the wind flow around the module, which produces a cooling effect [28]. CSP facilities operate with an array of typically hundreds of high-magnification mirrors or lenses that focus the sun’s direct beam

radiation on thermally efficient receivers, thereby heating a working fluid that is used to drive steam turbines. CSP outputs increase almost linearly in response to ambient temperature, but the dominant effect lies on the response to solar radiation changes [29]. Apart from the potential to reduce the amount of incident solar radiation, increased levels of dust and anthropogenic particulate matter being deposited on PV panels and CSP collectors diminish electricity generation[30].

The three following global-scale studies have used large GCM CMIP5 ensembles to investigate climate impacts on PV and CSP outputs, addressing specifically the RCP8.5. Wild et al.[31] found that the temperature effect is likely to compensate gains from an increase in incoming surface solar radiation, leading to negative trends in PV power output by the midcentury in most parts of the world. Overall, the projected power changes between 2006 and 2049 were about 1%/decade. In contrast, the midcentury CSP output was found to increase over large parts of the globe due to the combined effect of increasing mean temperatures and positive trends in surface solar radiation projected by the CMIP5 models [32]. In the study of Zou et al.[33], increasing trends of PV power (modeled as function of temperature) were found in East Asia, Europe, Central Africa, the northern part of South America, Central America, and central and eastern China in the 2006-2100 period. Such a trend was attributed to a decreasing trend in the atmospheric aerosols burden. For other regions such as North Africa, Central Asia, Australia, and especially the Tibetan Plateau, decreasing trends of PV power were found, associated with increasing aerosol burdens and cloudiness in the cases of the North Africa and the Tibetan Plateau regions.

Our examination of the literature revealed only one study (de Jong et al.[34]) focusing on a LAC domain. This study employed a downscaled regional climate model forced by 3 CMIP5 GCMs under RCP8.5, and projected a slight increase in average incoming surface solar radiation across most of Brazil. Particularly in the northeastern Brazil (where most of solar and wind power capacities have been concentrated on), the projected increase is of about 3.6% by the 2080s relative to the 1970s.

**Wind**

The theoretical extractable power of wind is directly proportional to the near-surface wind speed to the third power and air density [35]. Hence, the inherent physical relationship involving the general atmospheric circulation, surface pressure and temperature gradients render large to local-scale wind potential sensitive to global warming, resulting in alterations in the mean and extreme wind speeds, direction as well as in the resource variability across different temporal scales [36,37]. While changing annual and seasonal mean wind speeds affect power-generation capacity, changes in the magnitude and frequency of extreme wind speeds can affect farms infrastructure through damages to the turbines.

A recent global-scale study by Karnauskas et al.[38] based on an ensemble of ten GCMs from CMIP5 under RCPs 4.5 and 8.5 portrayed decreases in wind power across the Northern Hemisphere mid-latitudes and increases across the tropics and Southern Hemisphere (in this case with substantial regional variations and larger sensitivity to the emissions scenario). These responses were attributed to the polar amplification in the northern mid-latitudes, whereas

enhanced land–sea thermal gradients governed the tropical and southern subtropical increases. By investigating some focal regional domains in more detail, the study revealed increasing wind power over time for subdomains covering the Mexico-Caribbean and eastern Brazil regions and an opposite pattern in southern South America under the RCP8.5.

Narrowing down to regional impact studies in Latin America, the existing literature has focused in Brazil. Pereira de Lucena et al. and Pereira et al.[39,40] employed projections from the HadCM3 GCM (dynamically downscaled into regional climate projections) under high and low IPCC SRES emissions scenarios, showing growth in resource availability in most regions over the long term, particularly in the Northeast region of Brazil. From both studies, the most conservative is Pereira et al.[40], in which the average future growth in the wind power density inland for most of Northeast falls within the 15–30% range relative to the 1962-1990 baseline. These earlier findings agree with recent results produced by de Jong et al.[34] and Ruffato-Ferreira et al.[20], where wind speeds are projected to increase across most of Brazil. By applying trend analysis techniques on a 50-year historical series of wind speed data, Pes et al.[41] identified a positive trend for maximum wind speed in most stations analyzed, particularly in the mid-latitudes portions of Brazil.

**Supplementary Note 2. Overview of the Global Change Analysis Model (GCAM)**

This section provides an overview of basic aspects of the Global Change Analysis Model (GCAM) version 5.1, and consists of a summary of information available in the GCAM documentation (available at https://jgcri.github.io/gcam-doc/v5.1/toc.html), Calvin et al.[1] and Santos da Silva et al.[42], with other supporting references highlighted throughout the following text.

GCAM is a state-of-the-art integrated assessment model (IAM) developed by the Joint Global Change Research Institute of the Pacific Northwest National Laboratory. As an open source model, current and prior releases are available at https://github.com/JGCRI/gcam-core/releases. In GCAM, key interactions between population, economic growth, energy, land, water, and climate systems are linked within a consistent economic modeling framework, allowing the assessment of a range of questions aimed at informing decision-making. GCAM has a long tradition in supporting climate science, having been employed, for example, to model the representative concentration pathway (RCP) 4.5 [43] and the Shared Socioeconomic Pathway (SSP) 4 storyline [44].

The fundamental trigger of sectors demands within the model is the human system (i.e., population and gross domestic product (GDP) growth assumptions), which drives the future evolution of energy, water and land sectors. On top of socioeconomic pathways, a range of mitigation policies, climate impact inputs, adaptation strategies, technological options in distinct sectors, among other assumptions can be added within the scenarios set-up. This allow a multi-sectoral assessment of implications in a way that the model solution represents the least-cost and most technically feasible combination of existing technologies and resources quantities and quality per region.

GCAM is a five-year step, long-term, market-equilibrium model with global coverage. The base year for the model is 2010 based on calibration to the historical period, which requires multiple datasets (listed in Calvin et al.[1] as well as in the GCAM documentation) to cover the different sectors. In terms of solution algorithm, GCAM is a dynamic-recursive model, which solves each period sequentially (based on existing information for the period being solved) through the establishment of market-clearing prices for all existing markets (energy, agriculture, land, GHG emissions). This means that, for each model period, an iterative scheme ensures convergence to final equilibrium prices such that supplies and demands are equal in all markets.

The energy system contains representations of the energy supply and demand sectors for each region, also considering the trading of primary resources (coal, natural gas, oil and biomass) among regions. The model simulates the temporal evolution of the energy system from the extraction of primary energy resources (oil, natural gas, coal, bioenergy, uranium, hydropower, geothermal, solar, and wind energy) until the transformation processes (e.g., liquid fuel refineries and power generation) that produce the final energy carriers (refined liquids, gas, coal, commercial bioenergy, hydrogen, and electricity) required by the end-use sectors (buildings, industry, and transport). All energy-sector prices are calculated endogenously. GCAM utilizes a comprehensive technology database that includes more than 100 different energy supply and

conversion technology representations and includes assumptions regarding technological progress[45].

The GCAM representation of capital stock turnover in the electric power sector assumes that generating technologies have a prescribed lifetime, and investments in new plants are added by vintage (i.e., period in which the investment is made) in a pace that allows sufficient generating capacity exists to meet demand. Each power plant operates until the end of its lifetime or is retired from production if its operating costs surpass the electricity market price. The new technology investments compete for a share of energy markets. Such a competition is represented by the logit-choice formulation discussed in the main text (Methods).

The agriculture and land-use system provides projections of agricultural supply (crops, livestock, forestry and bioenergy products), commodity prices, and changes in land use and cover in each region, and accounts for the trading of primary agricultural and forest goods in a way that global supplies equilibrates demands at each time step. The spatial resolution in this submodule is determined by the intersection of the 32 (33 in the GCAM-LAC version as Uruguay is a distinct region) geopolitical regions with the 235 global river basins represented in GCAM. Within each land-use region, land is categorized according to distinct land use (e.g., croplands, pasture, commercial forest and urban) and land cover types (e.g., grasslands, shrubs, non-commercial forest, etc.), with the supply of agricultural products depending on the amount of land dedicated to the specific good and its yield. Land used for any purpose competes economically with croplands, commercial forests, pastures, and all lands not involved in commodity production, with the exception of tundra, deserts, and urban lands (assumed constant over time). Land allocation decisions are based on the expected profitability of each land type, implemented via a logit function that allocates larger shares where profit rates are expected to be the largest, but leaves some share to less profitable land categories. The profitability of any land used for commercial production is derived from the price of the commodity produced, the costs of production, and the yield[46]. GCAM models the production of 15 crop categories relying on exogenous assumptions of agricultural yield improvements over time that aim at capturing technological progress (e.g., better farming practices, seed variety advances, etc.). Unique assumptions apply to each individual land unit of the model. Moreover, yields can be endogenously intensified (or even decline) over time depending on the crop management options deployed throughout the simulation. Presently, there exists four management options (irrigated/high fertilizer, irrigated/low fertilizer, rainfed/high fertilizer, and rainfed/low fertilizer), each with a different yield and cost of production for each crop. The endogenous decision upon the yield intensification (or decline) is also based on the expected profitability of each management practice, which determines its share within the land unit.

Bioenergy production (in which demands are determined by the energy system) derives from: (1) various types of second-generation cellulosic crops (e.g., switchgrass, miscanthus, willow, jatropha, and eucalyptus), (2) residues from forestry and agriculture, (3) municipal solid waste, and (4) traditional bioenergy. Conventional or first-generation biofuel crops such as corn, sugars, oil crops are grown as part of the food crop production. In this case, the biomass liquids subsector within the energy module includes a number of transforming technologies for biofuels production from these first-generation crops.

The physical atmosphere, oceans and climate are represented in GCAM by the Hector Earth System model[47], which is a reduced-form global climate carbon-cycle model (or simple climate model–SCM). As a SCM, Hector was developed to represent only the most important large-scale earth system processes so that to significantly reduce computational costs relative to the most complex Earth-System Models. Although it can be used as a standalone model, Hector is fully integrated within the computational GCAM platform. This coupling allows Hector to track emissions of 24 GHGs and short-lived species generated by the energy, agriculture and land systems and to calculate future GHG concentrations in each modeling scenario. From GHG concentrations and short-lived climate forcers, Hector can then derive global mean radiative forcing, which is converted to global mean temperature and other variables. It is important to note that there is no feedback from Hector on any of the GCAM sectors based on the levels of radiative forcing or global mean temperature achieved from a given emission pathway.

The water module within GCAM provides water demand estimates (withdrawals and consumption) for six sectors: irrigation, livestock, primary energy production and processing, electricity generation, industrial, and municipal. The main characteristics of the GCAM water module are: (1) water demand for irrigation depends on the share of irrigated land and the water coefficient (water demand per unit output), which varies by crop and region; (2) livestock demand depends on a region-specific coefficient which represents both animal drinking water, plus any other water used by the animal production operations; (3) for electricity water demands, GCAM has exogenously assigned water withdrawal and consumption coefficients for each region and generation technology; (4) the industrial sector's water demands scale with industrial output while domestic water demands are driven by socioeconomic assumptions; and (5) demands for primary energy production depends on fuel production and the estimates of water demand per unit energy produced for a range of fuels.

The results presented in this study were generated through a research version of GCAM best suited for analyses in LAC (GCAM-LAC)[26] as noted in the main text.

## Supplementary Note 3. GCAM representation of climate change impacts on renewables

To account for the compounding climate-induced effects on LAC's power sector, we force GCAM with representations of changing agricultural productivity and hydropower production as well as with climate-impacted solar and wind cost-supply curves. These model inputs are predicated on bias-corrected projections from the GFDL-ESM2M, HadGEM2-ES and IPSL-CM5A-LR general circulation models (GCMs) obtained from the Inter-Sectoral Impact Model Intercomparison Project (ISIMIP)[48,49] under the representative concentration pathways 2.6 (RCP2.6) and 6.0 (RCP6.0). Next, the specific methods behind each generation technology are outlined.

The GCAM agricultural/land system requires information on yield changes at each time step in the 384 land units covering the globe (note that a land unit is formed by the intersection of geopolitical regions and water basins). While yields in the historical calibrated years are computed from a combination of datasets (listed in Calvin et al.[50]), changes in yields over time are computed from the Food and Agriculture Organization projections[46,50]. These yield changes constitute the GCAM baseline assumptions, which do not account for the effects of climate change. These are fixed assumptions per GCAM land unit that generally improve over time due to technological progress (e.g., better farming practices, seed variety advances, etc.)[51]. Moreover, these exogenous baseline yields can be modified during a GCAM simulation due to endogenous shifts in management practices (irrigation and fertilizing).

To incorporate climate change impacts, global gridded yields simulated by the parallel Decision Support System for Agrotechnology Transfer (pDSSAT - the parallelized global gridded version of the DSSAT model[52,53]) provided the basis to modify the baseline GCAM yield change assumptions. This cropping system simulates changes in the response of a crop to environmental conditions under different crop management options, accounting for water, temperature, specific-heat, nitrogen, and oxygen stresses as well as $CO_2$ fertilization effects[11].

Applying yield information from pDSSAT into GCAM requires some data processing to accommodate differences in spatial, temporal, and commodity resolutions between pDSSAT and GCAM. These steps are described in Calvin et al.[50], and can be summarized as follows. The pDSSAT dataset comprises gridded (spatial resolution of $0.5^{o} \times 0.5^{o}$) annual yield information for both irrigated and rain-fed crops. The latter allowed climate-induced yield changes to be applied separately in GCAM rain-fed and irrigated crops. These gridded yields are aggregated (separately for each crop-irrigation pair) to the GCAM land units using MIRCA2000[54] harvested area data for weighting. As the collections of crops represented by pDSSAT differ from the commodities modeled in GCAM, it is necessary to map each GCAM commodity to a pDSSAT crop type (see specifically section S1.1 in Calvin et al.[50] for details on this mapping). In the specific case of the second-generation bioenergy crops (such as switchgrass, miscanthus, etc.), which are not represented by pDSSAT, GCAM's biomass crop commodity receives the median of impacts to all other commodities. At the land unit scale, the pDSSAT time series of yields are converted to relative changes in yield from 2010. Specifically, before being applied to GCAM's baseline yield change assumptions, the pDSSAT time series of yields are (1) converted to multipliers by dividing by the historical baseline average yield for each crop-irrigation-land unit combination, and (2) smoothed using 30-yr moving averages. This method for incorporating

climate driven yield changes as multipliers on GCAM's exogenous yield assumptions follows methods used in the broader literature[46,55-57]. The outcome of this data processing is a modified model input of agricultural productivity change (written in the form of percent changes relative to the previous model time step). The pDSSAT simulations used in this study are part of the Agricultural Model Inter-comparison Project (AGMIP)[11] following RCPs 2.6 and 6.0, and were taken from the experiments that included $CO_2$ effects.

The effect of the climate-impacted yield change assumptions is to modify land profit rates across land units in the model. Note that GCAM uses this information on expected profitability to determine land allocated to each land type (cropland, biomass, grassland, shrubland, pasture, forest, etc.), and that the combination of yields and endogenous land allocation determines agricultural production in each land unit at each time step.

On the supply side of the energy sector in GCAM, "no-climate impacts" baseline hydropower assumptions (derived from the economic and technical potentials estimated by the International Hydropower Association[1]) are read in at the start of a simulation. Specifically, the model reads in predetermined quantities of hydroelectricity production (in EJ) for all time-steps and GCAM regions. These prescribed input quantities are then used by the model to determine the temporal evolution of hydropower production by GCAM region. This means that hydropower production does not result from the modeled economic competition like all other power-sector technologies represented in GCAM.

To incorporate gains/losses in hydropower production under evolving climatic conditions, the above-mentioned baseline hydropower assumptions are modified using data from the GHM Xanthos [58]. Xanthos supplies GCAM with information regarding water availability in the 235 large river basins represented in GCAM, as well as hydropower production. Xanthos evolved from the Global Water Availability Model [59], and currently includes four different methods to compute potential evapotranspiration (PET) as well as the abcd method [60] as the default runoff module. Xanthos requires gridded monthly precipitation and temperature and solves for monthly runoff and other variables at grid-cell level, relying on the modified River Transport Model (described in Zhou et al.[61]) as the routing scheme to produce streamflow data. Apart from its core components (PET, runoff generation, and river routing), Xanthos 2.0 features a hydropower module (based on Turner et al.[23]) that requires gridded streamflow projections (converted from the simulated runoff) to drive dam simulations. This module includes a dam database of existing and planned dams with information on the associated reservoir and plant properties, and assigs a turbine release for each dam at each time step. Data processing included aggregation from grid cells to GCAM regions and from monthly to yearly resolution, as well as the smoothing of the resulting yearly hydropower generation pathway to remove inter-annual variability. Lastly, for each GCAM region, absolute hydroelectricity generation (in energy units) in all future years are converted into percent changes relative to 2010 as in Turner et al.[23]. The latter is superimposed onto the baseline GCAM hydropower generation quantities producing a modified hydropower production pathway that incorporates climate change effects. Note that the Xanthos runs for this study were conducted with the Penman-Monteith method to estimate PET.

Climate-impacted solar and wind inputs comprise resource cost-supply curves (i.e., curves that map the availability of the resource at different energy production cost levels) built

upon the technical and economic potentials produced by Gernaat 2019 [62] using ISIMIP2b climate forcings. The methodology described by Gernaat 2019 [62] for the derivation of the solar and wind potentials that served as basis for the supply curves implemented in GCAM comprises the assessment of four general categories of "potentials" [63-65]: (1) theoretical potential – total primary energy content associated with each RE source; (2) geographical potential – the theoretical potential in suitable and available areas of the globe; (3) technical potential – the geographical potential after taking into consideration efficiency losses of the conversion from the primary resource to secondary energy forms (i.e., electricity or fuels); and (4) economic potential – the technical potential that can be realized economically given the estimated production cost of the secondary energy carrier.

While the GCMs simulations provide the spatial distribution and temporal evolution of the primary natural resource at a grid-cell level (0.5$^{o}$ spatial resolution), the geographic potential entails explicit consideration of geospatial constraints that limit the theoretical primary potential. Specifically, a set of exclusion criteria based on geospatial data (e.g., exclusion of urban areas, protected land, elevated terrain, forests, areas below a minimum resource availability threshold, among others) were used to estimate the amount of land suitable and available for RE production. This is followed by an assessment of constraints posed by technical and/or operational factors (e.g., efficiency of PV panels and CSP modules, wind turbine characteristics, density of installations, turbine availability in a year, wind array efficiency, etc.) that accounts for the fundamental premise that only a portion of the geographical potential can be converted into secondary energy. This step results in the technical potential. Finally, the production costs of electricity need to be estimated given that renewables must compete for a share of regional energy markets with other sources. This results in the economic potential, which includes considerations of capital and operational costs that are expressed in terms of a parameter known as "cost of energy" (COE). Full details and assumptions are described in Gernaat 2019 [62].

To produce supply curves for all GCAM regions, we arranged the technical potential data across the grid cells corresponding to each GCAM region in order of ascending electricity costs (given by the economic potential maps) considering all generating sources, time-periods and GCMs. This led to the derivation of three time-varying supply curves per renewable source and GCM (Supplementary Figs. 17 – 28), which we utilized to replace GCAM default assumptions that do not consider climate change effects on the solar and wind primary resource production. In the case of wind, the default supply curves derive from a reanalysis dataset covering the 1980-2009 period[66]. Solar energy is modeled as two separate resources: global solar resource and distributed PV (accounting for PV installations on residential and commercial buildings)[67]. While the GCAM default framework models the distributed PV resource with supply curves derived from an observational solar radiation dataset[68], no cost-supply curve is implemented for the global solar primary resource, which is assumed an unlimited resource with a very low price[67].

Replacing default GCAM assumptions by the modified supply curves implemented in this study has important implications. In GCAM, primary renewable resource production and their marginal resource-related costs serve as inputs to the energy transformation sectors such as the electricity sector, which contains representations of distinct generating technologies (fossil fuels, geothermal, hydropower, intermittent renewables, nuclear). These generating technologies

compete for a share of regional electricity markets on the basis of their relative costs. This competition is modeled by a logit-choice formulation as mentioned in the main text and in the Supplementary Note 2 (note that hydropower is set aside from economic competition since hydropower production is a fixed input to the model). Hence, the climate-impacted renewable supply curves affect both the availability of the renewable resource and the marginal costs of producing electricity from the resource, which is key to determine how much market share each generating technology captures.

**Supplementary Note 4. Detailed description of the emissions pathway and additional assumptions behind scenarios explored in this study**

We first devised the emission reduction scenario, named *NDC_to_2C*, to serve as the basis for all mitigation scenarios – with and without climate change impacts – in this study. In the *NDC_to_2C* scenario, 2020-2030 regional emissions pathways represent commitments made by United Nations Framework Convention on Climate Change's parties in the Paris Agreement. The quantification of the NDCs in Latin America and the Caribbean (LAC) within the 2020-2030 period follows Vrontisi et al.[5] (Supplementary Tables 6 and 7), which represents the conditional (high-ambition) NDCs by establishing 2025 or 2030 emissions changes relative to 2010 levels. Outside the LAC region, GHG emissions reductions are represented as single emissions constraints that are shared among the remainder of the world.

Beyond 2030 (period not covered by the current round of NDCs), it is assumed that all regions worldwide enhance their mitigation efforts resulting in the mean global surface temperature increase successfully limited to 2°C. This is achieved with all regions complying with annual rates of improvement in GHG emissions (excluding $CO_2$ emissions from land-use and land-cover change – LUC) per unit of GDP (i.e., GHG intensity) of 4.5%. Weaker GHG intensities were unable to meet the end-of-century 2°C climate goal. Note that $CO_2$ LUC emissions were not included in the GHG intensity computation due to the large uncertainty on the actual role of the land sector in future mitigation as discussed in the literature[69,70]. In the GCAM framework, GHG emissions in the *NDC_to_2C* scenario are limited by imposing economy-wide emissions constraints (computed from the GHG intensity values calculated above). This means that total GHG emissions are assigned to each GCAM region and the model internally calculates the carbon prices needed to achieve the emissions constraint. Following Binsted et al.[71], we assume medium mitigation efforts in the land-use sector globally by imposing that $CO_2$ emissions from LUC face a price that is 10% of the price per ton of carbon on fossil fuel and industrial emissions. Reductions in non-$CO_2$ emissions are achieved through equal marginal abatement costs across all sectors of the economy. It is important to acknowledge that actual climate policy approaches will significantly differ from the economy-wide carbon prices approach used herein, relying, for example, on a range of different sectoral measures. Hence, the resulting emissions pathway is meant to be illustrative. Indeed this scenario is only one of many possible scenarios that might reach the 2°C goal.

By designing a mitigation scenario in line with the 2°C climate goal, we aim at keeping consistency with the global warming level associated with our set of climate impact inputs developed under the RCP2.6 (Supplementary Note 3). As noted in the main text, the RCP2.6 provides climate forcing levels consistent with the long-term goal of the Paris Agreement of keeping global warming likely below 2°C above pre-industrial temperatures[4]. Supplementary

Figs. 49 and 50 compare the *NDC_to_2C* scenario with a range of policy scenarios consistent with the RCP2.6 forcing levels explored by the scientific literature (for comparison, the GCAM baseline, i.e., "no policy" scenario is also shown; except for the climate policy component, the GCAM baseline and *NDC_to_2C* scenarios share all other assumptions).

Global population and GDP assumptions in the *NDC_to_2C* derive from the GCAM implementation of the "Middle of the Road" Shared Socioeconomic Pathway (SSP) 2, which reflects a world in which social, economic and technological future trends do not differ markedly from historical patterns[2]. However, as noted in the Supplementary Note 2, socioeconomic assumptions in LAC (specifically in Argentina, Colombia and Uruguay) were revised to align with LAC countries' future projections.

All mitigation scenarios explored in this study are based on the *NDC_to_2C* scenario. In other words, all *RCP26* scenarios presented in Table 1 of the main text share the same assumptions with the *NDC_to_2C* scenario, except for the climate impacts and technology availability components. However, note that the *NDC_to_2C* and the *RCP26_FullTech: No-Climate impacts* scenarios share exactly the same assumptions since the *NDC_to_2C* scenario were constructed assuming that the full suite of power sector technologies represented by GCAM was available globally and under the assumption of no climate impacts on renewables.

**Supplementary Note 5. Calculation of capital investments in electric power sector**

Power-sector capital investments were calculated as in Iyer et al.[72] using GCAM outputs of electricity generation (EJ) by technology, vintage (i.e., the period in which the investment is made) and period. The first step is to compute new and additional electricity generation for each technology in each period, which is converted to capacity (in GW) via the capacity factor assumptions listed in the Supplementary Table 8 and 9. This can be expressed as:

$$\text{Capacity} = \frac{\text{Generation} \times (2.78 \times 10^5)}{\text{Capacity Factor} \times 8760} \qquad \text{(GW)}$$

Finally, the capacity addition calculated above is multiplied by the overnight capital cost associated with each technology (in $ per kilowatt) using assumptions listed in the Supplementary Table 10. This yields capital investments (in $ – representative of cumulative investments over a five-year model period) as shown below:

$$\text{Capital investment} = \text{Capacity} \times 10^6 \times \text{Overnight Capital Costs} \qquad (\$)$$

Note that GCAM does not track hydroelectric power plant capital by vintage as in the case of other generating technologies since future hydroelectricity generation is set exogenously for each model region. In this case, GCAM outcomes of hydropower generation by period were used to compute the additional power generation between model periods (i.e., by calculating differences in electricity generation between periods). Then, this additional power generation was converted into capacity addition and capital investments using the equations listed above. It is important to mention that capital investments computed by the method outlined here represent the upfront costs that occur at the beginning of the lifetime of a power station. Variable costs

(e.g., fuel costs and operation and maintenance costs) and other system costs (e.g., integration) are not included.

## Supplementary Note 6. Ancillary climate-impact model simulations

As noted in the main text, we have performed ancillary experiments based on the technology scenarios defined in the main text but assuming climate impacts on each renewable individually (similar to the approach conducted by Turner et al.[23] for hydropower and by Kyle et al.[46] for agricultural yields). Specifically, we have carried out simulations where each climate-impact input is incorporated into GCAM individually, and have assessed implications on electricity generation per source considered (i.e., if climate impacts on wind were incorporated in GCAM, changes in wind power generation were examined and so on). This is intended to help the understanding of the major driving forces acting on LAC's decarbonizing power sector under multiple simultaneous climate impacts (i.e., under the *Combined impacts* assumption).

By incorporating the physical impacts of climate change on the RE supply into our energy-economic framework, concurrent direct and indirect effects are induced. The former means the direct power-system responses to the climate-impacted RE inputs such as a decline in hydroelectricity production due to reduced streamflow volumes or an increase in bioenergy production due to improved crop yields. The indirect responses derive from feedbacks of the direct effects on the power system such as changes in the economic competitiveness of generating technologies relative to others, which play a key role in driving energy-technology decisions. More complex interactions emerge under multiple simultaneous climate impacts. This is illustrated in Supplementary Figs. 11–14, which show the model mean changes in cumulative renewable electricity production across LAC for the 2020–2100 and 2020–2050 periods. When climate impacts on individual renewables are assumed (left panels), magnitudes and signs of the resulting direct changes in renewable electricity generation vary considerably across LAC with effects on hydropower- and wind-based generation outweighing those on biomass and solar generation (see also Supplementary Tables 2 – 3 for the specific numerical values). When all impacts are jointly accounted for in the *Combined impacts* scenarios (defined in the main text), bioenergy and solar generation undergo more pronounced variations responding to the compounding indirect effects (price and demand adjusts) driven mostly by hydropower and wind sources ( Supplementary Figs. 11–14; center and right panels).

## Supplementary Note 7. Representation of renewable energy intermittency in GCAM

As noted in the main text, GCAM includes a representation of renewable intermittency. Like most IAMs, this is translated into costs that vary with share of renewables in the grid. As explained in the main, long-term changes in RE potentials induced by climate change affect changes in supply curves. This process, in turn, affects the amount of renewables in the grid and hence the intermittency costs computed by GCAM.

The calculation of intermittency costs is as follows. For each of the solar and wind technologies, GCAM includes, both a purely intermittent option and an option paired with dedicated storage. The representation of intermittency costs for intermittent renewables attempts to reflect the diminishing contributions to electric capacity reserves as the share of intermittent technologies in the grid increases. This is done with a simple exponential functional form that adds to the cost of adding new intermittent generation to secure additional capacity. The

marginal reserve capacity requirement is computed as an exponential function of the share of intermittent renewable capacity in total electric power capacity as follows:

$$\text{CapacityRatio} = \frac{F_{max}}{1 + \exp\left(C * \frac{(x_{mid} - \text{elecShare})}{\tau}\right)}$$

where CapacityRatio is the ratio of the reserve capacity to the capacity of intermittent renewable, $F_{max}$ is the maximum capacity ratio (defaults to 1), $C$ and $\tau$ are shape parameters (ratio of $C$ and $\tau$ determine the steepness to reach $F_{max}$), elecShare is the share of total intermittent renewable capacity in total power capacity, $x_{mid}$ is the value of elecshare at which CapacityRatio = 0.5* $F_{max}$.

A representative curve with $F_{max}$ =1; $C$ =5 and $\tau$ =0.1 and $x_{mid}$ = 0.35 is shown in Supplementary Figure 51 below.

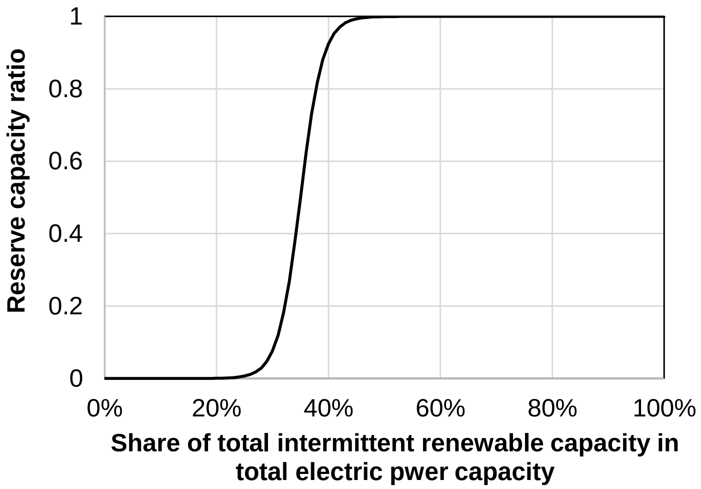


**Supplementary Figure 51.** Reserve capacity ratio as a function of share of intermittent renewable capacity in total electric power capacity.

At low shares of intermittent technologies, each additional intermittent unit requires a small fraction of reserve capacity. At a total share of 35%, the marginal intermittent unit counts 50% toward capacity reserves. The cost of reserve capacity requirements is then calculated by assuming that the reserve capacity is provided by natural gas combustion turbines operating at a capacity factor of 5%.

## Supplementary References


1. Calvin, K. *et al.* GCAM v5. 1: representing the linkages between energy, water, land, climate, and economic systems. *Geoscientific Model Development* **12**, 677-698 (2019).
2. Riahi, K. *et al.* The Shared Socioeconomic Pathways and their energy, land use, and greenhouse gas emissions implications: An overview. *Global Environmental Change* **42**, 153-168, doi:https://doi.org/10.1016/j.gloenvcha.2016.05.009 (2017).
3. Krey, V. *et al.* Annex II: Metrics & Methodology. In: Climate Change 2014: Mitigation of Climate Change. Contribution of Working Group III to the Fifth Assessment Report of the Intergovernmental Panel on Climate Change. (Cambridge University Press, Cambridge, United Kingdom and New York, NY, USA, 2014).
4. IPCC. Climate Change 2014: Synthesis Report. (IPCC, Geneva, Switzerland, 2014).
5. Vrontisi, Z. *et al.* Enhancing global climate policy ambition towards a 1.5 °C stabilization: a short-term multi-model assessment. *Environmental Research Letters* **13**, 044039, doi:10.1088/1748-9326/aab53e (2018).
6. Riahi, K. *et al.* Locked into Copenhagen pledges — Implications of short-term emission targets for the cost and feasibility of long-term climate goals. *Technological Forecasting and Social Change* **90**, 8-23, doi:https://doi.org/10.1016/j.techfore.2013.09.016 (2015).
7. Schaeffer, R. *et al.* Energy sector vulnerability to climate change: A review. *Energy* **38**, 1-12, doi:https://doi.org/10.1016/j.energy.2011.11.056 (2012).
8. Cronin, J., Anandarajah, G. & Dessens, O. Climate change impacts on the energy system: a review of trends and gaps. *Climatic Change* **151**, 79-93, doi:10.1007/s10584-018-2265-4 (2018).
9. Solaun, K. & Cerdá, E. Climate change impacts on renewable energy generation. A review of quantitative projections. *Renewable and Sustainable Energy Reviews* **116**, 109415, doi:https://doi.org/10.1016/j.rser.2019.109415 (2019).
10. IPCC. Special Report on Renewable Energy Sources and Climate Change Mitigation. (Cambridge University Press, New York, NY, 2012).
11. Rosenzweig, C. *et al.* Assessing agricultural risks of climate change in the 21st century in a global gridded crop model intercomparison. *Proceedings of the National Academy of Sciences* **111**, 3268, doi:10.1073/pnas.1222463110 (2014).
12. Tubiello, F. N., Soussana, J.-F. & Howden, S. M. Crop and pasture response to climate change. *Proceedings of the National Academy of Sciences* **104**, 19686, doi:10.1073/pnas.0701728104 (2007).
13. Magrin, G. O. *et al.* in *Climate Change 2014: Impacts, Adaptation, and Vulnerability. Part B: Regional Aspects. Contribution of Working Group II to the Fifth Assessment Report of the Intergovernmental Panel on Climate Change* (eds V.R. Barros *et al.*) (Cambridge University Press, 2014).
14. Marin, F. R. *et al.* Climate change impacts on sugarcane attainable yield in southern Brazil. *Climatic Change* **117**, 227-239, doi:10.1007/s10584-012-0561-y (2013).
15. Carvalho, A. L. d. *et al.* Impact of climate changes on potential sugarcane yield in Pernambuco, northeastern region of Brazil. *Renewable Energy* **78**, 26-34, doi:https://doi.org/10.1016/j.renene.2014.12.023 (2015).
16. Rolla, A. L. *et al.* Climate impacts on crop yields in Central Argentina. Adaptation strategies. *Agricultural Systems* **160**, 44-59, doi:https://doi.org/10.1016/j.agsy.2017.08.007 (2018).
17. Arent, D. J. *et al.* in *Climate Change 2014: Impacts, Adaptation, and Vulnerability. Part A: Global and Sectoral Aspects. Contribution of Working Group II to the Fifth Assessment Report of the Intergovernmental Panel on Climate Change* (eds C.B. Field *et al.*) 659–708 (Cambridge University Press, 2014).

18 van Vliet, M. T. H. *et al.* Multi-model assessment of global hydropower and cooling water discharge potential under climate change. *Global Environmental Change* **40**, 156-170, doi:https://doi.org/10.1016/j.gloenvcha.2016.07.007 (2016).

19 Hamududu, B. & Killingtveit, A. Assessing Climate Change Impacts on Global Hydropower. *Energies* **5**, doi:10.3390/en5020305 (2012).

20 Ruffato-Ferreira, V. *et al.* A foundation for the strategic long-term planning of the renewable energy sector in Brazil: Hydroelectricity and wind energy in the face of climate change scenarios. *Renewable and Sustainable Energy Reviews* **72**, 1124-1137, doi:https://doi.org/10.1016/j.rser.2016.10.020 (2017).

21 De Souza Dias, V., Pereira da Luz, M., Medero, M. G. & Tarley Ferreira Nascimento, D. An Overview of Hydropower Reservoirs in Brazil: Current Situation, Future Perspectives and Impacts of Climate Change. *Water* **10**, doi:10.3390/w10050592 (2018).

22 Almeida Prado, F. *et al.* How much is enough? An integrated examination of energy security, economic growth and climate change related to hydropower expansion in Brazil. *Renewable and Sustainable Energy Reviews* **53**, 1132-1136, doi:https://doi.org/10.1016/j.rser.2015.09.050 (2016).

23 Turner, S. W. D., Hejazi, M., Kim, S. H., Clarke, L. & Edmonds, J. Climate impacts on hydropower and consequences for global electricity supply investment needs. *Energy* **141**, 2081-2090, doi:https://doi.org/10.1016/j.energy.2017.11.089 (2017).

24 Lucena, A. F. P. *et al.* Interactions between climate change mitigation and adaptation: The case of hydropower in Brazil. *Energy* **164**, 1161-1177, doi:https://doi.org/10.1016/j.energy.2018.09.005 (2018).

25 Arango-Aramburo, S. *et al.* Climate impacts on hydropower in Colombia: A multi-model assessment of power sector adaptation pathways. *Energy Policy* **128**, 179-188, doi:https://doi.org/10.1016/j.enpol.2018.12.057 (2019).

26 Zarrar, K. *et al.* Integrated energy-water-land nexus planning to guide national policy: an example from Uruguay. *Environmental Research Letters* (2020).

27 Collins, M. *et al.* in *Climate Change 2013: The Physical Science Basis. Contribution of Working Group I to the Fifth Assessment Report of the Intergovernmental Panel on Climate Change* (eds T.F. Stocker *et al.*) (Cambridge University Press, 2013).

28 Mavromatakis, F. *et al.* Modeling the photovoltaic potential of a site. *Renewable Energy* **35**, 1387-1390, doi:https://doi.org/10.1016/j.renene.2009.11.010 (2010).

29 Crook, J. A., Jones, L. A., Forster, P. M. & Crook, R. Climate change impacts on future photovoltaic and concentrated solar power energy output. *Energy & Environmental Science* **4**, 3101-3109, doi:10.1039/C1EE01495A (2011).

30 Bergin, M. H., Ghoroi, C., Dixit, D., Schauer, J. J. & Shindell, D. T. Large Reductions in Solar Energy Production Due to Dust and Particulate Air Pollution. *Environmental Science & Technology Letters* **4**, 339-344, doi:10.1021/acs.estlett.7b00197 (2017).

31 Wild, M., Folini, D., Henschel, F., Fischer, N. & Müller, B. Projections of long-term changes in solar radiation based on CMIP5 climate models and their influence on energy yields of photovoltaic systems. *Solar Energy* **116**, 12-24, doi:https://doi.org/10.1016/j.solener.2015.03.039 (2015).

32 Wild, M., Folini, D. & Henschel, F. Impact of climate change on future concentrated solar power (CSP) production. *AIP Conference Proceedings* **1810**, 100007, doi:10.1063/1.4975562 (2017).

33 Zou, L. *et al.* Global surface solar radiation and photovoltaic power from Coupled Model Intercomparison Project Phase 5 climate models. *Journal of Cleaner Production* **224**, 304-324, doi:https://doi.org/10.1016/j.jclepro.2019.03.268 (2019).

34 de Jong, P. *et al.* Estimating the impact of climate change on wind and solar energy in Brazil using a South American regional climate model. *Renewable Energy* **141**, 390-401, doi:https://doi.org/10.1016/j.renene.2019.03.086 (2019).
35 Eurek, K. *et al.* An improved global wind resource estimate for integrated assessment models. *Energy Economics* **64**, 552-567, doi:https://doi.org/10.1016/j.eneco.2016.11.015 (2017).
36 Pryor, S. C. & Barthelmie, R. J. Climate change impacts on wind energy: A review. *Renewable and Sustainable Energy Reviews* **14**, 430-437, doi:https://doi.org/10.1016/j.rser.2009.07.028 (2010).
37 Pryor, S. C. & Barthelmie, R. J. Assessing the vulnerability of wind energy to climate change and extreme events. *Climatic Change* **121**, 79-91, doi:10.1007/s10584-013-0889-y (2013).
38 Karnauskas, K. B., Lundquist, J. K. & Zhang, L. Southward shift of the global wind energy resource under high carbon dioxide emissions. *Nature Geoscience* **11**, 38-43, doi:10.1038/s41561-017-0029-9 (2018).
39 Pereira de Lucena, A. F., Szklo, A. S., Schaeffer, R. & Dutra, R. M. The vulnerability of wind power to climate change in Brazil. *Renewable Energy* **35**, 904-912, doi:https://doi.org/10.1016/j.renene.2009.10.022 (2010).
40 Pereira, E. B., Martins, F. R., Pes, M. P., da Cruz Segundo, E. I. & Lyra, A. d. A. The impacts of global climate changes on the wind power density in Brazil. *Renewable Energy* **49**, 107-110, doi:https://doi.org/10.1016/j.renene.2012.01.053 (2013).
41 Pes, M. P. *et al.* Climate trends on the extreme winds in Brazil. *Renewable Energy* **109**, 110-120, doi:https://doi.org/10.1016/j.renene.2016.12.101 (2017).
42 Santos Da Silva, S. R. *et al.* The Paris pledges and the energy-water-land nexus in Latin America: Exploring implications of greenhouse gas emission reductions. *PLOS ONE* **14**, e0215013, doi:10.1371/journal.pone.0215013 (2019).
43 Thomson, A. M. *et al.* RCP4.5: a pathway for stabilization of radiative forcing by 2100. *Climatic Change* **109**, 77, doi:10.1007/s10584-011-0151-4 (2011).
44 Calvin, K. *et al.* The SSP4: A world of deepening inequality. *Global Environmental Change* **42**, 284-296, doi:https://doi.org/10.1016/j.gloenvcha.2016.06.010 (2017).
45 McJeon, H. *et al.* Limited impact on decadal-scale climate change from increased use of natural gas. *Nature* **514**, 482-485, doi:10.1038/nature13837 (2014).
46 Kyle, P., Müller, C., Calvin, K. & Thomson, A. Meeting the radiative forcing targets of the representative concentration pathways in a world with agricultural climate impacts. *Earth's Future* **2**, 83-98, doi:10.1002/2013EF000199 (2014).
47 Hartin, C. A., Patel, P., Schwarber, A., Link, R. P. & Bond-Lamberty, B. P. A simple object-oriented and open-source model for scientific and policy analyses of the global climate system – Hector v1.0. *Geosci. Model Dev.* **8**, 939-955, doi:10.5194/gmd-8-939-2015 (2015).
48 Warszawski, L. *et al.* The Inter-Sectoral Impact Model Intercomparison Project (ISI–MIP): Project framework. *Proceedings of the National Academy of Sciences* **111**, 3228, doi:10.1073/pnas.1312330110 (2014).
49 Frieler, K. *et al.* Assessing the impacts of 1.5 °C global warming – simulation protocol of the Inter-Sectoral Impact Model Intercomparison Project (ISIMIP2b). *Geosci. Model Dev.* **10**, 4321-4345, doi:10.5194/gmd-10-4321-2017 (2017).
50 Calvin, K. *et al.* Global market and economic welfare implications of changes in agricultural yields due to climate change. *Climate Change Economics* **11**, 2050005, doi:10.1142/S2010007820500050 (2020).
51 Snyder, A., Calvin, K. V., Phillips, M. & Ruane, A. C. A crop yield change emulator for use in GCAM and similar models: Persephone v1.0. *Geosci. Model Dev.* **12**, 1319-1350, doi:10.5194/gmd-12-1319-2019 (2019).

52 Jones, J. W. *et al.* The DSSAT cropping system model. *European Journal of Agronomy* **18**, 235-265, doi:https://doi.org/10.1016/S1161-0301(02)00107-7 (2003).
53 Elliott, J. *et al.* The parallel system for integrating impact models and sectors (pSIMS). *Environmental Modelling & Software* **62**, 509-516, doi:https://doi.org/10.1016/j.envsoft.2014.04.008 (2014).
54 Portmann, F. T., Siebert, S. & Döll, P. MIRCA2000—Global monthly irrigated and rainfed crop areas around the year 2000: A new high-resolution data set for agricultural and hydrological modeling. *Global Biogeochemical Cycles* **24**, doi:10.1029/2008GB003435 (2010).
55 Bond-Lamberty, B. *et al.* On linking an Earth system model to the equilibrium carbon representation of an economically optimizing land use model. *Geosci. Model Dev.* **7**, 2545-2555, doi:10.5194/gmd-7-2545-2014 (2014).
56 Calvin, K. & Fisher-Vanden, K. Quantifying the indirect impacts of climate on agriculture: an inter-method comparison. *Environmental Research Letters* **12**, 115004, doi:10.1088/1748-9326/aa843c (2017).
57 Di Vittorio, A. V., Kyle, P. & Collins, W. D. What are the effects of Agro-Ecological Zones and land use region boundaries on land resource projection using the Global Change Assessment Model? *Environmental Modelling & Software* **85**, 246-265, doi:https://doi.org/10.1016/j.envsoft.2016.08.016 (2016).
58 Vernon, C. R. *et al.* A Global Hydrologic Framework to Accelerate Scientific Discovery. *Journal of Open Research Software* **7**, doi:https://doi.org/10.5334/jors.245 (2019).
59 Hejazi, M. I. *et al.* Integrated assessment of global water scarcity over the 21st century under multiple climate change mitigation policies. *Hydrol. Earth Syst. Sci.* **18**, 2859-2883, doi:10.5194/hess-18-2859-2014 (2014).
60 Liu, Y., Hejazi, M., Li, H., Zhang, X. & Leng, G. A hydrological emulator for global applications – HE v1.0.0. *Geosci. Model Dev.* **11**, 1077-1092, doi:10.5194/gmd-11-1077-2018 (2018).
61 Zhou, Y. *et al.* A comprehensive view of global potential for hydro-generated electricity. *Energy & Environmental Science* **8**, 2622-2633, doi:10.1039/C5EE00888C (2015).
62 Gernaat, D. E. H. J. *The role of renewable energy in long-term energy and climate scenarios*, Utrecht University, (2019).
63 de Vries, B. J. M., van Vuuren, D. P. & Hoogwijk, M. M. Renewable energy sources: Their global potential for the first-half of the 21st century at a global level: An integrated approach. *Energy Policy* **35**, 2590-2610, doi:https://doi.org/10.1016/j.enpol.2006.09.002 (2007).
64 Hoogwijk, M., de Vries, B. & Turkenburg, W. Assessment of the global and regional geographical, technical and economic potential of onshore wind energy. *Energy Economics* **26**, 889-919 (2004).
65 Köberle, A. C., Gernaat, D. E. H. J. & van Vuuren, D. P. Assessing current and future techno-economic potential of concentrated solar power and photovoltaic electricity generation. *Energy* **89**, 739-756, doi:https://doi.org/10.1016/j.energy.2015.05.145 (2015).
66 Zhou, Y., Luckow, P., Smith, S. J. & Clarke, L. Evaluation of Global Onshore Wind Energy Potential and Generation Costs. *Environmental Science & Technology* **46**, 7857-7864, doi:10.1021/es204706m (2012).
67 JGCRI. *GCAM v5.1 Documentation*, <http://jgcri.github.io/gcam-doc/v5.1/toc.html> (2019).
68 Denholm, P. & Margolis, R. Supply Curves for Rooftop Solar PV-Generated Electricity for the United States. Report No. NREL/TP-6A0-44073, (Golden, CO, 2008).
69 Grassi, G. *et al.* The key role of forests in meeting climate targets requires science for credible mitigation. *Nature Climate Change* **7**, 220-226, doi:10.1038/nclimate3227 (2017).
70 Forsell, N. *et al.* Assessing the INDCs' land use, land use change, and forest emission projections. *Carbon Balance and Management* **11**, 26, doi:10.1186/s13021-016-0068-3 (2016).

71 Binsted, M. *et al.* Stranded asset implications of the Paris Agreement in Latin America and the Caribbean. *Environmental Research Letters* (2019).
72 Iyer, G. *et al.* Measuring progress from nationally determined contributions to mid-century strategies. *Nature Climate Change* **7**, 871-874, doi:10.1038/s41558-017-0005-9 (2017).
