# Supplementary material for: Power sector investment implications of climate impacts on renewable resources in Latin America and the Caribbean
Source: Nat Commun. 2021 Feb 24;12:1276. doi: 10.1038/s41467-021-21502-y (PMC7904843; doi:10.1038/s41467-021-21502-y)
Supplement: Supplementary file 3 — Reporting Summary [file 41467_2021_21502_MOESM3_ESM.pdf]

nature research

Corresponding author(s): Silvia R. Santos da Silva

Last updated by author(s): Dec 10, 2020

Reporting Summary

Nature Research wishes to improve the reproducibility of the work that we publish. This form provides structure for consistency and transparency in reporting. For further information on Nature Research policies, see our Editorial Policies and the Editorial Policy Checklist.

Statistics

For all statistical analyses, confirm that the following items are present in the figure legend, table legend, main text, or Methods section.

n/a	Confirmed	
[ ]	[x]	The exact sample size (*n*) for each experimental group/condition, given as a discrete number and unit of measurement
[x]	[ ]	A statement on whether measurements were taken from distinct samples or whether the same sample was measured repeatedly
[x]	[ ]	The statistical test(s) used AND whether they are one- or two-sided<br>*Only common tests should be described solely by name; describe more complex techniques in the Methods section.*
[x]	[ ]	A description of all covariates tested
[x]	[ ]	A description of any assumptions or corrections, such as tests of normality and adjustment for multiple comparisons
[ ]	[x]	A full description of the statistical parameters including central tendency (e.g. means) or other basic estimates (e.g. regression coefficient) AND variation (e.g. standard deviation) or associated estimates of uncertainty (e.g. confidence intervals)
[x]	[ ]	For null hypothesis testing, the test statistic (e.g. *F*, *t*, *r*) with confidence intervals, effect sizes, degrees of freedom and *P* value noted<br>*Give P values as exact values whenever suitable.*
[x]	[ ]	For Bayesian analysis, information on the choice of priors and Markov chain Monte Carlo settings
[x]	[ ]	For hierarchical and complex designs, identification of the appropriate level for tests and full reporting of outcomes
[x]	[ ]	Estimates of effect sizes (e.g. Cohen's *d*, Pearson's *r*), indicating how they were calculated

Our web collection on statistics for biologists contains articles on many of the points above.

Software and code

Policy information about availability of computer code

Data collection	All data was collected from outputs from a global integrated assessment model. Specifically, scenarios developed for this study were simulated in the Global Change Analysis Model (GCAM). Although GCAM is an open source model, the version used in this study (GCAM-LAC) is a research branch whose source code is available at http://doi.org/10.5281/zenodo.4048788.
Data analysis	Analyses were mostly produced with Python versions 3.6 and 2.7. Common packages were used: Numpy, Pandas, Scipy, Matplotlib and Basemap (in python 2.7 to produce the maps). Capital investments were calculated using a script coded in the R program, version 3.6. Common R packages were used: readr, tidyr, dplyr and devtools. In addition, the rgcam package (available at https://github.com/JGCRI/rgcam) was also required.

For manuscripts utilizing custom algorithms or software that are central to the research but not yet described in published literature, software must be made available to editors and reviewers. We strongly encourage code deposition in a community repository (e.g. GitHub). See the Nature Research guidelines for submitting code & software for further information.

Data

Policy information about availability of data

All manuscripts must include a data availability statement. This statement should provide the following information, where applicable:

- Accession codes, unique identifiers, or web links for publicly available datasets
- A list of figures that have associated raw data
- A description of any restrictions on data availability

The data that support the findings of this study are available at https://github.com/Silviameteoro/GCAM-LAC_Modeling.

Field-specific reporting

Please select the one below that is the best fit for your research. If you are not sure, read the appropriate sections before making your selection.

- [ ] Life sciences
- [ ] Behavioural & social sciences
- [x] Ecological, evolutionary & environmental sciences

For a reference copy of the document with all sections, see nature.com/documents/nr-reporting-summary-flat.pdf

Ecological, evolutionary & environmental sciences study design

All studies must disclose on these points even when the disclosure is negative.

Study description	Overall description: This study focuses on the implications of climate change impacts on renewables (bioenergy, hydropower, solar and wind) for power sector investments under emissions constrained and unconstrained scenarios using a global integrated assessment model (GCAM-LAC).<br><br>Study design: This study relies on the mitigation and no_policy scenarios described in the article. These scenarios vary across assumptions about climate impacts on renewables and technology availability.<br><br>Number of model experiments: The climate inputs used to represent climate impacts on renewables in the GCAM model were produced from bias-corrected projections from the GFDL-ESM2M, HadGEM2-ES and IPSL-CM5A-LR general circulation models (GCMs) from the Inter-Sectoral Impact Model Intercomparison Project (ISIMIP) project under RCPs 2.6 and 6.0. We then conducted three distinct model simulations for each climate-impact scenario (described in the main text) corresponding to climate inputs from each of the GCMs. However, we focus on the mean values across all GCMs in the analysis.
Research sample	The study is based on the scenario outcomes of the GCAM model as described above. All scenarios simulated were used (1 set of scenario results per GCM climate inputs; mean values across GCMs calculated) to support our findings.
Sampling strategy	All data was collected from the GCAM model dependent upon scenario. GCAM provides outcomes at 5 year intervals from 2015 to 2100 (calibrated data prior to 2015). Original outcomes were aggregated to produce cumulative outcomes between 2020 and 2100 and 2020-2050 (Supplementary Information).
Data collection	Data was collected via GCAM model outputs by the corresponding author.
Timing and spatial scale	Timing: GCAM-LAC provides outputs at 5-yr time steps for 33 geopolitical regions. GCAM scenario outcomes were aggregated for the 2020-2100 period to produce cumulative outcomes of regional changes in cumulative generation and capital investments (other temporal aggregations - e.g. 2020-2050 - shown in the supplementary material).<br>Spatial scale: Latin America and the Caribbean region.
Data exclusions	No data was excluded from this study.
Reproducibility	The data of this study is available at https://github.com/Silviameteoro/GCAM-LAC_Modeling. The source code of the GCAM-LAC version used in this study is available at http://doi.org/10.5281/zenodo.4048788.
Randomization	Not applicable.
Blinding	Not applicable.

Did the study involve field work?
- [ ] Yes
- [x] No

Reporting for specific materials, systems and methods

We require information from authors about some types of materials, experimental systems and methods used in many studies. Here, indicate whether each material, system or method listed is relevant to your study. If you are not sure if a list item applies to your research, read the appropriate section before selecting a response.

Materials & experimental systems

n/a	Involved in the study	
[x]	[ ]	Antibodies
[x]	[ ]	Eukaryotic cell lines
[x]	[ ]	Palaeontology and archaeology
[x]	[ ]	Animals and other organisms
[x]	[ ]	Human research participants
[x]	[ ]	Clinical data
[x]	[ ]	Dual use research of concern

Methods

n/a	Involved in the study	
[x]	[ ]	ChIP-seq
[x]	[ ]	Flow cytometry
[x]	[ ]	MRI-based neuroimaging
